# High-throughput screening of natural compounds and inhibition of a major therapeutic target HsGSK-3*β* for Alzheimer’s disease using computational approaches

**DOI:** 10.1186/s43141-021-00163-w

**Published:** 2021-05-04

**Authors:** Rohit Shukla, Tiratha Raj Singh

**Affiliations:** Department of Biotechnology and Bioinformatics, Jaypee University of Information Technology (JUIT), Waknaghat, Solan, H.P., 173234 India

**Keywords:** Alzheimer’s disease, Neurofibrillary tangles, Tauopathies, GSK-3*β*, Virtual screening, Molecular docking, Molecular dynamics simulation, Principal component analysis, MM-PBSA

## Abstract

**Background:**

Alzheimer’s disease is a leading neurodegenerative disease worldwide and is the 6th leading cause of death in the USA. AD is a very complex disease and the drugs available in the market cannot fully cure it. The glycogen synthase kinase 3 beta plays a major role in the hyperphosphorylation of tau protein which forms the neurofibrillary tangles which is a major hallmark of AD. In this study, we have used a series of computational approaches to find novel inhibitors against GSK-3*β* to reduce the TAU hyperphosphorylation.

**Results:**

We have retrieved a set of compounds (*n*=167,741) and screened against GSK-3*β* in four sequential steps. The resulting analysis of virtual screening suggested that 404 compounds show good binding affinity and can be employed for pharmacokinetic analysis. From here, we have selected 20 compounds those were good in terms of pharmacokinetic parameters. All these compounds were re-docked by using Autodock Vina followed by Autodock. Four best compounds were employed for MDS and here predicted RMSD, RMSF, Rg, hydrogen bonds, SASA, PCA, and binding-free energy. From all these analyses, we have concluded that out of 167,741 compounds, the ZINC15968620, ZINC15968622, and ZINC70707119 can act as lead compounds against HsGSK-3*β* to reduce the hyperphosphorylation.

**Conclusion:**

The study suggested three compounds (ZINC15968620, ZINC15968622, and ZINC70707119) have great potential to be a drug candidate and can be tested using in vitro and in vivo experiments for further characterization and applications.

**Supplementary Information:**

The online version contains supplementary material available at 10.1186/s43141-021-00163-w.

## Background

Alzheimer’s disease (AD) majorly contributes to dementia and is a lethal neurodegenerative disease. Worldwide approximately 50 million people are suffering from some form of dementia in which AD is the most contributing disease (60–70%). It is the major cause of death in the USA and is ranked 6th in number. According to the World Alzheimer Report of WHO 2018, this number (50 million) for dementia will be tripled (~152 million) by the year 2050, which reflects the seriousness of this disease for mankind. The disease showed a very high economic burden on the global society as 1 trillion US$ loss in 2018 was observed while it will double till 2030 [1]. The impact of the disease is reflected by its rise in incidence rate where one person develops dementia every 3 second globally. There is an urgent need to find treatment and cure for this disease. AD showed very complex disease etiology which is characterized by majorly two hallmarks, first is the association of amyloid β plaques which is formed by the abnormal cutting of amyloid precursor protein (APP) while another hallmark is the neurofibrillary tangles (NFTs) being formed by the association of hyperphosphorylated microtubule-associated binding protein (MAPT) [2, 3]. AD is also linked with several autosomal mutations in the genes which are inherited from parents encoding APP, tau protein, and presenilins 1 and 2 (PSEN1 and PSEN2), and these mutations induce the Aβ plaques and NFTs formation [4–7].

For understanding the disease mechanism and inhibitor identification, several studies have been done recently [8–10]. Quite a lot of efforts have been made towards finding the cure for AD for the last 20 years against Aβ-based therapeutics including drug identification, and antibody generation but these are not successful [1]. Scientists also looked towards other tau-induced therapies for AD such as tauopathies. The tau is a microtubule-stabilizing protein that stabilizes the microtubule and binds with α and β tubulin units of microtubules and forms the nerve cell cytoskeleton. During the Aβ formation and several other conditions, various kinases like GSK-3*β* (glycogen synthase kinase 3 beta), CDK5 (cyclin-dependent kinase 5), DYRK1A (dual specificity tyrosine-phosphorylation-regulated kinase 1A), and few more enzymes hyperphosphorylate the tau protein [11]. Due to the hyperphosphorylation, tau detached from the microtubule and aggregates in the form of clumps of intracellular NFTs, which block the nerve cell communication and contributes towards AD progression. The NFTs are the result of assembled tau protein fragments, and they can also disrupt the nuclear-cytoplasmic transport [12]. Several compounds are in a clinical trial for reducing the NFTs aggregation. But there is an urgent need for inhibitors which can reduce tau hyperphosphorylation by inhibiting the kinases which are responsible for hyperphosphorylation [11].

The GSK-3*β* is an enzyme belonging to the family of proline-directed serine/threonine kinase and plays a vital role in the phosphorylation of various substrates in a range of pathways [13]**.** This enzyme is involved in the regulation of various cellular processes like metabolism, cardiac hypertrophy, cell proliferation, apoptosis, and oncogenesis [14]. It is known as a major therapeutic target against various metabolic disorders like insulin resistance and type-2 diabetes because its function is associated with glycogen metabolism. The GSK-3*β* is associated with several central nervous systems (CNS) diseases like AD, stroke, and Huntington’s disease due to its overexpression in the brain [15, 16]. Various strong evidence in the literature showed that the GSK-3*β* co-localizes preferentially with neurofibrillary tangles [17]. GSK-3*β* expressed actively in pre-tangle neurons and plays an active role in the formation of paired helical filaments (PHFs) or NFTs in the AD patient brain [18, 19]. Alteration in the GSK-3*β* function induces various neuroinflammation and neurodegenerative disorders which affect the CNS. The hyperphosphorylation occurs in the PHFs due to the over activation of the GSK-3*β*, and it is revealed both in transfected mammalian neuronal cells and in vivo experiments [14]. The role of GSK-3*β* is mainly involved in the tau protein phosphorylation while it is also associated with some other AD-related mechanisms. Various strategies have been applied to find novel inhibitors against GSK-3*β* to reduce the phosphorylation burden of tau and for the management of tauopathies [20, 21].

**Fig. 1:**
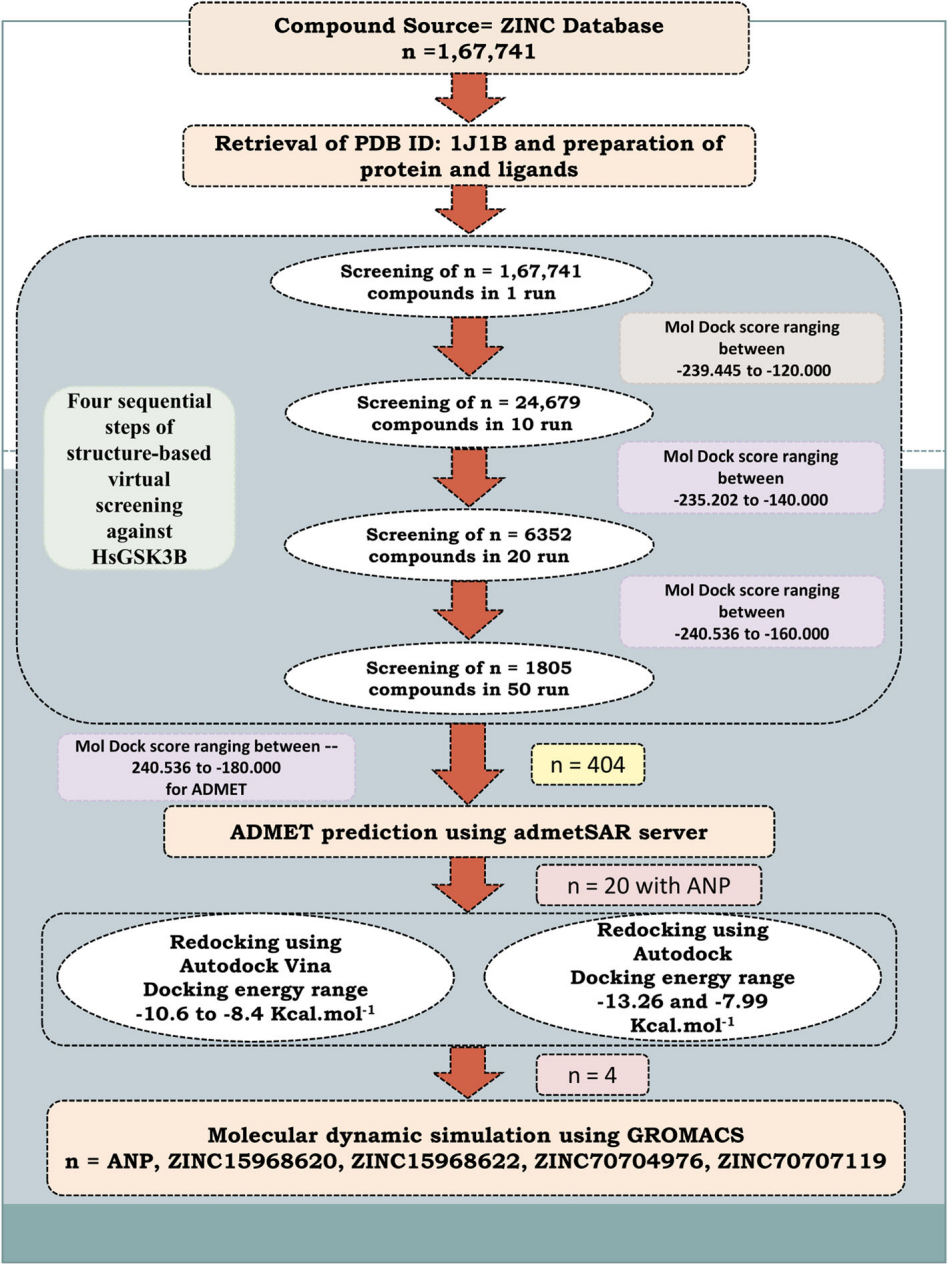
Schematic workflow for identifying the potential drug-like molecules specific to the HsGSK3β.

Here we have applied the computational high throughput screening method to predict the potential inhibitors against GSK-3*β*. A natural subset (*n*=167,741) was retrieved and screened in four steps of virtual screening against GSK-3*β*. Based on the MolDock score the 404 compounds were selected and employed for ADMET prediction. The 20 compounds were selected based on pharmacokinetics evaluations along with ATP analog ANP (PhosphoAminophosphonic acid-adenylate ester) for re-docking by using Autodock Vina and Autodock software. Based on re-docking, we have selected finally four compounds that showed a greater binding affinity than the control compound and employed them for molecular dynamics simulation (MDS) study. Various MDS results were performed and analyzed like RMSD, RMSF, radius of gyration, number of hydrogen bonds, PCA, and binding-free energy. The analysis suggests that three compounds can act as best lead molecules against GSK-3*β* and can act as an anti-Alzheimer compound or could be proposed as potential lead molecules for tauopathies. The whole method which has been used in this study was depicted in Fig. 1.

## Methods

### Receptor and ligand preparation

The 3D crystal structure of GSK-3*β* was retrieved from the RCSB protein databank. Various crystal structures (approximately >35) are available in PDB with good resolution. From there, we have selected GSK-3*β* (RCSB-PDB ID: 1J1B, X-ray, 1.8 Å) [22] which is co-crystallized with ANP an analog ATP. Here we wanted to design the ATP competitive inhibitor therefore ANP was a good control for this study. 1J1B was imported into Chimera 1.13.2 [23], and all the hydrogens were added by using a structure preparation wizard. There are several open-source protein preparation tools available while Chimera 1.13.2 has multiple advanced options than other tools such as structure editing, multiple force-fields. The structure minimization was performed using Amber ff99SB force-field through the Amber tool of Chimera 1.13.2 [23]. This force-field is widely used to perform the MD simulation and energy minimization of the structure. The natural compound subset (*n*=167,741) is downloaded from the ZINC database [24–26]. The compounds are categorized based on the ZINC natural product catalog. All structures of small molecules were retrieved in *.mol2* file format which has all the hydrogen atoms.

### ANP binding analysis

In the ANP, only one oxygen is replaced by nitrogen for making it an ATP competitive inhibitor. The ANP is a control compound in this study. Here ANP binds between the two domain interface: one domain has β strand (residues 35-138) and the other consisting of a helical domain with residues from 139 to 388 and a loop (glycine-rich) and hinge region surrounds the cavity [22]. The hydrophobic binding cavity of the enzyme occupies the adenine ring of ANP and showed the interaction with the main chain atoms of the hinge loop. Here the authors described that a key residue (Arg141) mainly involve in the ANP binding and it is not present in other serine-threonine kinases [22]. Hence, Arg141 is used as a centroid residue due to its specificity for the grid preparation.

### Virtual screening

Drug identification, design, and development is a very time-consuming and expensive procedure, while with the advent of new computational technologies, the drug identification becomes a little easy as well as less time-consuming. The in silico drug identification method can screen the large databases based on binding site information or the basis of pharmacophore or QSAR model and can predict the lead compound to reduce the time for the drug identification [27]. This process is faster, has a low cost, and reduces the burden of unnecessary experiments [28]. The structure-based virtual screening approach is a popular and established method for the screening of datasets retrieved from the ZINC database [24]. The ligands and receptors were prepared using the Molegro Virtual Docker (MVD) [29] and screened in four steps using MVD. The MVD is a protein-ligand docking software and used for the screening of large datasets as mentioned earlier [30, 31]. The MolDock Score is provided in the form of a binding score. Then four subsequent runs were performed to find the novel inhibitors from the virtual screening. In the first run, a total of *n*=167,741 natural ligands were used for virtual screening in one run. Other parameters were set like population size (50), maximum iteration (2000), scaling factor (0.50), and crossover rate (0.90). From the first round of docking, we have selected 24,689 compounds for the next round of virtual screening. The same parameter was used for the next round except for the number of runs which was increased from 1 to 10. Then from here 6352 compounds were selected and screened in 20 runs. Finally, 1805 compounds were selected from the previous result and screened in 50 runs. Then finally, out of 1805 compounds, 404 compounds were selected for ADMET (Absorption, Distribution, Metabolism, Excretion, and Toxicity) prediction. Although this is a well-proved [27, 30, 31] and a good method for virtual screening where it has some drawbacks also, in the 1 run, sometimes it can exclude the good compounds but this error can also be removed by screening all the compounds in 100 runs with good computational facility. Some other methods are also available in which users can screen a large library by using the Autodock Vina and other software [32–34]. In the Autodock Vina, the user can increase the exhaustiveness instead of the number of runs and then can perform the screening. The Autodock Vina is a command-line tool while MVD is a GUI-based tool where users can easily give a library of compounds and can screen in multiple steps. Hence, screening by using the MVD is better than Autodock Vina and other command-line tools. We have also used Autodock Vina for redocking in this study.

### Pharmacokinetic evaluation

The ADMET parameters are very important when we are proposing the lead compound. The ADMET values predictions by using in silico tools can reduce the time and give a proper estimation of the pharmacokinetic behavior of a predicted small chemical molecule. Several tools such as preADMET, pkCSM, and others are available to predict the ADMET values but we have used the admetSAR online server (http://lmmd.ecust.edu.cn/admetsar1/) [35] for the calculation of pharmacokinetic parameters (ADMET). It predicts more and relevant descriptors as compare to other tools. Additionally, the developers are regularly updating this server. It uses the data of FDA approved drugs and predicts the values for the newly given compound using the machine learning algorithm. The data has also been taken from Google Scholar (https://scholar.google.co.in/) and Pubmed (https://www.ncbi.nlm.nih.gov/pubmed/). We have predicted various parameters like BBB (blood-brain barrier), Caco-2 cell permeability, HIA (Human Intestinal Absorption), Pgp Substrate/Inhibitor identification, Carcinogenicity, toxicity, Cyp450 metabolism, hERG gene inhibition, and Lethal dose. We have employed all the selected 404 compounds for ADMET prediction, and several descriptors were calculated. From this analysis, we have selected 20 compounds that were employed for re-docking studies.

### Molecular docking

For the prediction of better binding poses as well as for validating our selected ligands, we have re-docked all selected 20 compounds including ANP by using three widely used software’s Molegro Virtual Docker [29], Autodock Vina [36], and Autodock [37]. The number of runs is increased to 100 for MVD and a total of 5 poses is generated for each ligand. The grid box for the docking with Autodock Vina, and Autodock was set based on co-crystallized ligand ANP and kept the same which were used in the MVD virtual screening process. Here we have focused on Arg141 because this is not conserved in other kinases, as mentioned earlier [22]. The MGL tools provide a complete suite to perform the protein-ligand preparation, grid preparation, etc. Then for docking, we have used Autodock which is a freely available and highly cited tool for protein-ligand docking. The grid box is generated by using the grid generation box of Autodock Tools. We used ANP inhibitor as a reference to construct the size of the grid (X, Y, and Z-coordinates as 50, 50, and 50 Å, respectively) with 0.4Å grid spacing, where the incoming ligand binds during the docking process. First Autodock Vina was used for re-docking studies. It is a very efficient and less time-consuming software than Autodock. Then to crosscheck and finally select the best results, we used Autodock. The Autodock uses the Lamarckian genetic algorithm conformational search method for the generation of binding poses. The semi-empirical free energy force field was used with all the default parameters except pose generation. The sum of the freedom of a torsional degree was used for the calculation of the conformational entropy. The binding energy was evaluated in two steps firstly, the protein and ligand energy were calculated in the unbound state, and then in the second step, the protein-ligand complex energy was calculated [38]. Finally, the difference between 1 and 2 was considered for the result.

1 $$ \Delta G=\left({V}_{bound}^{L-L}-{V}_{unbound}^{L-L}\right)+\left({V}_{bound}^{P-P}-{V}_{unbound}^{P-P}\right)+\left({V}_{bound}^{P-L}-{V}_{unbound}^{P-L}+\Delta {S}_{conf}\right) $$

In this equation, the *protein is referred to by P*, *Ligand is referred by L*, *pairwise evaluation is denoted by V*, and ΔS_conf_ denotes the loss of conformational entropy during binding. For each ligand, 100 binding poses were generated for result analysis. Then, these generated binding poses were sort-listed based on binding affinity as well as the number of hydrogen bonds.

### Conformational stability analysis

The conformational changes and complex stability can be best achieved by the MDS [39, 40]. It is a very useful and widely accepted method to predict the accuracy of docking poses [41]. The complexes were selected based on interaction and binding affinity analyses which were carried forward for the MDS analysis. The apo-HsGSK3β and predicted complex (HsGSK3β-ANP (control compound), HsGSK3β-ZINC15968620, HsGSK3β-ZINC15968622, HsGSK3β-ZINC70704976, and HsHSK3β-ZINC70707119) were employed for 60 ns molecular dynamics simulation studies by using Gromacs 5.1.2 [42] for analyzing the protein-ligand stability. The Gromacs is an open-source widely usable software for the MDS. It has several force-fields such as Amber, GROMOS, and CHARMM. with regular updates. However, it cannot generate the topology other than 20 amino acids, hence we have to use third-party software to generate the ligand topology such as ProDRG. The generation of each conformation with the time frame is based on Newton’s law of molecular motions (Eq. 1).

2 $$ {F}_i={m}_i{a}_i=\frac{\delta V\left({r}^N\right)}{\delta {r}_i} $$

Here *i* represents the mass of the atom while *m*_*i*_ and *a*_*i*_ represent its acceleration. The force acting on *i* given by the partial spatial derivative of the potential energy function *V* that is dependent on the positions *r*^N^ = (*r*^1^, *r*^2^, … , *r*^N^) of all *N* particles in the system is represented by *F*_*i*_. The protein topology and ligand topology were generated by using the GROMOS 9653A6 force field [43]. The ligand coordinates were generated by using the ProDRG server [44]. A cubic box is created and then HsGSK3β and HsGSK3β-ligand complexes were placed in that box. Solvent molecules (no. 28,101) were added and 8 Cl^-^ ions were also added for neutralizing the systems. Energy minimizations of all the systems were performed to remove the steric clashes of the system. Then the PBC (periodic boundary condition) was applied and the Ewald summation method [45] was used for the long-range electrostatic interaction calculation. After that NVT (the constant number of particles, volume, and temperature), simulation was performed to fix the volume and temperature (300K) of the systems. Then NPT (the constant number of particles, pressure, and temperature) simulation was carried out to fix the pressure of the system. Finally, all the systems were employed for the 60 ns MDS study, and the coordinates were saved in 2fs. Then all the trajectories were analyzed with various Gromacs utilities. The gmx rms, gmx rmsf, gmx gyrate, gmx sasa, and gmx hbond tools were used for the calculation of various parameters like RMSD, RMSF, Rg, SASA, and number of hydrogen bonds. The trajectories were visualized and analyzed by using Chimera 1.13.2 [23].

### Binding-free energy calculation

The g_mmpbsa developed by Kumara et al. is a commonly used tool to calculate the binding-free energy using Gromacs as an interface [46]. This is a very easy-to-use tool as compare to others. The user can calculate the binding free energy by using only two steps. The last 10 ns MD trajectory snapshots were used for the calculation of binding free energy. The ΔG_bind_ is calculated by the following equation:

3 $$ {\Delta \mathrm{G}}_{\mathrm{bind}}={\Delta \mathrm{G}}_{\mathrm{mm}}+{\Delta \mathrm{G}}_{\mathrm{sol}}-\mathrm{T}\Delta \mathrm{S} $$

The electrostatic and Van der Wall interactions were computed in the molecular mechanics energy (ΔG_mm_). The polar and non-polar contributions defined solvation free energy (ΔG_sol_). The Solvent Accessible Surface Area (SASA) model was used for the determination of nonpolar solvation-free energy. In this method, the entropy (-TΔS) is not calculated due to the high computational cost.

## Results

### Virtual high-throughput screening

For the identification of potential drug candidates that can inhibit the activity of HsGSK3β, a systematic structure-based virtual screening approach was implemented. The crystal structure of HsGSK3β was used for virtual screening. It is co-crystallized with the ANP. So we have set a binding site based on the control compound ANP and then docked this compound first. Then all the retrieved ligand compounds (*n*=167,741) were screened against this enzyme in the defined grid. From the first round of virtual screening, 24,689 compounds were selected which showed the MolDock Score ranging between −239.45 and −120.00. These compounds were again screened in 10 runs. Increasing the number of runs can remove false-positive binders [27]. The 6352 compounds were selected between the MolDock Score −235.20 and −140.00 from this round of screening. Then in the last run, we have selected 1805 compounds and screened in 50 runs. These showed the MolDock Score of −240.54 to −160.00. Then from this round of virtual screening, it was observed that many of these natural compounds reflect a good binding affinity score towards the binding pocket of HsGSK3β; therefore, these compounds (*n*=404) were selected for the pharmacokinetic evaluation.

### Pharmacokinetic evaluation

The natural compounds selected from the previous analysis (404) were subjected to an admetSAR tool for the evaluation of various pharmacokinetic descriptors like BBB, HIA, Caco-2 cell permeability, Cyp450 substrate/inhibition, Pgp-substrate/inhibition, carcinogenicity, hERG gene inhibition, and Lethal dose. As we are targeting the central nervous system (CNS), so we have selected only those compounds for further study which can cross the BBB. We found that out of 404 compounds, 269 compounds were showing permeability towards the BBB. HIA describes the absorption of a small molecule in the large intestine. 367 compounds could be absorbed from the HIA from our dataset of 404 compounds. In the Caco-2 parameter, we could not found more compounds that can pass this parameter like out of 404, 361 cannot pass from these criteria while 41 compounds can pass from these criteria. In our study, only 43 compounds can pass from this filter. P-gp (P-glycoprotein) is a cell surface receptor for the efflux of the xenobiotics. Out of 404 compounds, the 315 and 89 compounds acting as a substrate and non-substrate towards P-gp, respectively. Out of 404, the 168 and 236 compounds acted as a substrate and non-substrate towards P-gp, respectively (Supplementary Table S1). The Cytochrome P450 is the key enzyme for the metabolization of the chemical molecule or xenobiotics. In this study, we have taken 5 variants of Cyp450 and predicted the inhibition and substrate probability. In our study, 176 and 228 compounds act as high and low inhibition promiscuity against Cyp450, respectively (Supplementary Table S2). Further, toxicity, carcinogenicity, and other parameters filtering were also done. The toxic compound cannot be selected because it can cause adverse effects when entering into the bloodstream. So we have removed those compounds which showed the toxicity. In this study, only 31 compounds act as toxic compounds out of 404 compounds. Carcinogenicity of the compound described that if the compound can cause the mutation. So here only 2 compounds act as carcinogenic compounds while others are safe in this parameter. We have also predicted hERG gene inhibition because inhibition of this gene can cause long QT syndrome. In our study, 269 compounds act as a non-inhibitor while 135 compounds act as an inhibitor for this gene. Lastly, we have predicted the lethal dose and acute oral toxicity of all the compounds. The LD_50_ of maximum compounds came between 2 and 3 mol.Kg^-1^ (Supplementary Table S3). From all these analyses, we have selected only 20 compounds out of 404 compounds that passed all the parameters successfully and were subjected for re-docking studies.

### Molecular docking analysis

The re-docking study is a good approach to predict the binding poses from various tools and methods for validating the study. Based upon the technical evaluation from the previous set of analyses, we have taken 20 compounds along with the control compound ANP for re-docking studies. We have used Autodock Vina, Autodock, and MVD for this purpose. The ANP showed binding affinity through Autodock, Autodock Vina, and MVD and its scores are −7.8, −7.83, Kcal.mol^-1^, and −179.07 MolDock Score, respectively. From the Autodock, we have seen ZINC70707119 and ZINC95100194 showed the highest and lowest binding affinity of −13.26 and −7.99 Kcal.mol^-1^. The binding affinity for 20 compounds ranging between −13.26 and −7.99 Kcal.mol^-1^ from ADT. The ZINC15968620 and ZINC95100194 showed the higher and lower binding affinity of −10.6 and −8.4 Kcal.mol^-1^ from the Autodock Vina. The binding affinity ranging between −10.6 and −8.4 Kcal.mol^-1^ for all the 20 compounds. The MVD, ZINC70704976, and ZINC70700682 showed the highest and lowest MolDock Score of −207.04 and −181.85, respectively. In the case of MVD, we have seen the MolDock Score ranging from −207.04 to −181.25. The binding affinity, number of hydrogen bonds, and interacting residues for all the 20 compounds with control compound ANP were shown in Supplementary Table S4.

### Structural analysis of selected hits

**Fig. 2:**
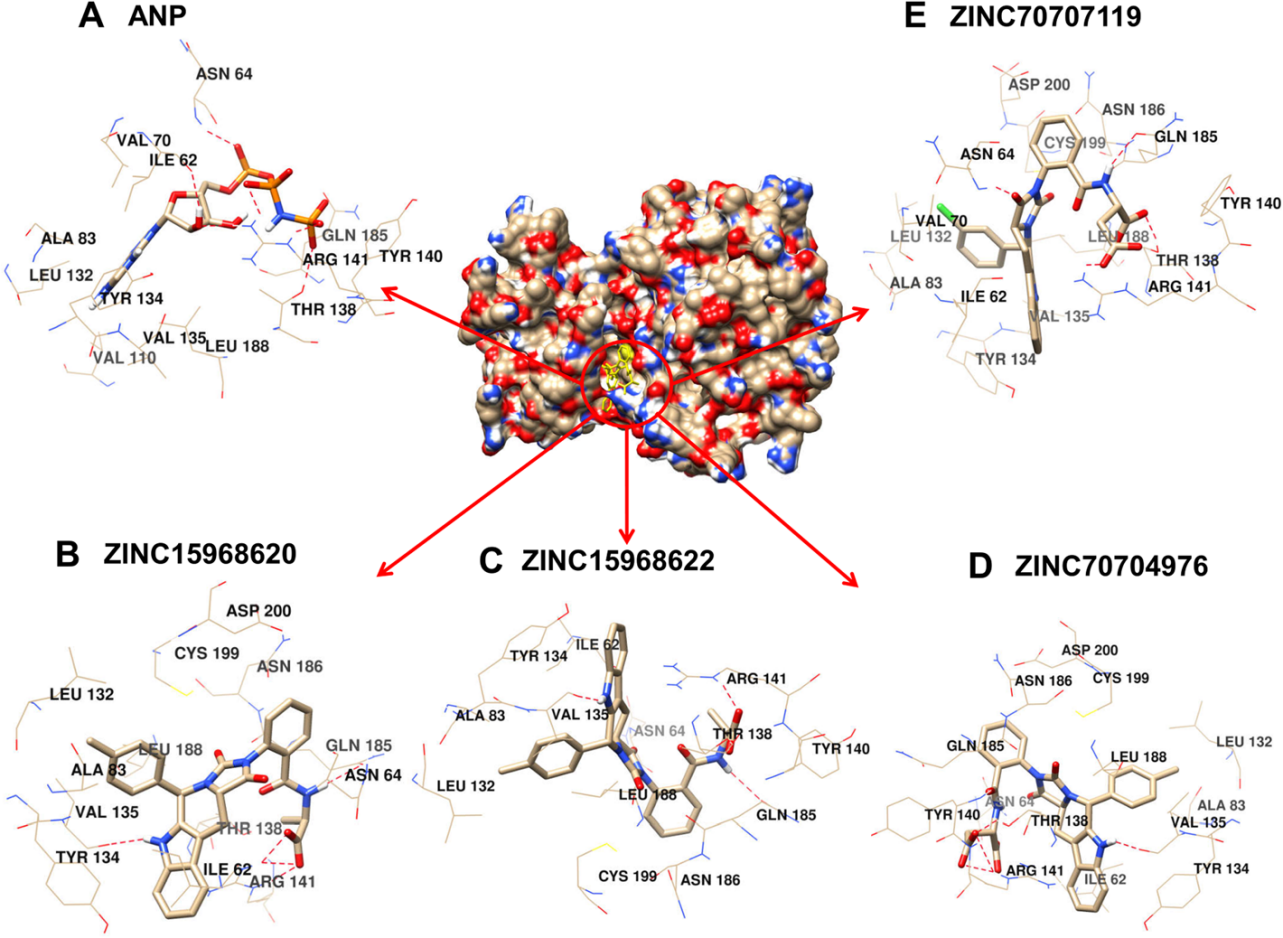
Schematic representations of the binding interactions with HsGSK3β. **a** HsGSK3β-ANP. **b** HsGSK3β-ZINC15968620. **c** HsGSK3β-ZINC15968622. **d** HsGSK3β-ZINC70704976. **e** HsGSK3β-ZINC70707119

The binding pattern of the four selected (ZINC15968620, ZINC15968622, ZINC70704976, ZINC70707119) hits is described below, which was selected by rounds of virtual screening as well as ADMET studies from 167,741 compounds. The ANP binding pattern was also checked for validation of the docking study. The chemical structure, binding affinity, interacting residues for four selected hits, and ANP were shown in Table 1 and Fig. 2.

**Table 1:**
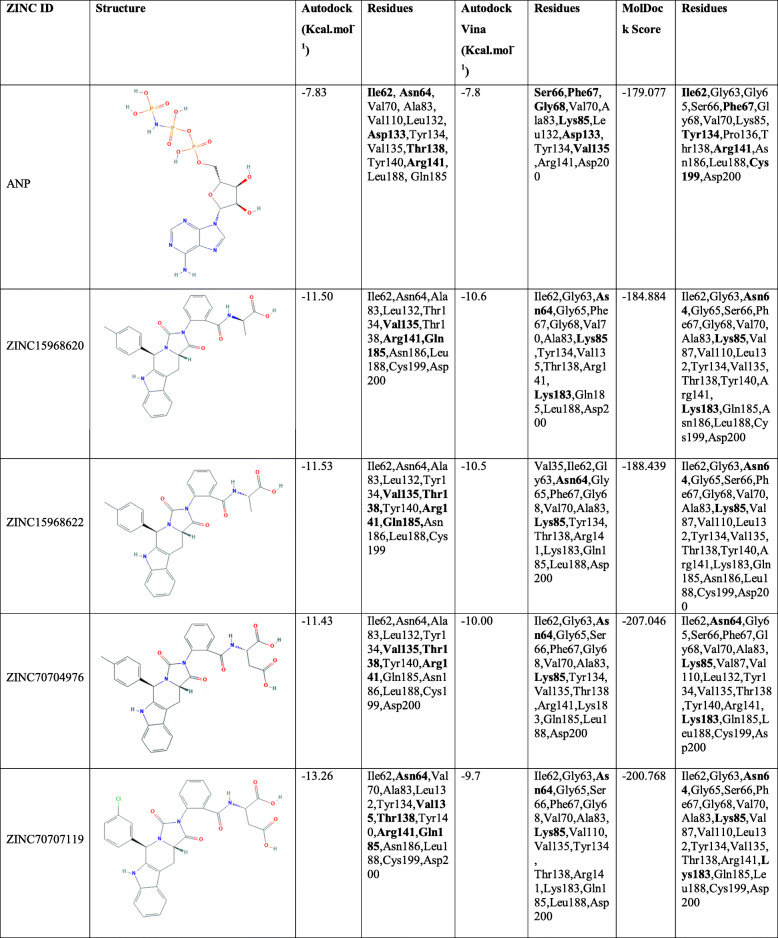
Details of the four selected compounds with control compound ANP. ZINC ID, structures, binding affinity, and interacting residues obtained after molecular docking are shown. The residues which are forming hydrogen bonds are shown in bold

### Docking protocol validation

The ANP is co-crystallized in the PDB structure (PDB ID: 1J1B). The ANP is removed from the PDB and again re-docked by all three docking tools. The binding residues are found similar from all the three docking tools while binding affinity is different due to different algorithms. The RMSD of the ligands from all the docking tools is <2 Å from the experimentally solved 3D coordinates. Various residues that interact in the PDB structure like Asp133 and Val135 are the same and forming the hydrogen bonds in re-docking studies. The PDB structure authors resolved that Arg141 is a conserved residue, and it showed the interaction from all three docking tools [22]. The binding affinity for ANP from all the three tools was −7.83, −7.8 Kcal.mol^-1^, and −179.077 MolDock Score. Table 1 and Fig. 2a show detailed analyses of interacting residues and binding affinity. The common residues which lie from all three softwares are Val70, Tyr134, and Arg141. In these three residues, the Val70 and Arg141 are also predicted by an experimentally solved (PDB ID: 1J1B) structure.

#### ZINC15968620

It is the predicted hit, which showed the highest binding affinity from Autodock VINA as compared to all other ligands. The binding affinity from Autodock Vina, Autodock, and MVD was shown −10.6, −11.5 Kcal.mol^-1^, and −184.884 MolDock Score. The binding affinity of this compound is greater than ANP. The binding score represents that it has more affinity than ANP and can block the active site of GSK3β and inhibits the enzyme’s function. It binds with the key catalytic residues of the ATP site. Table 1 and Fig. 2b showed detailed analyses of interacting residues, binding affinity, and the number of hydrogen bonds. Common interacting residues, which have been predicted from all three docking tool’s are Ile62, Asn64, Ala83, Val135, Thr138, Arg141, Gln185, Leu188, and Asp200. Here we have seen that most of the amino acid residues are hydrophobic and positively charged. It describes that the binding site has the capability of hydrophobic interactions. Arg141 is also conserved and it is a key residue that is not conserved in other kinases and actively participating in GSK3β-ZINC15968620 complex stabilization. In these residues Val135, Arg141, Gln185, and Asp200 are actively participating in the ANP stabilization in the experimentally resolved structure also. It represents that our ligand is interacting with the key residues which are required for the activity of the enzyme and the docking tools are giving accurate binding poses. Additionally, newly identified residues could also be checked experimentally and can provide new substrate specificity.

#### ZINC15968622

It is the derivative of ZINC15968620 with a bonding difference. It showed approximately similar binding affinity which was shown by ZINC15968620. It showed the binding affinity from Autodock Vina, Autodock, and MVD as −10.5, −11.53 Kcal.mol^-1^, and −188.439 MolDock Score, respectively. It also showed a greater binding affinity than control ligand ANP. Binding affinity proves that it can compete with the ANP and occupy the ATP binding site and can block the enzyme function. It showed good hydrogen and other bonding interactions with the key catalytic residues. It showed the binding with Arg141 through two of the three software used. A detailed description of the chemical structure, hydrogen bonding, and binding affinity is given in Table 1 and Fig. 2c. The identical residues from all three docking tools were predicted and compared with the X-ray crystallized structure. The residues Ile62, Asn64, Ala83, and Leu188 were found common from all three docking tools.

#### ZINC70704976

The ZINC70704976 also showed a good binding affinity and even greater than ANP from all the three docking tools. The binding affinity of −10.0, −11.43 Kcal.mol^-1^, and −207.046 MolDock Score was observed from Autodock Vina, Autodock, and MVD tools, receptively. It showed the highest MolDock Score compared to all other compounds. The compounds showed the interaction with the key catalytic residues. The number of hydrogen bonds, binding affinity, and the chemical structure is shown in Table 1 and Fig. 2d. The residues, which were identified from all the docking tools, are Asn64, Ile62, Ala83, Tyr134, and Val135. These all residues are catalytically important. The Val135 is a residue that is also found in the stabilization of ANP with HsGSK3β in the experimentally resolved structure.

#### ZINC70707119

The ZINC70707119 showed the highest binding affinity of −13.26 Kcal.mol^-1^ from the Autodock as compared to all other selected ligands. The ZINC70707119 also showed a greater binding score than ANP. The binding score from Autodock Vina was −9.7 while the −200.768 MolDock score was observed from the MVD. It interacts with the various key catalytic residues like Arg141 which is a unique residue of GSK3β and participates in the hydrogen bonding during docking. A detailed description of hydrogen bonds, hydrophobic interaction, and chemical structure is given in Table 1 and Fig. 2e. The residues found identical from all the docking tools are Ile62, Asn64, Val70, Ala83, Tyr134, Val135, Thr138, Arg141, Gln185, Leu188, and Asp200. These are the key catalytic residues and are also found in ANP interaction in the crystal structure. The residues Val70, Val135, and Asp200 are identical to the experimentally solved structure. From this analysis, we have seen that all four ligands have a higher binding affinity than the control ligand ANP. It represents that all selected hits can compete with the ANP and can occupy the binding cavity. These residues are interacting with the key catalytic residues which are required for enzyme function. It reveals that our predicted inhibitors may alter the enzyme function. Further MDS study was performed for the prediction of conformational dynamics of all the four compounds with ANP complex and apo-HsGSK3β.

#### Common scaffold for all four selected compounds

We obtained a very interesting finding from this analysis. We have worked on a structure-based virtual screening approach with a set of compounds. From these rounds of virtual screening, we have selected 4 compounds for the MDS study. In these compounds, we have seen that they belong to one scaffold (β-carboline-hydantoin derivative named “2,5-DIPHENYL-1,3-DIOXO-6H-1,2,3,5,11,11A-HEXAHYDROIMIDAZO-[1,5-B]-BETA-CARBOLINE”) [47] only and all the compounds are derivatives of this scaffold. The chemical structure is shown in Supplementary Figure 1. It represents that this scaffold can play a key role in the designing of potent inhibitors against GSK3β for AD and other tauopathies.

### Molecular dynamic simulation

The MDS was performed in two phases. First, the systems are prepared to remove the steric clashes and the temperature and volume of the system are fixed, then second the production run of 60 ns was carried out for predicting the protein-ligand complex stability analysis. The MDS provides the deeper detail of the mechanism of the ligand-binding, and we can predict the changes at the atomic level with the time-scale. We have predicted various values such as RMSD, RMSF, Rg, SASA, number of hydrogen bonds, PCA, and binding-free energy for predicting the protein-ligand complex stability. The trajectories are equilibrated after 20 ns, hence the analysis was carried out from the last 40 ns trajectory.

### RMSD

**Fig. 3:**
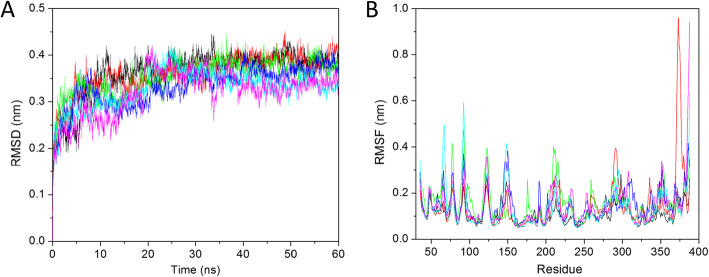
Molecular dynamic simulation. **a** Root mean square deviation (RMSD) values of Cα backbone atoms for 60 ns. **b** Root mean square fluctuation (RMSF) values of Cα atoms for the last 40 ns. The color code for all panels are apo-HsGSK3β (black), HsGSK3β-ANP (red), HsGSK3β-ZINC15968620 (green), HsGSK3β-ZINC15968622 (blue), HsGSK3β-ZINC70704976 (cyan), and HsGSK3β-ZINC70707119 (magenta)

The RMSD is a widely used parameter to check the stability of the system. Here the RMSD value is calculated for all the systems to predict the conformational changes after ligand binding in the apo-protein and depicted in Fig. 3a. The average RMSD value for apo-HsGSK3β, HsGSK3β-ANP, HsGSK3β-ZINC15968620, HsGSK3β-ZINC15968622, HsGSK3β-ZINC70704976, and HsGSK3β-ZINC70707119 were 0.38, 0.38, 0.37, 0.35, 0.35, and, 0.34 nm, respectively, for the last 40 ns trajectory. From the RMSD value, we have analyzed that the ligand-binding induces stability in the apo-HsGSK3β. The apo-HsGSK3β and HsGSK3β-ANP showed the same RMSD value while HsGSK3β-ZINC70707119 showed the least RMSD value as compared to all other compounds. All the trajectories got the equilibration state after 20 ns while some extent in the RMSD peak was observed in the HsGSK3β-ZINC70707119. It showed the lower and higher RMSD peak till 40 ns, and after that, it got the equilibration state and showed a stable peak. From the RMSD, we have concluded that all the trajectories are well stable and can be considered for further analysis.

#### Residue mobility analysis

The RMSF defines residual mobility after ligand binding. The rigid structure like helix and sheet showed lower RMSF values while loosely folded structures like turns and coils showed higher RMSF values. We have calculated the RMSF value for all the ligand complexes and shown in Fig. 3b. The average RMSF value for apo-HsGSK3β, HsGSK3β-ANP, HsGSK3β-ZINC15968620, HsGSK3β-ZINC15968622, HsGSK3β-ZINC70704976, and HsGSK3β-ZINC70707119 were 0.11, 0.13, 0.15, 0.15, 0.13, and 0.14 nm, respectively. Figure 3b showed that the ligand-binding induces conformational changes. It showed the major changes in the residues between 64–70, 90–95, 119–126, 140–154, and 202–216. We have observed a higher peak in the C-terminal region. From the RMSF analysis, we have seen that ligand binding altering the geometry of the protein structure and for performing the native function of any protein a proper conformation is required. The HsGSK3β-ZINC70704976 and HsGSK3β-ZINC70707119 showed less fluctuation as compared to other predicted ligands.

#### Radius of gyration

**Fig. 4:**
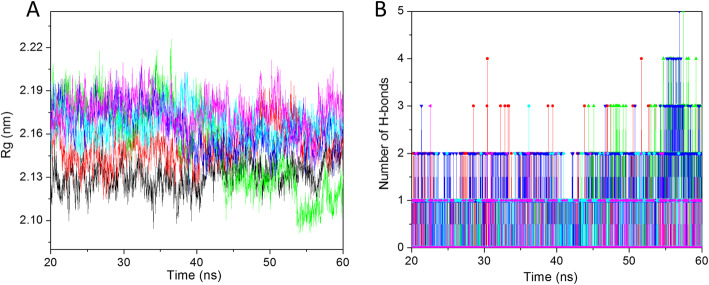
Rg and hydrogen bond. **a** The radius of gyration value for backbone at 300 K for last 40 ns trajectory. **b** Number of hydrogen bonds between protein and ligand for last 40 ns trajectory. The color code for all panels are apo-HsGSK3β (black), HsGSK3β-ANP (red), HsGSK3β-ZINC15968620 (green), HsGSK3β-ZINC15968622 (blue), HsGSK3β-ZINC70704976 (cyan), and HsGSK3β-ZINC70707119 (magenta)

The radius of gyration analysis of the protein-ligand complex is done by the radius of gyration (*Rg*) parameter analysis. The less Rg value is observed in the compactly folded protein while the higher Rg value is generally shown by the loosely folded protein structure. The Rg value for all the systems was calculated for the last 40 ns trajectory and plotted in Fig. 4a. The average Rg value were recorded 2.13, 2.15, 2.15, 2.16, 2.16, and 2.17 nm for apo-HsGSK3β, HsGSK3β-ANP, HsGSK3β-ZINC15968620, HsGSK3β-ZINC15968622, HsGSK3β-ZINC70704976, and HsGSK3β-ZINC70707119, respectively. The result showed that the least Rg value was observed for the apo-HsGSK3β as compared to other compounds and control compounds. It represents that after ligand binding, some conformational changes have occurred. The HsGSK3β-ZINC15968620 showed the same average Rg value as compared to the control compound. The HsGSK3β-ZINC15968622 and HsGSK3β-ZINC70704976 showed the same Rg value and higher than control while less than HsGSK3β-ZINC70704976. All the complexes showed the stable peak from 20 to 40 ns while the value for HsGSK3β-ZINC15968620 is decreasing from higher to lower with the timescale. From here, we have concluded that all predicted hits are good and showed the protein-ligand complex stability.

#### Interaction analysis

Various interactions between protein and ligand play a key role in protein-ligand stabilization. The hydrogen bonds are the key interaction between protein and ligand. They provide interaction specificity and directionality which is a key step in molecular recognition. We have calculated the number of hydrogen bonds *vs.* time and plotted it in Fig. 4b. All the complexes showed good bonding between protein and ligand. The average number of hydrogen bonds for HsGSK3β-ANP, HsGSK3β-ZINC15968620, HsGSK3β-ZINC15968622, HsGSK3β-ZINC70704976, and HsGSK3β-ZINC70707119 was 0–1 for all the ligand complexes. The HsGSK3β-ZINC15968620 and HsGSK3β-ZINC15968622 showed 0–3 hydrogen bonds respected time. The control compound also showed 3 hydrogen bonds in some time steps. It represents that these compounds stably interacted with the HsGSK3β binding cavity and provides stable interaction.

#### Solvent accessible surface area

**Fig. 5:**
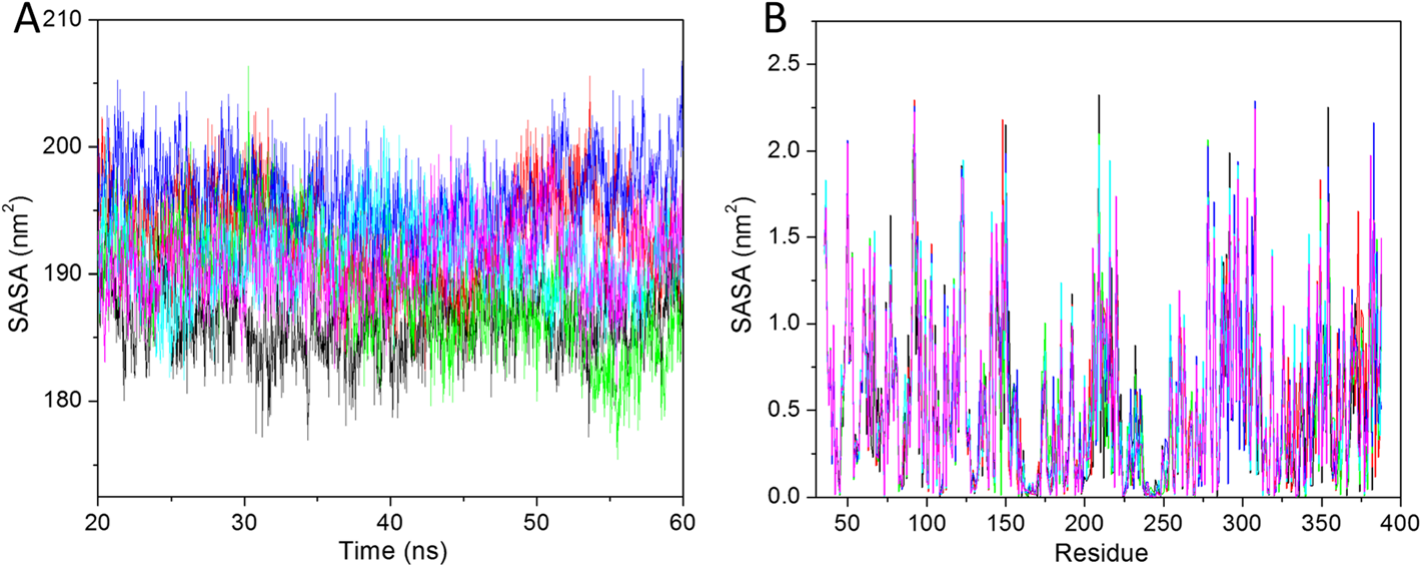
The solvent-accessible surface area as a function of time at 300 K. **a** SASA value vs*.* time. **b** SASA value for residues. The color code for all panels are apo-HsGSK3β (black), HsGSK3β-ANP (red), HsGSK3β-ZINC15968620 (green), HsGSK3β-ZINC15968622 (blue), HsGSK3β-ZINC70704976 (cyan), and HsGSK3β-ZINC70707119 (magenta)

The SASA of a protein defines the area which can be accessed by the solvent. The SASA value is often increased by hydrophobic residues. The solvation-free energy of protein is calculated by the polar and non-polar interaction. The SASA value is calculated by using the last 40 ns trajectory. The average SASA values were 186.23, 196.16, 188.84, 196.19, 191.18, and 190.93 nm^2^ calculated for apo-HsGSK3β, HsGSK3β-ANP, HsGSK3β-ZINC15968620, HsGSK3β-ZINC15968622, HsGSK3β-ZINC70704976, and HsGSK3β-ZINC70707119, respectively (Fig. 5a). Here also, we have seen results similar to Rg, where apo-GSK3β showed less value as compared to all other predicted hits. The control compounds HsGSK3β-ANP showed a very high SASA value as compared to all other predicted hits. It represents that our predicted ligands showed more complex stability as compared to the control ligand. The HsGSK3β-ZINC15968620 showed a high SASA value of 42 ns while after that it showed a stable and lower peak as compared to all other predicted compounds. The control compound showed lower and higher SASA values with respect to time while the HsGSK3β-ZINC70704976 and HsGSK3β-ZINC70707119 showed very stable peaks from starting (20 ns) till the 60 ns. The results showed that both complexes are very stable as compared to other predicted hits as well as the control compound.

We have also tracked the residue level changes by plotting the residue SASA value. It is an important parameter to understand the residual changes during ligand binding. The SASA value vs. residue plot is shown in Fig. 5b. The average SASA value for apo-HsGSK3β, HsGSK3β-ANP, HsGSK3β-ZINC15968620, HsGSK3β-ZINC15968622, HsGSK3β-ZINC70704976, and HsGSK3β-ZINC70707119 were 0.52, 0.54, 0.53, 0.55, 0.54, and 0.53 nm^2^, respectively. It represents that apo-GSK3β shows the least residual SASA as compared to all other predicted hits. In the case of predicted hits, the HsGSK3β-ZINC15968620 and HsGSK3β-ZINC70707119 showed 0.53 nm^2^ and it is the least value as compared to other predicted hits. It is confirmed from this finding that these compounds showed a stable complex.

#### Principal component analysis

**Fig. 6:**
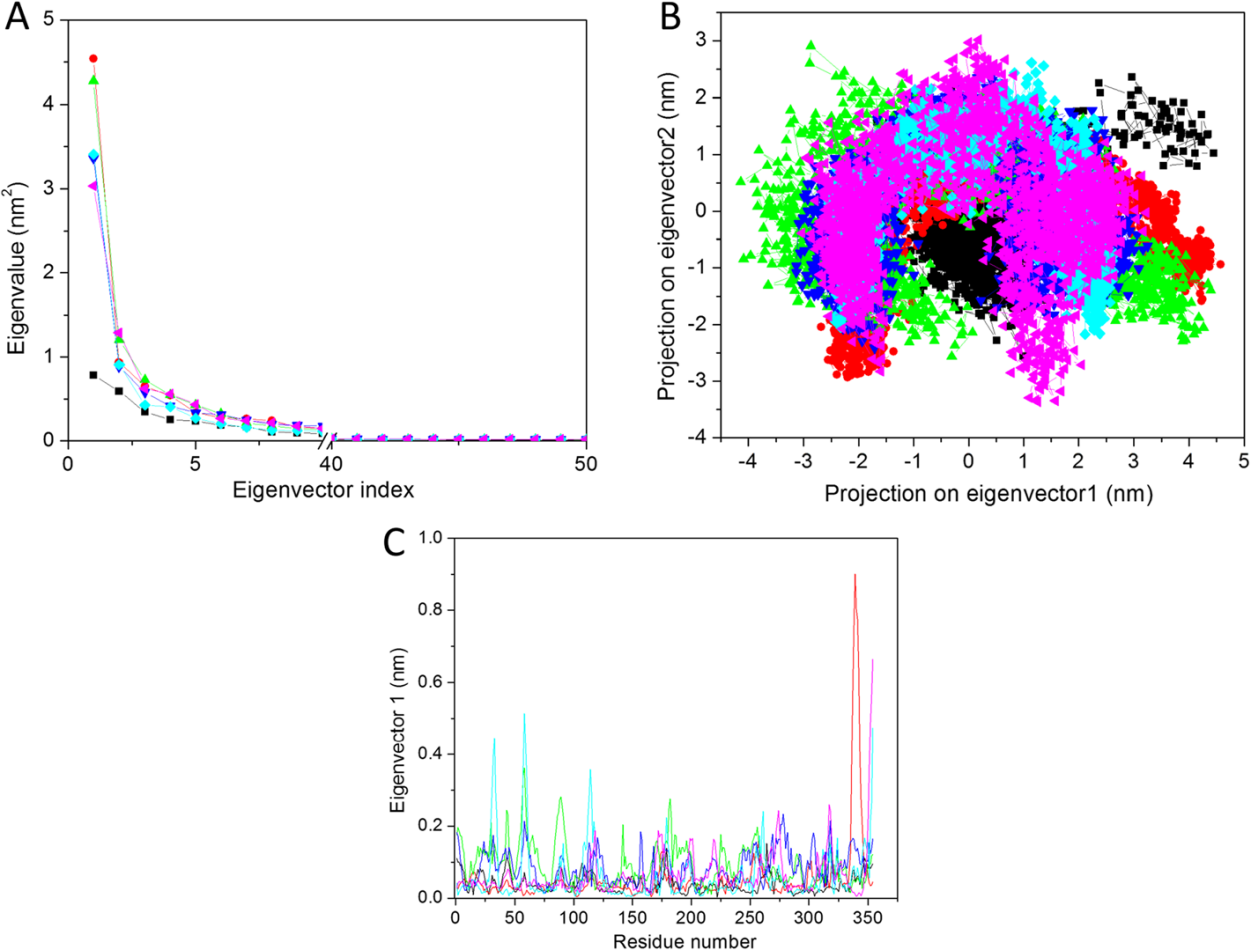
Principal component analysis. **a** The plot of eigenvalues vs*.* first 50 eigenvectors. **b** First two eigenvectors describing the projection of protein motion in phase space for all the systems. **c** eigRMSF obtained from the first PC during PCA calculations. The color code for all panels is apo-HsGSK3β (black), HsGSK3β-ANP (red), HsGSK3β-ZINC15968620 (green), HsGSK3β-ZINC15968622 (blue), HsGSK3β-ZINC70704976 (cyan), and HsGSK3β-ZINC70707119 (magenta)

The PCA or essential dynamics (ED) are used to analyze the correlated motions in the protein after ligand binding. The total motility in the system is equivalent to the sum of the eigenvalues. It can be used to compare the flexibility of a protein under different conditions. The Gromacs provide the facility to calculate the eigenvectors for characterizing the protein motions. Here we have considered the first fifty eigenvectors for result analysis because it is a well-known fact that the first few PCs describe the overall dynamics of the system. We have calculated the percent wise motions for the first five eigenvectors. The first five eigenvectors accounted for 52.49%, 70.70%, 72.35%, 65.83%, 69.84%, and 66.59% of the motions, recorded for last 40 ns trajectory for apo-HsGSK3β, HsGSK3β-ANP, HsGSK3β-ZINC15968620, HsGSK3β-ZINC15968622, HsGSK3β-ZINC70704976, and HsGSK3β-ZINC70707119, respectively (Fig. 6a). The apo-HsGSK3β showed very less motions as compared to ligand-bound complexes. It indicates that ligand binding induces structural changes and motions in the protein. In the case of the ligand-protein complex the HsGSK3β-ZINC15968622, HsGSK3β-ZINC70704976, and HsGSK3β-ZINC70707119 showed less motions as compared to the control compound. So from here, we have concluded that these three complexes can act as lead compounds.

In this result, we have observed that the first few eigenvectors were describing the overall dynamics of the protein. So in the next analysis, we have considered the first two eigenvectors, and a 2D projection plot was plotted to achieve the phase space behavior of the protein-ligand complex (Fig. 6b). In this plot, the clear cluster describes the well stable complex while the non-stable cluster defines the non-stable complex. In Fig. 6b, we have seen that apo-HsGSK3β showed a well stable cluster. In the case of the protein-ligand complex, the HsGSK3β-ZINC15968622, HsGSK3β-ZINC70704976, and HsGSK3β-ZINC70707119 showed well stable cluster as compared to the control compound. This result is also consistent with the above-stated analysis.

After that, we have calculated the eigRMSF for the one eigenvector vs*.* residues and plotted it in Fig. 6c. It describes the motions based on residues that were affected after ligand binding. We have calculated the eigRMSF value for all the protein-ligand complexes and shown in Fig. 6c. The average value for apo-HsGSK3β, HsGSK3β-ANP, HsGSK3β-ZINC15968620, HsGSK3β-ZINC15968622, HsGSK3β-ZINC70704976, and HsGSK3β-ZINC70707119 were 0.03, 0.05, 0.09, 0.08, 0.06, and 0.06 nm, respectively. The average value suggested that apo-protein showed very less motions as compare to all other protein-ligand complexes including the control compound. The HsGSK3β-ZINC70704976 and HsGSK3β-ZINC70707119 showed the least value as compared to other ligands but more than the control compound. The overall patterns were found similar to the RMSF analysis.

#### Gibbs free energy landscape

**Fig. 7:**
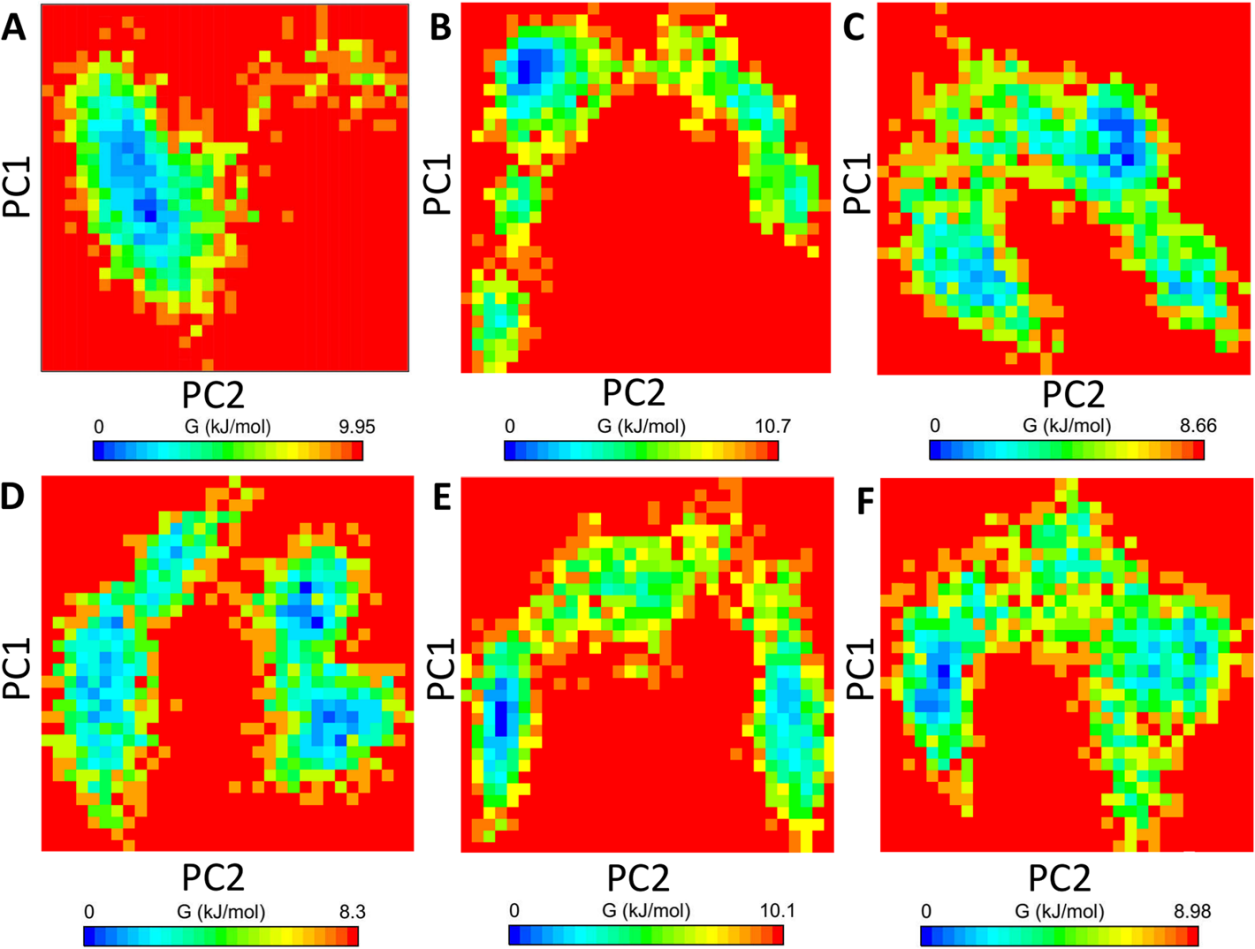
Gibbs free energy landscape obtained from first two PCs at 300 K. **a** HsGSK3β, **b** HsGSK3β-ANP, **c** HsGSK3β-ZINC15968620, **d** HsGSK3β-ZINC15968622, **e** HsGSK3β-ZINC70704976, and **f** HsGSK3β-ZINC70707119

The *gmx sham* tool was used for the calculation of the Gibbs free energy landscape. The projection of the first two principal components PC1 and PC2 was done for the prediction of Gibbs free energy landscape. The color-coded representation of the Gibbs free energy landscape for all the systems was shown in Fig. 7. The direction of fluctuation for all the *Cα* atoms was inspected for apo-HsGSK3β, HsGSK3β-ANP, HsGSK3β-ZINC15968620, HsGSK3β-ZINC15968622, HsGSK3β-ZINC70704976, and HsGSK3β-ZINC70707119 from the last 40 ns trajectory. The deeper blue color on the contour map represents the lower energy for all the systems. A higher blue color was observed for the control compound and HsGSK3β-ZINC15968620. It represents that these complexes have only one minimum state. The apo-HsGSK3β showed a very stable cluster with blue color, and it also represents only one stable conformation. In the case of HsGSK3β-ZINC15968622, HsGSK3β-ZINC70704976, and HsGSK3β-ZINC70707119, we have observed the two to four conformational states so it represents that these complexes have many energy minima. We have concluded from overall MD analysis that our all protein-ligand complexes showed robust stability.

#### Binding free energy analysis

For the investigation of the protein-ligand complex stability and binding of selected hits after MD simulation, binding free energy of the lead compounds was calculated by using Molecular-Mechanics Poisson Boltzmann Surface Area (MM-PBSA) method. We have selected the last five ns trajectory for the calculation of binding free energy. It calculated the polar and non-polar solvation energy in energetic terms like electrostatic interaction, Van der Walls energy, and SASA energy. The average binding affinity for all the protein-ligand complexes was summarized in Table 2. The binding affinity for HsGSK3β-ANP, HsGSK3β-ZINC15968620, HsGSK3β-ZINC15968622, HsGSK3β-ZINC70704976, and HsGSK3β-ZINC70707119 were −150.49, −159.86, −161.42, −143.55, and −159.54 kJ.mol^-1^, respectively. All the compounds showed greater binding affinity than control compound ANP except HsGSK3β-ZINC70704976 while it showed higher Van der Walls energy, electrostatic energy, and polar solvation energy than the control compound. It represents that this compound also acts as an inhibitor against HsGSK3β. The HsGSK3β-ZINC15968622 showed the highest binding affinity than all other selected hits. From here, we have concluded that these complexes are energetically favorable and can act as a novel compound against HsGSK3β.

**Table 2:** The table represents the Van der Waals, electrostatic, polar solvation, SASA, and binding energy in kJ.mol^-1^ for control compound and predicted hits

S. No.	Compound	Van der Waals energy	Electrostatic energy	Polar solvation energy	SASA energy	Binding energy
1.	ANP	-234.83 ± 16.12	-11.20 ± 7.04	115.37 ± 18.46	-19.82 ± 1.11	-150.49 ± 22.57
2.	ZINC15968620	-233.65 ± 15.30	-16.37 ± 7.83	112.65 ± 22.21	-22.50 ± 1.46	-159.86 ± 20.28
3.	ZINC15968622	-238.74 ± 16.94	-14.22 ± 7.28	114.71 ± 20.41	-23.18 ± 1.53	-161.42 ± 15.76
4.	ZINC70704976	-267.56 ± 14.38	-30.88 ± 10.22	179.11 ± 26.14	-24.22 ± 1.15	-143.55 ± 20.84
5.	ZINC70707119	-247.80 ± 12.58	-19.63 ± 6.95	129.82 ± 24.71	-21.94 ± 1.04	-159.54 ± 23.47

**Fig. 8:**
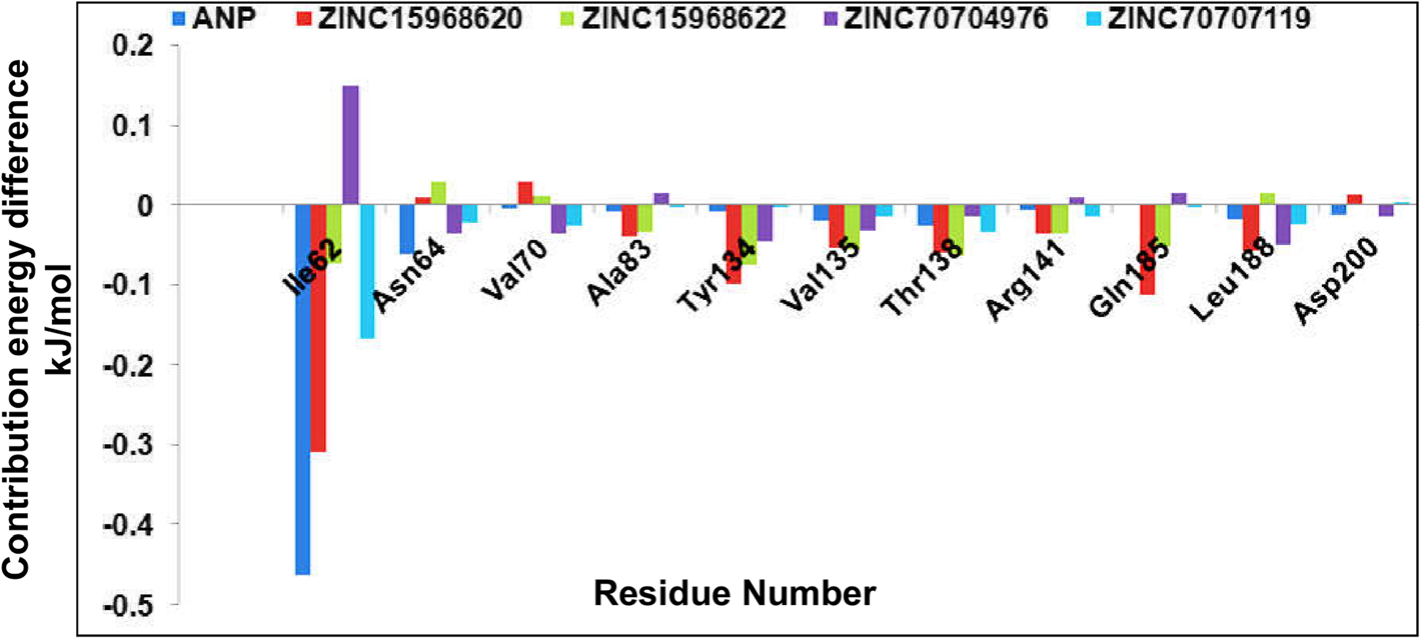
Residual binding free energy decomposition. The plot describes the contribution of each amino acid to the binding of ligands

In structure-based drug designing, the residual contribution in binding is very important. We have identified the binding affinity based on each residue. For a clear depiction of the result, we have taken the catalytically important residues only, which play a key role in ligand binding, and plotted in Fig 8. In the figure, we have seen that Ile62, Asn62, Val70, Ala83, Tyr134, Val135, Thr138, Arg141, and Gln185 play a key role in ligand binding. Looking into these residues, it was observed that most of these are hydrophobic; therefore, from here we have concluded that hydrophobic interaction plays a key role in HsGSK3β and ligand stabilization. We have also found a few key residues which play important role in ligand binding.

## Discussion

AD is a complex and multifactorial disease. It is mainly characterized by memory loss and generally affects old age people. The Aβ and NFTs are the main key hallmarks for disease identification. The amyloid precursor protein and tau protein form the Aβ plaques and NFTs, respectively. Several enzymes play an important role in the disease progression in which the HsGSK3β plays a key role in the phosphorylation of the tau protein and due to the hyperphosphorylation tau protein forms the NFTs [48]. These NFTs promote neuron death and actively participate in AD progression. Hence, inhibition of the HsGSK3β reduces the tau phosphorylation in various studies [18]. We have taken a subset of natural compounds and screened against the HsGSK3β in four sequential docking runs. From the screening, we have selected the top 404 compounds for the ADMET analysis, which is a required parameter to produce the compounds in the market [49]. Then, the ADMET value is predicted by using the admetSAR server [35]. The admetSAR server can predict various physiochemical properties such as BBB, HIA, Caco-2, Pgp substrate-inhibitor, Cyp450 substrate/inhibitor, lethal dose, toxicity, and carcinogenicity. We have predicted all these parameters, and on the basis of all these parameters, we have selected 20 compounds for the redocking analysis by using MVD, Autodock Vina, and Autodock. These compounds were redocked by using the MVD, Autodock, and Autodock Vina [36] software with the ANP. The redocking suggested four best compounds (ZINC15968620, ZINC15968622, ZINC70704976, and ZINC70707119) which are used for further MDS analysis. The MDS is a widely used method to predict the protein-ligand stability of the docked complex. Hence, we have also performed 100 ns MD simulation for all the complexes. Several structural parameters such as RMSD, RMSF, Rg, SASA, PCA, and binding free energy analysis were carried out. The RMSD value suggested that all the complexes are stable and producing the equilibrated trajectory for further analysis. Hence, we have calculated various other structural parameters such as RMSF, Rg, PCA, Gibbs free energy, and binding free energy analysis. The hydrogen bonds analysis suggest that all the complexes are stable and showing the interaction with the key catalytic residues of the binding site. The essential dynamics analysis also agreed with this result. Then we have also carried out the Gibbs free energy landscape analysis. The analysis suggested that some complexes followed the stable state with the metastable state. The binding free energy analysis showed that out of four compounds three compounds (ZINC15968620, ZINC15968622, and ZINC70707119) are better than ZNC70704976. Hence from all these results we have selected ZINC15968620, ZINC15968622, and ZINC70707119 that can act as a lead compound against the HsGSK3β to reduce the NFTs burden from the cell. We have proposed these compounds to the global scientific community as they can further evaluate these compounds by using the in vitro and in vivo techniques.

## Conclusion

AD is a major problem for our society at a global level, and there is a need to look for possible treatment strategies. The HsGSK3β is an important protein kinase involved in tau hyperphosphorylation in AD including several other diseases. So in this study, we have targeted the HsGSK3β to reduce the hyperphosphorylation of tau protein. We have used various computational approaches for predicting the small molecules against GSK3β which can bind to the active site and can block the enzyme activity. We have retrieved the natural compound library (*n*=167,741) from the ZINC database and screened it against HsGSK3β in various steps by using MVD. From the virtual screening, we have selected 404 compounds. These compounds were further employed for the pharmacokinetic analysis. From this analysis, 20 compounds were selected and employed for the redocking. Then selected 4 plausible compounds from docking that were employed for MDS. From MDS, finally, three compounds ZINC15968620, ZINC15968622, and ZINC70707119 were selected and proposed as potent lead compounds against HsGSK3β. These compounds can be further evaluated through in vitro and in vivo experiments. These compounds will serve as an initial point to design the novel inhibitors against HsGSK3β and can act as a novel therapeutic compound for AD. It is anticipated that proposed compounds will provide ready to use input for the experimental scientists and after their respective verifications could help in the management of tauopathies and AD.

## Supplementary Information

**Additional file 1 **Figure 1. The figure represents the common ring in all the four compounds. The red circle represents the different group in the compounds.s. **Supplementary Table S1.** *In-silico* absorption and distribution profile obtained from admetSAR server for selected 404 compounds. Selected compounds (20) for redocking were highlighted in bold. **Supplementary Table S2.** *In-silico* Cyp450 enzyme metabolism profile was obtained from admetSAR server for selected 404 compounds. Selected compounds (20) for redocking were highlighted in bold. **Supplementary Table S3.** *In-silico* toxicity, carcinogenicity and LD_50_ profile obtained from admetSAR server for selected 404 compounds. Selected compounds (20) for redocking were highlighted in bold. **Supplementary Table S4.** Summary of binding affinity with interacting residues of the top 20 compounds with control compound ANP obtained from molecular docking studies by three docking tools: Autodock Tools, AutodockVina and Molegro Virtual Docker. The Residues which involved in hydrogen bonding were highlighted in bold as well as selected hits for MDS are also highlighted in bold.

## Data Availability

Provided in the form of the supplementary table.

## Abbreviations

AD: Alzheimer’s disease
NFTs: Neurofibrillary tangles
GSK-3*β*: Glycogen synthase kinase 3 beta
MDS: Molecular dynamics simulation
PCA: Principal component analysis
MM-PBSA: Molecular Machenics – Poisson Boltzmann Surface Area
