# Supplementary material for: High-throughput screening of natural compounds and inhibition of a major therapeutic target HsGSK-3β for Alzheimer’s disease using computational approaches
Source: J Genet Eng Biotechnol. 2021 May 4;19:61. doi: 10.1186/s43141-021-00163-w (PMC8096881; doi:10.1186/s43141-021-00163-w)
Supplement: Supplementary file 1 — Additional file 1 Figure 1. The figure represents the common ring in all the four compounds. The red circle represents the different group in the compounds.s. Supplementary Table S1. In-silico absorption and distribution profile obtained from admetSAR server for selected 404 compounds. Selected compounds (20) for redocking were highlighted in bold. Supplementary Table S2. In-silico Cyp450 enzyme metabolism profile was obtained from admetSAR server for selected 404 compounds. Selected compounds (20) for redocking were highlighted in bold. Supplementary Table S3. In-silico toxicity, carcinogenicity and LD50 profile obtained from admetSAR server for selected 404 compounds. Selected compounds (20) for redocking were highlighted in bold. Supplementary Table S4. Summary of binding affinity with interacting residues of the top 20 compounds with control compound ANP obtained from molecular docking studies by three docking tools: Autodock Tools, AutodockVina and Molegro Virtual Docker. The Residues which involved in hydrogen bonding were highlighted in bold as well as selected hits for MDS are also highlighted in bold. [file 43141_2021_163_MOESM1_ESM.docx]

**Supplementary Material**

**High-throughput screening of natural compounds and inhibition of a major therapeutic target HsGSK-3*β* for Alzheimer’s disease using Computational approaches**

**Running title**

*Identification of novel natural compounds against HsGSK3β enzyme*

**Figure 1**. The figure represents the common ring in all the four compounds. The red circle represents the different group in the compounds.

**Supplementary Table S1.** *In-silico* absorption and distribution profile obtained from admetSAR server for selected 404 compounds. Selected compounds (20) for redocking were highlighted in bold.

| **Sr. No.** | **ZINC ID** | **BBB probability** | **HIA-probability** | **Caco-2 permeability probability** | **Caco-2 permeability** | **P-gp substrate** | **P-gp inhibitor** |
| --- | --- | --- | --- | --- | --- | --- | --- |
|  | ZINC01530886 | +/0.9388 | +/0.9969 | -/0.8142 | 1.2144 | Substrate | Inhibitor |
|  | ZINC02096969 | -/0.6478 | +/9087 | -/0.6462 | 0.2135 | Substrate | Non-Inhibitor |
|  | ZINC02097182 | +/0.6020 | +/0.9577 | -/0.6742 | 0.1994 | Substrate | Non-Inhibitor |
|  | ZINC02118796 | +/0.6965 | +/0.9529 | -/0.6532 | 0.2165 | Substrate | Non-Inhibitor |
|  | ZINC02121154 | +/0.6394 | +/0.9533 | -/0.6468 | 0.2623 | Substrate | Non-Inhibitor |
|  | ZINC02125476 | +/0.7362 | +/0.9641 | -/0.6470 | 0.1913 | Substrate | Non-Inhibitor |
|  | ZINC02128340 | +/0.8772 | +/1.0000 | -/0.6283 | 1.1043 | Substrate | Non-Inhibitor |
|  | ZINC02128421 | -/0.5319 | +/0.8467 | -/0.6895 | 0.0453 | Substrate | Non-Inhibitor |
|  | ZINC02128423 | -/0.5319 | +/0.8467 | -/0.6895 | 0.0453 | Substrate | Non-Inhibitor |
|  | ZINC02128602 | +/0.5805 | +/0.9969 | -/0.6921 | 0.7023 | Substrate | Inhibitor |
|  | ZINC02129853 | +/0.6436 | +/0.9494 | -/0.6474 | 0.2236 | Substrate | Non-Inhibitor |
|  | ZINC02129857 | -/0.7620 | +/0.5617 | -/0.7246 | 0.2340 | Substrate | Non-Inhibitor |
|  | ZINC02130074 | +/0.8013 | +/1.0000 | -/0.6479 | 1.0202 | Substrate | Inhibitor |
|  | ZINC02130079 | +/0.8667 | +/1.0000 | -/0.6727 | 0.9166 | Substrate | Non-Inhibitor |
|  | ZINC02130200 | +/0.5401 | +/1.0000 | -/0.6196 | 1.0513 | Substrate | Non-Inhibitor |
|  | ZINC02130322 | -/0.6442 | +/0.8607 | -/0.7172 | 0.0260 | Substrate | Non-Inhibitor |
|  | ZINC02130539 | +/0.6955 | +/0.9956 | -/0.6457 | 1.0697 | Substrate | Inhibitor |
|  | ZINC02130817 | +/0.6816 | +/0.9969 | -/0.6535 | 1.1154 | Substrate | Inhibitor |
|  | ZINC02130826 | +/0.7875 | +/1.0000 | -/0.7058 | 0.7096 | Substrate | Non-Inhibitor |
|  | ZINC02131176 | -/0.5139 | -/0.6996 | -/0.7877 | -0.0081 | Substrate | Non-Inhibitor |
|  | ZINC02131179 | -/0.5139 | -/0.6996 | -/0.7877 | -0.0081 | Substrate | Non-Inhibitor |
|  | ZINC02131227 | -/0.7620 | +/0.5617 | -/0.7246 | 0.2340 | Substrate | Non-Inhibitor |
|  | ZINC02131415 | +/0.6622 | +/0.9969 | -/0.5996 | 0.7997 | Non-Substrate | Non-Inhibitor |
|  | ZINC02131893 | -/0.5748 | +/0.9963 | -/0.6900 | 1.0728 | Substrate | Non-Inhibitor |
|  | ZINC02131897 | -/0.5748 | +/0.9963 | -/0.6900 | 1.0728 | Substrate | Non-Inhibitor |
|  | ZINC02133098 | +/0.5352 | -/0.6347 | -/0.7839 | -0.0320 | Substrate | Non-Inhibitor |
|  | ZINC02133383 | -/0.6288 | +/0.9932 | -/0.6480 | 0.9432 | Substrate | Inhibitor |
|  | ZINC02133431 | +/0.7750 | +/1.0000 | -/0.5989 | 1.1133 | Substrate | Non-Inhibitor |
|  | ZINC02133462 | -/0.6302 | +/0.9963 | -/0.6580 | 0.9397 | Substrate | Non-Inhibitor |
|  | ZINC02133485 | +/0.5196 | +/0.8801 | -/0.6851 | 0.0263 | Substrate | Non-Inhibitor |
|  | ZINC02133487 | +/0.5196 | +/0.8801 | -/0.6851 | 0.0263 | Substrate | Non-Inhibitor |
|  | ZINC02134726 | +/0.8546 | +/1.0000 | -/0.6445 | 1.1965 | Substrate | Inhibitor |
|  | ZINC02134956 | +/0.7875 | +/1.0000 | -/0.7058 | 0.7096 | Substrate | Non-Inhibitor |
|  | ZINC02135285 | -/0.5461 | -/0.5604 | -/0.7212 | 0.0683 | Substrate | Non-Inhibitor |
|  | ZINC02135300 | -/0.6964 | +/0.8227 | -/0.7254 | 0.0404 | Substrate | Non-Inhibitor |
|  | ZINC02135304 | -/0.6964 | +/0.8227 | -/0.7254 | 0.0404 | Substrate | Non-Inhibitor |
|  | ZINC02135455 | -/0.6561 | +/0.5540 | -/0.7312 | 0.1413 | Substrate | Non-Inhibitor |
|  | ZINC02135875 | +/0.8092 | +/0.9962 | -/0.6533 | 0.9732 | Substrate | Inhibitor |
|  | ZINC02135983 | +/0.6635 | +/1.0000 | -/0.6490 | 0.8940 | Substrate | Inhibitor |
|  | ZINC02137586 | -/0.5447 | +/0.8809 | -/0.6788 | 0.0697 | Substrate | Non-Inhibitor |
|  | ZINC02137697 | +/0.5939 | +/1.0000 | -/0.6124 | 1.0964 | Substrate | Non-Inhibitor |
|  | ZINC02137876 | +/0.5253 | +/0.9955 | -/0.6258 | 1.0400 | Substrate | Non-Inhibitor |
|  | ZINC02145637 | +/0.8883 | +/0.9965 | +/0.6858 | 1.0936 | Substrate | Inhibitor |
|  | ZINC02146033 | +/0.9466 | +/1.0000 | +/0.7102 | 1.3092 | Substrate | Inhibitor |
|  | ZINC02146060 | +/0.9384 | +/1.0000 | +/0.6896 | 1.3587 | Substrate | Inhibitor |
|  | ZINC02146088 | +/0.9381 | +/0.9967 | +/0.7534 | 1.3625 | Substrate | Inhibitor |
|  | ZINC02160816 | -/0.6057 | +/0.9948 | -/0.6502 | 0.8706 | Substrate | Inhibitor |
|  | ZINC02160958 | +/0.6601 | +/1.0000 | -/0.6481 | 0.9829 | Substrate | Inhibitor |
|  | ZINC02161189 | -/0.7695 | -/0.6293 | -/0.7381 | 0.3073 | Substrate | Non-Inhibitor |
|  | ZINC02161190 | -/0.7695 | -/0.6293 | -/0.7381 | 0.3073 | Substrate | Non-Inhibitor |
|  | ZINC02161303 | +/0.6816 | +/0.9969 | -/0.6535 | 1.1154 | Substrate | Inhibitor |
|  | ZINC03983911 | +/0.7005 | -/0.9403 | -/0.8249 | 0.3850 | Substrate | Non-Inhibitor |
|  | ZINC04204381 | +/0.5368 | +/0.9448 | +/0.5000 | 0.8671 | Non-Substrate | Inhibitor |
|  | ZINC04268355 | +/0.7016 | +/0.8782 | -/0.5318 | 0.4563 | Substrate | Non-Inhibitor |
|  | ZINC04273402 | +/0.7226 | +/0.7619 | +/0.5206 | 0.6563 | Substrate | Non-Inhibitor |
|  | ZINC04281017 | +/0.6598 | +/0.9892 | +/0.6065 | 1.1906 | Non-Substrate | Inhibitor |
|  | ZINC04292491 | +/0.7737 | +/1.0000 | -/0.5554 | 1.1672 | Non-Substrate | Inhibitor |
|  | ZINC04292705 | +/0.8045 | +/0.9962 | -/0.5600 | 1.0681 | Non-Substrate | Inhibitor |
|  | ZINC04293318 | +/0.8580 | +/0.9974 | -/0.5382 | 1.1444 | Non-Substrate | Inhibitor |
|  | ZINC04293322 | +/0.8580 | +/0.9974 | -/0.5382 | 1.1444 | Non-Substrate | Inhibitor |
|  | ZINC04293326 | +/0.8336 | +/0.9952 | -/0.5262 | 1.1646 | Non-Substrate | Inhibitor |
|  | ZINC04293328 | +/0.8860 | +/1.0000 | +/0.5292 | 1.1838 | Non-Substrate | Inhibitor |
|  | ZINC04293329 | +/0.9174 | +/1.0000 | +/0.5320 | 1.3712 | Non-Substrate | Inhibitor |
|  | ZINC04293330 | +/0.7085 | +/0.9949 | -/0.5175 | 1.0281 | Non-Substrate | Inhibitor |
|  | ZINC04293377 | +/0.6094 | +/0.8637 | -/0.6402 | 0.6150 | Non-Substrate | Inhibitor |
|  | ZINC04293484 | +/0.9637 | +/0.9569 | +/0.5000 | 1.4899 | Non-Substrate | Non-Inhibitor |
|  | ZINC04293487 | +/0.7617 | +/0.9965 | -/0.5171 | 0.9046 | Non-Substrate | Non-Inhibitor |
|  | ZINC04293736 | +/0.9348 | +/1.0000 | -/0.5345 | 1.1342 | Non-Substrate | Inhibitor |
|  | ZINC04691948 | +/0.5702 | -/0.9716 | -/0.6602 | 0.3657 | Substrate | Non-Inhibitor |
|  | ZINC04712260 | +/0.7737 | +/1.0000 | -/0.5554 | 1.1672 | Non-Substrate | Inhibitor |
|  | ZINC06500907 | +/0.8726 | +/0.9922 | +/0.5383 | 1.1908 | Substrate | Inhibitor |
|  | ZINC06500915 | +/0.8726 | +/0.9922 | +/0.5383 | 1.1908 | Substrate | Inhibitor |
|  | ZINC06631508 | +/0.8500 | +/0.9932 | +/0.5426 | 1.2001 | Substrate | Inhibitor |
|  | ZINC08214433 | +/0.8739 | +/0.9963 | +/0.5210 | 1.3387 | Non-Substrate | Non-Inhibitor |
|  | ZINC08382321 | +/0.9172 | +/0.8741 | -/0.6151 | 0.1504 | Non-Substrate | Non-Inhibitor |
|  | ZINC08382323 | +/0.9172 | +/0.8741 | -/0.6151 | 0.1504 | Non-Substrate | Non-Inhibitor |
|  | ZINC08382324 | +/0.9172 | +/0.8741 | -/0.6151 | 0.1504 | Non-Substrate | Non-Inhibitor |
|  | ZINC08398296 | +/0.9677 | +/1.0000 | -/0.6197 | 1.1151 | Non-Substrate | Non-Inhibitor |
|  | ZINC08398409 | +/0.5370 | +/0.6487 | -/0.6263 | 0.5369 | Substrate | Inhibitor |
|  | ZINC08790054 | +/0.9470 | +/0.9930 | -/0.6813 | 0.8237 | Non-Substrate | Non-Inhibitor |
|  | ZINC08790412 | +/0.7777 | +/0.9347 | -/0.6841 | 0.6557 | Non-Substrate | Non-Inhibitor |
|  | ZINC08790736 | +/0.6449 | +/1.0000 | -/0.6630 | 1.1625 | Substrate | Inhibitor |
|  | ZINC08790787 | +/0.5465 | +/0.9966 | -/0.6379 | 0.9565 | Substrate | Non-Inhibitor |
|  | ZINC08790849 | +/0.5465 | +/0.9966 | -/0.6379 | 0.9565 | Substrate | Non-Inhibitor |
|  | ZINC08790961 | +/0.8146 | +/0.9974 | -/0.7075 | 0.8403 | Substrate | Non-Inhibitor |
|  | ZINC08791059 | +/0.9005 | +/1.0000 | -/0.6108 | 1.2087 | Substrate | Non-Inhibitor |
|  | ZINC08791123 | +/0.7750 | +/1.0000 | -/0.5989 | 1.1133 | Substrate | Non-Inhibitor |
|  | ZINC08791133 | +/0.5547 | +/1.0000 | -/0.6124 | 1.0948 | Substrate | Inhibitor |
|  | ZINC08791324 | +/0.8605 | +/0.9966 | -/0.6324 | 1.0936 | Substrate | Inhibitor |
|  | ZINC08917941 | +/0.7809 | +/1.0000 | +/0.6979 | 0.9647 | Non-Substrate | Inhibitor |
|  | ZINC08918002 | +/0.8173 | +/1.0000 | +/0.6483 | 0.8182 | Non-Substrate | Inhibitor |
|  | ZINC08918038 | -/0.5705 | +/0.9971 | -/0.6465 | 1.0441 | Substrate | Inhibitor |
|  | ZINC08918050 | -/0.6721 | +/0.9836 | -/0.6218 | 0.9636 | Substrate | Inhibitor |
|  | ZINC08918440 | +/0.6327 | +/0.9838 | -/0.6344 | 0.8499 | Substrate | Inhibitor |
|  | ZINC09312660 | +/0.7294 | +/0.9595 | -/0.5124 | 0.9243 | Substrate | Inhibitor |
|  | ZINC09373722 | +/0.7294 | +/0.9595 | -/0.5124 | 0.9243 | Substrate | Inhibitor |
|  | ZINC11867664 | +/0.8681 | +/0.9970 | +/0.5812 | 0.9834 | Non-Substrate | Inhibitor |
|  | ZINC11868779 | +/0.8836 | +/1.0000 | +/0.5948 | 0.8403 | Substrate | Inhibitor |
|  | ZINC11868805 | +/0.6975 | +/1.0000 | +/0.6872 | 1.0131 | Substrate | Inhibitor |
|  | ZINC11868862 | +/0.8662 | +/1.0000 | +/0.6839 | 0.9237 | Non-Substrate | Inhibitor |
|  | ZINC11868946 | +/0.7948 | +/0.9958 | +/0.7377 | 1.1925 | Substrate | Inhibitor |
|  | ZINC11869394 | +/0.8341 | +/1.0000 | +/0.6050 | 0.8550 | Substrate | Inhibitor |
|  | ZINC11869400 | +/0.8571 | +/1.0000 | +/0.5611 | 0.7230 | Substrate | Inhibitor |
|  | ZINC11869425 | +/0.9409 | +/0.9960 | +/0.5872 | 0.7373 | Substrate | Inhibitor |
|  | ZINC12662395 | +/0.7809 | +/1.0000 | +/0.6979 | 0.9647 | Non-Substrate | Inhibitor |
|  | ZINC12872711 | -/0.5437 | +/1.0000 | -/0.6527 | 0.8842 | Substrate | Inhibitor |
|  | ZINC12880349 | +/0.5816 | +/1.0000 | -/0.6542 | 1.0151 | Substrate | Inhibitor |
|  | ZINC12880848 | +/0.8423 | +/0.8971 | -/0.5643 | 0.5890 | Non-Substrate | Non-Inhibitor |
|  | ZINC12882432 | +/0.8126 | +/1.0000 | -/0.5755 | 1.1442 | Substrate | Non-Inhibitor |
|  | ZINC12882846 | -/0.5437 | +/1.0000 | -/0.6527 | 0.8842 | Substrate | Inhibitor |
|  | ZINC12883224 | +/0.7152 | +/1.0000 | -/0.6577 | 0.8791 | Substrate | Non-Inhibitor |
|  | ZINC12883239 | +/0.8421 | +/1.0000 | -/0.6743 | 0.9490 | Substrate | Non-Inhibitor |
|  | ZINC12883509 | +/0.5761 | +/0.9936 | -/0.6451 | 0.6955 | Substrate | Non-Inhibitor |
|  | ZINC15953437 | -/0.5753 | +/0.9939 | -/0.5442 | 0.9332 | Non-Substrate | Inhibitor |
|  | **ZINC15968620** | +/0.6133 | +/0.9092 | -/0.6069 | 0.8325 | Non-Substrate | Non-Inhibitor |
|  | **ZINC15968622** | +/0.6133 | +/0.9092 | -/0.6069 | 0.8325 | Non-Substrate | Non-Inhibitor |
|  | ZINC18007499 | +/0.5313 | -/0.7984 | -/0.8472 | 0.2510 | Substrate | Non-Inhibitor |
|  | ZINC18158134 | +/0.8467 | +/0.9764 | -/0.5668 | 1.0068 | Non-Substrate | Inhibitor |
|  | ZINC18163300 | -/0.5728 | -/0.9459 | -/0.6796 | 0.2655 | Substrate | Non-Inhibitor |
|  | ZINC19721276 | -/0.5728 | -/0.9459 | -/0.6796 | 0.2655 | Substrate | Non-Inhibitor |
|  | ZINC19866195 | +/0.6676 | -/0.8442 | -/0.6804 | 0.3097 | Substrate | Non-Inhibitor |
|  | ZINC22443609 | +/0.6780 | +/0.5797 | -/0.5493 | 0.3436 | Substrate | Non-Inhibitor |
|  | ZINC26671872 | +/0.6842 | -/0.9299 | -/0.6870 | 0.3576 | Substrate | Non-Inhibitor |
|  | ZINC28539034 | +/0.6095 | -/0.6799 | -/0.6654 | 0.3309 | Substrate | Non-Inhibitor |
|  | ZINC30724344 | -/0.5179 | -/0.7739 | -/0.6462 | 0.4667 | Substrate | Non-Inhibitor |
|  | ZINC30725806 | -/0.5806 | -/0.7723 | -/0.8206 | 0.1471 | Substrate | Non-Inhibitor |
|  | ZINC30725812 | -/0.5806 | -/0.7723 | -/0.8206 | 0.1471 | Substrate | Non-Inhibitor |
|  | ZINC32786262 | +/0.5334 | -/0.9394 | -/0.7148 | 0.1581 | Substrate | Non-Inhibitor |
|  | ZINC32789745 | -/0.5806 | -/0.7723 | -/0.8206 | 0.1471 | Substrate | Non-Inhibitor |
|  | ZINC38139950 | +/0.6117 | +/0.9969 | -/0.6573 | 0.7798 | Substrate | Non-Inhibitor |
|  | ZINC38139967 | +/0.5899 | +/0.9619 | -/0.6158 | 1.0580 | Substrate | Non-Inhibitor |
|  | ZINC38139969 | +/0.8002 | +/0.9278 | -/0.5574 | 1.1535 | Substrate | Non-Inhibitor |
|  | ZINC38139983 | +/0.8002 | +/0.9278 | -/0.5574 | 1.1535 | Substrate | Non-Inhibitor |
|  | ZINC38140001 | +/0.6457 | +/0.9957 | -/0.5844 | 1.0328 | Substrate | Non-Inhibitor |
|  | ZINC38140007 | +/0.6969 | +/0.9968 | -/0.6837 | 0.6844 | Substrate | Non-Inhibitor |
|  | ZINC38140019 | +/0.6969 | +/0.9968 | -/0.6837 | 0.6844 | Substrate | Non-Inhibitor |
|  | ZINC38140043 | +/0.6457 | +/0.9957 | -/0.5844 | 1.0328 | Substrate | Non-Inhibitor |
|  | ZINC38140045 | +/0.5855 | +/1.0000 | -/0.6566 | 0.8902 | Substrate | Non-Inhibitor |
|  | ZINC38140047 | +/0.5855 | +/1.0000 | -/0.6566 | 0.8902 | Substrate | Non-Inhibitor |
|  | ZINC44459964 | -/0.5822 | +/1.0000 | -/0.5461 | 1.0495 | Substrate | Non-Inhibitor |
|  | ZINC53276076 | +/0.7005 | -/0.9403 | -/0.8249 | 0.3850 | Substrate | Non-Inhibitor |
|  | ZINC53682947 | +/0.6603 | -/0.9038 | -/0.6315 | 0.4075 | Substrate | Inhibitor |
|  | ZINC56871207 | -/0.5806 | -/0.7723 | -/0.8206 | 0.1471 | Substrate | Non-Inhibitor |
|  | **ZINC65731330** | +/0.8180 | -/0.8772 | -/0.7416 | -0.4171 | Non-Substrate | Non-Inhibitor |
|  | ZINC67903538 | -/0.5921 | -/0.7931 | -/0.7931 | -0.6055 | Non-Substrate | Non-Inhibitor |
|  | ZINC67913695 | +/0.7558 | +/0.6037 | -/0.8294 | -/0.7657 | Substrate | Inhibitor |
|  | ZINC68568464 | +/0.9004 | +/0.9454 | -/0.5698 | 0.8268 | Substrate | Non-Inhibitor |
|  | ZINC68581659 | +/0.8783 | +/0.9954 | +/0.5455 | 1.0999 | Non-Substrate | Inhibitor |
|  | ZINC68581663 | +/0.8783 | +/0.9954 | +/0.5455 | 1.0999 | Non-Substrate | Inhibitor |
|  | ZINC68581666 | +/0.8783 | +/0.9954 | +/0.5455 | 1.0999 | Non-Substrate | Inhibitor |
|  | ZINC68603562 | +/0.8139 | +/0.5749 | -/0.5270 | 0.7652 | Substrate | Non-Inhibitor |
|  | ZINC68604313 | +/0.6215 | -/0.9938 | -/0.6566 | -0.6360 | Substrate | Non-Inhibitor |
|  | ZINC70670071 | -/0.5127 | +/0.6007 | -/0.5726 | 0.0338 | Substrate | Inhibitor |
|  | ZINC70673869 | +/0.9403 | +/1.0000 | +/0.7085 | 1.2678 | Substrate | Inhibitor |
|  | ZINC70686632 | +/0.7591 | +/1.0000 | +/0.7338 | 1.1162 | Non-Substrate | Inhibitor |
|  | ZINC70686670 | +/0.6975 | +/1.0000 | +/0.6872 | 1.0131 | Substrate | Inhibitor |
|  | ZINC70686752 | +/0.7809 | +/1.0000 | +/0.6979 | 0.9647 | Non-Substrate | Inhibitor |
|  | ZINC70687241 | -/0.5299 | +/0.9919 | -/0.6367 | 0.9557 | Substrate | Inhibitor |
|  | ZINC70687549 | +/0.5832 | +/0.9947 | -/0.6155 | 0.7624 | Substrate | Inhibitor |
|  | ZINC70687967 | +/0.6327 | +/0.9838 | -/0.6344 | 0.8499 | Substrate | Inhibitor |
|  | ZINC70691607 | +/0.9318 | +/0.9575 | +/0.5777 | 0.9337 | Substrate | Inhibitor |
|  | ZINC70692032 | +/0.9067 | +/0.8737 | +/0.6111 | 0.9324 | Substrate | Inhibitor |
|  | ZINC70692191 | +/0.8092 | +/0.9962 | -/0.6533 | 0.9732 | Substrate | Inhibitor |
|  | ZINC70692310 | +/0.6601 | +/1.0000 | -/0.6481 | 0.9829 | Substrate | Inhibitor |
|  | ZINC70692371 | -/0.7859 | -/0.7734 | -/0.6113 | 0.4352 | Substrate | Non-Inhibitor |
|  | **ZINC70699156** | +/0.5821 | +/0.8389 | -/0.5891 | 0.9154 | Non-Substrate | Non-Inhibitor |
|  | ZINC70699175 | +/0.7894 | +/0.9946 | -/0.6849 | 0.8365 | Substrate | Non-Inhibitor |
|  | ZINC70699179 | +/0.7894 | +/0.9946 | -/0.6849 | 0.8365 | Substrate | Non-Inhibitor |
|  | ZINC70699730 | +/0.5845 | +/0.9360 | -/0.7027 | 0.6836 | Substrate | Non-Inhibitor |
|  | **ZINC70699739** | +/0.5942 | +/0.9420 | -/0.6315 | 0.7923 | Non-Substrate | Non-Inhibitor |
|  | ZINC70699803 | +/0.8528 | +/0.9957 | -/0.6538 | 0.9501 | Substrate | Non-Inhibitor |
|  | ZINC70699952 | +/0.6424 | +/0.9974 | -/0.6129 | 0.9392 | Substrate | Inhibitor |
|  | **ZINC70700165** | +/0.8112 | +/0.9102 | -/0.6787 | 0.5865 | Non-Substrate | Non-Inhibitor |
|  | ZINC70700167 | +/0.8955 | +/0.9959 | -/0.6573 | 0.9449 | Substrate | Non-Inhibitor |
|  | ZINC70700233 | +/0.8528 | +/0.9957 | -/0.6538 | 0.9501 | Substrate | Non-Inhibitor |
|  | **ZINC70700623** | +/0.5821 | +/0.8389 | -/0.5891 | 0.9154 | Non-Substrate | Non-Inhibitor |
|  | **ZINC70700682** | +/0.5942 | +/0.9420 | -/0.6315 | 0.7923 | Non-Substrate | Non-Inhibitor |
|  | ZINC70700741 | -/0.6344 | +/1.0000 | -/0.6583 | 0.9768 | Substrate | Inhibitor |
|  | ZINC70700757 | -/0.6344 | +/1.0000 | -/0.6583 | 0.9768 | Substrate | Inhibitor |
|  | ZINC70700768 | +/0.8546 | +/1.0000 | -/0.6445 | 1.1965 | Substrate | Inhibitor |
|  | ZINC70700772 | +/0.5617 | +/0.9953 | -/0.6299 | 0.8843 | Substrate | Inhibitor |
|  | ZINC70700789 | +/0.5253 | +/0.9955 | -/0.6258 | 1.0400 | Substrate | Non-Inhibitor |
|  | ZINC70700816 | +/0.6049 | +/1.0000 | -/0.6445 | 1.0040 | Substrate | Inhibitor |
|  | ZINC70700844 | +/0.7730 | +/0.9974 | -/0.6786 | 0.9593 | Substrate | Non-Inhibitor |
|  | ZINC70700849 | +/0.9154 | +/0.9948 | -/0.5941 | 1.3080 | Substrate | Inhibitor |
|  | **ZINC70700931** | +/0.5821 | +/0.8389 | -/0.5891 | 0.9154 | Non-Substrate | Non-Inhibitor |
|  | ZINC70700934 | +/0.7497 | +/0.8807 | -/0.6639 | 0.7267 | Substrate | Non-Inhibitor |
|  | ZINC70700996 | +/0.7497 | +/0.8807 | -/0.6639 | 0.7267 | Substrate | Non-Inhibitor |
|  | ZINC70701006 | +/0.6849 | +/0.9962 | -/0.6816 | 1.0313 | Substrate | Non-Inhibitor |
|  | ZINC70701009 | +/0.6849 | +/0.9962 | -/0.6816 | 1.0313 | Substrate | Non-Inhibitor |
|  | **ZINC70701019** | +/0.5821 | +/0.8389 | -/0.5891 | 0.9154 | Non-Substrate | Non-Inhibitor |
|  | ZINC70701154 | +/0.9370 | +/0.9942 | -/0.6776 | 0.7671 | Non-Substrate | Non-Inhibitor |
|  | ZINC70701261 | +/0.8182 | +/1.0000 | -/0.6619 | 0.8223 | Substrate | Inhibitor |
|  | ZINC70701263 | +/0.8182 | +/1.0000 | -/0.6619 | 0.8223 | Substrate | Inhibitor |
|  | ZINC70701308 | +/0.5617 | +/0.9953 | -/0.6299 | 0.8843 | Substrate | Inhibitor |
|  | ZINC70701310 | -/0.5182 | +/0.9963 | -/0.6169 | 1.0386 | Substrate | Non-Inhibitor |
|  | ZINC70701627 | -/0.8612 | +/0.6490 | -/0.6676 | 0.7407 | Substrate | Non-Inhibitor |
|  | ZINC70701630 | -/0.8612 | +/0.6490 | -/0.6676 | 0.7407 | Substrate | Non-Inhibitor |
|  | ZINC70704409 | -/0.7290 | +/0.6477 | -/0.7537 | 0.3164 | Substrate | Non-Inhibitor |
|  | ZINC70704530 | -/0.8612 | +/0.6490 | -/0.6676 | 0.7407 | Substrate | Non-Inhibitor |
|  | ZINC70704538 | -/0.8612 | +/0.6490 | -/0.6676 | 0.7407 | Substrate | Non-Inhibitor |
|  | ZINC70704562 | +/0.8092 | +/0.9962 | -/0.6533 | 0.9732 | Substrate | Inhibitor |
|  | ZINC70704571 | -/0.8121 | -/0.6364 | -/0.6513 | 0.5636 | Substrate | Non-Inhibitor |
|  | ZINC70704576 | +/0.8709 | +/1.0000 | -/0.7284 | 0.6292 | Substrate | Inhibitor |
|  | ZINC70704593 | -/0.8011 | +/0.9201 | -/0.6535 | 0.4499 | Substrate | Inhibitor |
|  | ZINC70704643 | -/0.5982 | +/0.9680 | -/0.6500 | 0.8488 | Substrate | Non-Inhibitor |
|  | ZINC70704648 | -/0.5982 | +/0.9680 | -/0.6500 | 0.8488 | Substrate | Non-Inhibitor |
|  | ZINC70704650 | -/0.5964 | +/0.9708 | -/0.6595 | 0.8215 | Substrate | Non-Inhibitor |
|  | ZINC70704667 | -/0.5613 | +/0.8650 | -/0.5963 | 1.0582 | Substrate | Inhibitor |
|  | ZINC70704687 | -/0.6144 | +/0.7044 | -/0.6610 | 0.5147 | Substrate | Non-Inhibitor |
|  | ZINC70704696 | +/0.7826 | +/0.9690 | -/0.6578 | 0.1712 | Substrate | Non-Inhibitor |
|  | ZINC70704741 | +/0.9414 | +/1.0000 | -/0.6673 | 1.0124 | Non-Substrate | Non-Inhibitor |
|  | ZINC70704777 | -/0.5402 | +/0.9253 | -/0.6183 | 0.9572 | Non-Substrate | Non-Inhibitor |
|  | ZINC70704782 | +/0.9069 | +/1.0000 | -/0.6460 | 1.0642 | Substrate | Non-Inhibitor |
|  | ZINC70704817 | +/0.6976 | +/1.0000 | -/.6681 | 0.7006 | Substrate | Non-Inhibitor |
|  | ZINC70704820 | +/0.6976 | +/1.0000 | -/.6681 | 0.7006 | Substrate | Non-Inhibitor |
|  | ZINC70704831 | -/0.5384 | +/0.9940 | -/0.6460 | 0.8219 | Substrate | Inhibitor |
|  | ZINC70704924 | -/0.6699 | +/0.9722 | -/0.6522 | 0.7556 | Substrate | Non-Inhibitor |
|  | ZINC70704940 | -/0.6769 | +/0.8817 | -/0.6503 | 0.8501 | Substrate | Inhibitor |
|  | ZINC70704954 | +/0.7315 | +/0.9947 | -/0.6664 | 0.9171 | Substrate | Non-Inhibitor |
|  | ZINC70704963 | +/0.7315 | +/0.9947 | -/0.6664 | 0.9171 | Substrate | Non-Inhibitor |
|  | ZINC70704967 | +/0.7375 | +/1.0000 | -/0.6369 | 0.9456 | Substrate | Non-Inhibitor |
|  | ZINC70704970 | -/0.6288 | +/0.9932 | -/0.6480 | 0.9432 | Substrate | Inhibitor |
|  | **ZINC70704976** | +/0.5969 | +/0.8775 | -/0.6643 | 0.5493 | Non-Substrate | Non-Inhibitor |
|  | ZINC70704983 | +/0.8504 | +/0.9971 | -/0.6576 | 0.9952 | Substrate | Inhibitor |
|  | ZINC70705014 | +/0.5264 | +/0.9195 | -/0.6206 | 0.9323 | Substrate | Inhibitor |
|  | ZINC70705018 | +/0.5264 | +/0.9195 | -/0.6206 | 0.9323 | Substrate | Inhibitor |
|  | ZINC70705022 | -/0.5402 | +/0.9253 | -/0.6183 | 0.9572 | Non-Substrate | Non-Inhibitor |
|  | ZINC70705055 | +/0.8092 | +/0.9962 | -/0.6533 | 0.9732 | Substrate | Inhibitor |
|  | ZINC70705072 | +/0.7576 | +/1.0000 | -/0.6126 | 0.8859 | Substrate | Inhibitor |
|  | ZINC70705084 | +/0.7576 | +/1.0000 | -/0.6126 | 0.8859 | Substrate | Inhibitor |
|  | ZINC70705092 | +/0.6661 | +/1.0000 | +/0.5062 | 1.0859 | Substrate | Inhibitor |
|  | ZINC70705102 | -/0.5188 | +/0.7299 | -/0.6847 | 0.3675 | Substrate | Non-Inhibitor |
|  | ZINC70705211 | +/0.7128 | +/1.0000 | -/0.6773 | 1.1171 | Substrate | Non-Inhibitor |
|  | ZINC70705227 | -/0.6089 | +/0.5582 | -/0.7351 | 0.2804 | Non-Substrate | Non-Inhibitor |
|  | ZINC70705243 | -/0.8011 | +/0.9201 | -/0.6535 | 0.4499 | Substrate | Inhibitor |
|  | ZINC70705246 | -/0.8011 | +/0.9201 | -/0.6535 | 0.4499 | Substrate | Inhibitor |
|  | ZINC70705249 | -/0.8011 | +/0.9201 | -/0.6535 | 0.4499 | Substrate | Inhibitor |
|  | ZINC70705273 | +/0.8013 | +/1.0000 | -/0.6479 | 1.0202 | Substrate | Inhibitor |
|  | ZINC70705332 | -/0.7478 | +/0.7088 | -/0.6551 | 0.6709 | Non-Substrate | Non-Inhibitor |
|  | ZINC70705335 | -/0.7478 | +/0.7088 | -/0.6551 | 0.6709 | Non-Substrate | Non-Inhibitor |
|  | ZINC70705341 | +/0.8013 | +/1.0000 | -/0.6479 | 1.0202 | Substrate | Inhibitor |
|  | ZINC70705347 | -/0.7717 | +/0.9625 | -/0.6615 | 0.6417 | Substrate | Non-Inhibitor |
|  | ZINC70705373 | +/0.8709 | +/1.0000 | -/0.7284 | 0.6292 | Substrate | Non-Inhibitor |
|  | ZINC70705454 | -/0.5668 | +/0.8205 | -/0.6860 | 0.4434 | Substrate | Non-Inhibitor |
|  | ZINC70705469 | -/0.5769 | +/0.9673 | -/0.6457 | 0.6686 | Non-Substrate | Non-Inhibitor |
|  | ZINC70705472 | -/0.5769 | +/0.9673 | -/0.6457 | 0.6686 | Non-Substrate | Non-Inhibitor |
|  | ZINC70705481 | -/0.5402 | +/0.9253 | -/0.6183 | 0.9572 | Non-Substrate | Non-Inhibitor |
|  | ZINC70705530 | +/0.8766 | +/1.0000 | -/0.7185 | 0.6743 | Substrate | Non-Inhibitor |
|  | **ZINC70705576** | +/0.6305 | +/0.7305 | -/0.6967 | 0.1946 | Non-Substrate | Non-Inhibitor |
|  | ZINC70705586 | +/0.7352 | +/1.0000 | -/0.6805 | 0.8166 | Substrate | Non-Inhibitor |
|  | ZINC70705594 | +/0.8304 | +/1.0000 | -/0.6616 | 0.9254 | Substrate | Inhibitor |
|  | ZINC70705647 | -/0.5711 | +/0.9430 | -/0.6492 | 0.7656 | Non-Substrate | Non-Inhibitor |
|  | ZINC70705650 | -/0.8706 | +/0.6510 | -/0.7642 | 0.3034 | Substrate | Non-Inhibitor |
|  | ZINC70705678 | +/0.8153 | +/0.9946 | -/0.6481 | 0.9213 | Substrate | Inhibitor |
|  | ZINC70705686 | +/0.8013 | +/1.0000 | -/0.6479 | 1.0202 | Substrate | Inhibitor |
|  | ZINC70705694 | +/0.5152 | +/0.8395 | -/0.7120 | 0.2649 | Substrate | Non-Inhibitor |
|  | ZINC70705713 | +/0.8304 | +/1.0000 | -/0.6616 | 0.9254 | Substrate | Inhibitor |
|  | ZINC70705731 | +/0.5264 | +/0.9195 | -/0.6206 | 0.9323 | Substrate | Inhibitor |
|  | ZINC70705733 | +/0.5264 | +/0.9195 | -/0.6206 | 0.9323 | Substrate | Inhibitor |
|  | ZINC70705741 | +/0.6630 | +/0.9968 | -/0.6500 | 1.1060 | Substrate | Non-Inhibitor |
|  | ZINC70705766 | -/0.7478 | +/0.7088 | -/0.6551 | 0.6709 | Non-Substrate | Non-Inhibitor |
|  | ZINC70705771 | -/0.7478 | +/0.7088 | -/0.6551 | 0.6709 | Non-Substrate | Non-Inhibitor |
|  | ZINC70705789 | -/0.8121 | -/0.6364 | -/0.6513 | 0.5636 | Substrate | Non-Inhibitor |
|  | ZINC70705793 | -/0.8121 | -/0.6364 | -/0.6513 | 0.5636 | Substrate | Non-Inhibitor |
|  | ZINC70705797 | +/0.5856 | +/0.9958 | -/0.5698 | 1.2982 | Substrate | Inhibitor |
|  | ZINC70705801 | +/0.8709 | +/1.0000 | -/0.7284 | 0.6292 | Substrate | Non-Inhibitor |
|  | ZINC70705817 | +/0.6630 | +/0.9968 | -/0.6500 | 1.1060 | Substrate | Non-Inhibitor |
|  | ZINC70705820 | +/0.5000 | +/0.9429 | -/0.6961 | 0.4645 | Non-Substrate | Non-Inhibitor |
|  | ZINC70705851 | -/0.6176 | +/0.8801 | -/0.6851 | 0.5597 | Substrate | Non-Inhibitor |
|  | ZINC70705862 | +/0.7473 | +/0.9962 | -/0.6980 | 0.7457 | Substrate | Non-Inhibitor |
|  | ZINC70705876 | +/0.5365 | +/0.7883 | -/0.6521 | 0.6135 | Substrate | Non-Inhibitor |
|  | ZINC70705882 | -/0.6510 | +/0.9355 | -/0.6674 | 0.7357 | Substrate | Inhibitor |
|  | ZINC70705913 | -/0.7182 | +/0.9359 | -/0.6801 | 0.1985 | Substrate | Non-Inhibitor |
|  | ZINC70705940 | -/0.9201 | +/0.7215 | -/0.6532 | 0.3914 | Substrate | Non-Inhibitor |
|  | ZINC70705965 | -/0.7864 | +/0.9772 | -/0.6586 | 0.8878 | Substrate | Non-Inhibitor |
|  | ZINC70705983 | +/0.5000 | +/0.6686 | -/0.6794 | 0.3502 | Substrate | Non-Inhibitor |
|  | ZINC70706001 | -/0.5751 | +/0.9949 | -/0.6312 | 0.8314 | Substrate | Non-Inhibitor |
|  | ZINC70706036 | +/0.7875 | +/1.0000 | -/0.7058 | 0.7095 | Substrate | Non-Inhibitor |
|  | **ZINC70706110** | +/0.6098 | +/0.9205 | -/0.6875 | 0.4134 | Non-Substrate | Non-Inhibitor |
|  | **ZINC70706152** | +/0.6833 | +/0.9305 | -/0.6009 | 0.8678 | Non-Substrate | Non-Inhibitor |
|  | ZINC70706199 | -/0.7035 | +/0.9829 | -/0.6373 | 0.7403 | Substrate | Non-Inhibitor |
|  | ZINC70706205 | +/0.8092 | +/0.9962 | -/0.6533 | 0.9732 | Substrate | Inhibitor |
|  | ZINC70706216 | +/0.8092 | +/0.9962 | -/0.6533 | 0.9732 | Substrate | Inhibitor |
|  | ZINC70706255 | +/0.5912 | +/1.0000 | -/0.6402 | 0.7189 | Substrate | Inhibitor |
|  | ZINC70706259 | +/0.5912 | +/1.0000 | -/0.6402 | 0.7189 | Substrate | Inhibitor |
|  | ZINC70706263 | -/0.5613 | +/0.8650 | -/0.5963 | 1.0582 | Substrate | Inhibitor |
|  | ZINC70706269 | -/0.5613 | +/0.8650 | -/0.5963 | 1.0582 | Substrate | Inhibitor |
|  | ZINC70706273 | -/0.6769 | +/0.8817 | -/0.6503 | 0.8501 | Substrate | Inhibitor |
|  | ZINC70706301 | -/0.5188 | +/0.7299 | -/0.6847 | 0.3675 | Substrate | Non-Inhibitor |
|  | ZINC70706313 | +/0.8013 | +/1.0000 | -/0.6479 | 1.0202 | Substrate | Inhibitor |
|  | ZINC70706346 | -/0.5880 | +/0.8952 | -/0.6891 | 0.5144 | Substrate | Non-Inhibitor |
|  | ZINC70706388 | +/0.5805 | +/0.9969 | -/0.6921 | 0.7023 | Substrate | Inhibitor |
|  | ZINC70706410 | -/0.5880 | +/0.8952 | -/0.6891 | 0.5144 | Substrate | Non-Inhibitor |
|  | ZINC70706450 | +/0.8298 | +/1.0000 | -/0.6424 | 0.8836 | Substrate | Non-Inhibitor |
|  | ZINC70706454 | +/0.5495 | +/0.9951 | -/0.6671 | 0.7721 | Substrate | Inhibitor |
|  | ZINC70706476 | -/0.7692 | +/0.5995 | -/0.6584 | 0.6370 | Substrate | Non-Inhibitor |
|  | ZINC70706482 | -/0.7692 | +/0.5995 | -/0.6584 | 0.6370 | Substrate | Non-Inhibitor |
|  | ZINC70706497 | -/0.5613 | +/0.8650 | -/0.5963 | 1.0582 | Substrate | Inhibitor |
|  | ZINC70706500 | +/0.7966 | +/0.9870 | -/0.5560 | 0.9196 | Non-Substrate | Inhibitor |
|  | ZINC70706505 | +/0.7966 | +/0.9870 | -/0.5560 | 0.9196 | Non-Substrate | Inhibitor |
|  | ZINC70706527 | +/0.8766 | +/1.0000 | -/0.7185 | 0.6743 | Substrate | Non-Inhibitor |
|  | ZINC70706554 | +/0.6124 | +/1.0000 | -/0.6567 | 0.9329 | Substrate | Inhibitor |
|  | ZINC70706561 | +/0.6124 | +/1.0000 | -/0.6567 | 0.9329 | Substrate | Inhibitor |
|  | ZINC70706606 | -/0.6510 | +/0.9355 | -/0.6674 | 0.7357 | Substrate | Inhibitor |
|  | ZINC70706646 | -/0.7692 | +/0.5995 | -/0.6584 | 0.6370 | Substrate | Non-Inhibitor |
|  | ZINC70706651 | -/0.7692 | +/0.5995 | -/0.6584 | 0.6370 | Substrate | Non-Inhibitor |
|  | ZINC70706660 | +/0.7954 | +/1.0000 | -/0.6994 | 0.7052 | Substrate | Non-Inhibitor |
|  | ZINC70706670 | -/0.5384 | +/0.9940 | -/0.6460 | 0.8219 | Substrate | Inhibitor |
|  | ZINC70706672 | -/0.5384 | +/0.9940 | -/0.6460 | 0.8219 | Substrate | Inhibitor |
|  | ZINC70706692 | -/0.8414 | +/0.8720 | -/0.6155 | 0.6881 | Substrate | Non-Inhibitor |
|  | ZINC70706700 | -/0.7633 | +/0.9766 | -/0.6500 | 0.3597 | Substrate | Non-Inhibitor |
|  | ZINC70706714 | -/0.5182 | +/1.0000 | -/0.6613 | 0.8744 | Substrate | Non-Inhibitor |
|  | ZINC70706747 | -/0.7017 | +/0.9505 | -/0.6833 | 0.5630 | Substrate | Non-Inhibitor |
|  | ZINC70706767 | -/0.7717 | +/0.9625 | -/0.6615 | 0.6417 | Substrate | Non-Inhibitor |
|  | ZINC70706775 | +/0.7823 | +/1.0000 | -/0.6149 | 0.9084 | Substrate | Non-Inhibitor |
|  | ZINC70706779 | +/0.7823 | +/1.0000 | -/0.6149 | 0.9084 | Substrate | Non-Inhibitor |
|  | ZINC70706783 | -/0.5668 | +/0.8205 | -/0.6860 | 0.4434 | Substrate | Non-Inhibitor |
|  | ZINC70706791 | +/0.7658 | +/1.0000 | -/0.6945 | 1.0284 | Substrate | Non-Inhibitor |
|  | ZINC70706801 | -/0.6510 | +/0.9355 | -/0.6674 | 0.7357 | Substrate | Inhibitor |
|  | ZINC70706824 | +/0.6072 | +/0.9963 | -/0.6772 | 0.7813 | Substrate | Inhibitor |
|  | ZINC70706830 | -/0.6448 | +/0.8002 | -/0.7137 | 0.2444 | Substrate | Non-Inhibitor |
|  | ZINC70706862 | +/0.9198 | +/0.9941 | +/0.5941 | 1.3295 | Substrate | Inhibitor |
|  | ZINC70706868 | -/0.5711 | +/0.9430 | -/0.6492 | 0.7656 | Non-Substrate | Non-Inhibitor |
|  | ZINC70706920 | -/0.6144 | +/0.7044 | -/0.6610 | 0.5147 | Substrate | Non-Inhibitor |
|  | ZINC70706955 | -/0.5188 | +/0.7299 | -/0.6847 | 0.3675 | Substrate | Non-Inhibitor |
|  | ZINC70706967 | +/0.9086 | +/0.9943 | -/0.6652 | 0.8692 | Non-Substrate | Non-Inhibitor |
|  | **ZINC70706981** | +/0.5471 | +/0.9568 | -/0.6409 | 0.8161 | Non-Substrate | Non-Inhibitor |
|  | **ZINC70706982** | +/0.5471 | +/0.9568 | -/0.6409 | 0.8161 | Non-Substrate | Non-Inhibitor |
|  | ZINC70706983 | -/0.5763 | +/1.0000 | -/0.6271 | 0.9302 | Substrate | Inhibitor |
|  | ZINC70707015 | -/0.8102 | -/0.5173 | -/0.7433 | 0.3963 | Substrate | Non-Inhibitor |
|  | ZINC70707063 | -/0.7017 | +/0.9505 | -/0.6833 | 0.5630 | Substrate | Non-Inhibitor |
|  | ZINC70707070 | +/0.7443 | +/0.9899 | -/0.6262 | 0.9702 | Substrate | Non-Inhibitor |
|  | ZINC70707076 | +/0.7443 | +/0.9899 | -/0.6262 | 0.9702 | Substrate | Non-Inhibitor |
|  | ZINC70707086 | +/0.8013 | +/1.0000 | -/0.6479 | 1.0202 | Substrate | Inhibitor |
|  | ZINC70707113 | -/0.8121 | -/0.6364 | -/0.6513 | 0.5636 | Substrate | Non-Inhibitor |
|  | ZINC70707115 | -/0.8121 | -/0.6364 | -/0.6513 | 0.5636 | Substrate | Non-Inhibitor |
|  | **ZINC70707119** | +/0.6565 | +/0.8331 | -/0.6801 | 0.4751 | Non-Substrate | Non-Inhibitor |
|  | ZINC70707131 | -/0.8678 | +/0.9738 | -/0.6762 | 0.2797 | Substrate | Inhibitor |
|  | ZINC70707132 | -/0.8678 | +/0.9738 | -/0.6762 | 0.2797 | Substrate | Inhibitor |
|  | **ZINC70707134** | +/0.6833 | +/0.9305 | -/0.6009 | 0.8678 | Non-Substrate | Non-Inhibitor |
|  | ZINC70707151 | -/0.6769 | +/0.8817 | -/0.6503 | 0.8501 | Substrate | Inhibitor |
|  | ZINC70707156 | +/0.7851 | +/1.0000 | -/0.6844 | 1.1255 | Substrate | Non-Inhibitor |
|  | ZINC70707164 | +/0.8304 | +/1.0000 | -/0.6616 | 0.9254 | Substrate | Inhibitor |
|  | ZINC70707172 | -/0.7280 | +/0.9607 | -/0.6540 | 0.7329 | Substrate | Non-Inhibitor |
|  | ZINC70707186 | +/0.5805 | +/0.9969 | -/0.6921 | 0.7023 | Substrate | Inhibitor |
|  | ZINC70707192 | -/0.8176 | +/0.9690 | -/0.6609 | 0.3166 | Substrate | Non-Inhibitor |
|  | ZINC70707205 | -/0.5402 | +/0.9253 | -/0.6183 | 0.9572 | Non-Substrate | Non-Inhibitor |
|  | ZINC70707209 | +/0.8504 | +/0.9971 | -/0.6576 | 0.9952 | Substrate | Inhibitor |
|  | ZINC70707221 | -/0.6510 | +/0.9355 | -/0.6674 | 0.7357 | Substrate | Inhibitor |
|  | ZINC70707222 | -/0.9566 | +/0.8909 | -/0.6678 | 0.2154 | Substrate | Inhibitor |
|  | ZINC70707229 | -/0.6140 | +/0.9876 | -/0.6189 | 0.9806 | Substrate | Inhibitor |
|  | ZINC70707251 | +/0.8153 | +/0.9946 | -/0.6481 | 0.9213 | Substrate | Inhibitor |
|  | ZINC70707260 | +/0.7114 | +/0.9811 | -/0.6555 | 0.2822 | Substrate | Non-Inhibitor |
|  | ZINC70707277 | -/0.5402 | +/0.9253 | -/0.6183 | 0.9572 | Non-Substrate | Non-Inhibitor |
|  | ZINC70707332 | +/0.5152 | +/0.8395 | -/0.7120 | 0.2649 | Substrate | Non-Inhibitor |
|  | ZINC70707349 | -/0.5613 | +/0.8650 | -/0.5963 | 1.0582 | Substrate | Inhibitor |
|  | ZINC70707371 | +/0.7443 | +/0.9899 | -/0.6262 | 0.9702 | Substrate | Non-Inhibitor |
|  | ZINC70707376 | +/0.7443 | +/0.9899 | -/0.6262 | 0.9702 | Substrate | Non-Inhibitor |
|  | ZINC70707395 | -/0.6694 | +/0.9932 | -/0.6317 | 0.9283 | Substrate | Inhibitor |
|  | ZINC70707416 | -/0.5630 | +/1.0000 | -/0.6245 | 0.8451 | Substrate | Non-Inhibitor |
|  | ZINC70707422 | +/0.8318 | +/0.9966 | -/0.6447 | 0.8895 | Substrate | Inhibitor |
|  | ZINC70707443 | +/0.9584 | +/1.0000 | -/0.6742 | 0.9313 | Non-Substrate | Non-Inhibitor |
|  | ZINC70707449 | +/0.8092 | +/0.9962 | -/0.6533 | 0.9732 | Substrate | Inhibitor |
|  | ZINC70707458 | +/0.6072 | +/0.9963 | -/0.6772 | 0.7813 | Substrate | Inhibitor |
|  | ZINC70707552 | +/0.5000 | +/0.6686 | -/0.6794 | 0.3502 | Substrate | Non-Inhibitor |
|  | ZINC70707580 | -/0.8102 | -/0.5173 | -/0.7433 | 0.3963 | Substrate | Non-Inhibitor |
|  | ZINC70707597 | +/0.5805 | +/0.9969 | -/0.6921 | 0.7023 | Substrate | Inhibitor |
|  | ZINC70707603 | +/0.7389 | +/1.0000 | -/0.6813 | 1.0926 | Substrate | Non-Inhibitor |
|  | ZINC70707609 | -/0.6769 | +/0.8817 | -/0.6503 | 0.8501 | Substrate | Inhibitor |
|  | ZINC70707624 | -/0.8121 | -/0.6364 | -/0.6513 | 0.5636 | Substrate | Non-Inhibitor |
|  | ZINC70707626 | -/0.8121 | -/0.6364 | -/0.6513 | 0.5636 | Substrate | Non-Inhibitor |
|  | **ZINC70707655** | +/0.5665 | +/0.7963 | -/0.6993 | 0.2190 | Non-Substrate | Non-Inhibitor |
|  | ZINC70707671 | +/0.8667 | +/1.0000 | -/0.6727 | 0.9166 | Substrate | Non-Inhibitor |
|  | ZINC70707750 | +/0.5495 | +/0.9951 | -/0.6671 | 0.7721 | Substrate | Inhibitor |
|  | ZINC70707762 | +/0.7576 | +/1.0000 | -/0.6126 | 0.8859 | Substrate | Inhibitor |
|  | ZINC70707772 | +/0.7576 | +/1.0000 | -/0.6126 | 0.8859 | Substrate | Inhibitor |
|  | ZINC70712128 | +/0.6804 | +/0.9793 | -/0.5680 | 0.9713 | Non-Substrate | Inhibitor |
|  | ZINC71382583 | +/0.7858 | +/0.9889 | +/0.7204 | 1.0277 | Non-Substrate | Inhibitor |
|  | ZINC77269479 | +/0.7558 | +/0.6037 | -/0.8294 | -0.7657 | Substrate | Inhibitor |
|  | ZINC79209918 | +/0.6876 | +/0.9932 | +/0.7122 | 1.0763 | Substrate | Inhibitor |
|  | ZINC79212807 | +/0.8176 | +/0.9893 | -/0.6499 | 0.2090 | Substrate | Non-Inhibitor |
|  | ZINC82185013 | +/0.6602 | +/0.9855 | -/0.5728 | 0.7998 | Substrate | Inhibitor |
|  | ZINC85536937 | +/0.9121 | +/0.7316 | -/0.5946 | 0.4365 | Substrate | Non-Inhibitor |
|  | ZINC85552021 | -/0.5059 | -/0.9239 | -/0.6916 | -0.1106 | Substrate | Non-Inhibitor |
|  | ZINC85866826 | -/0.5654 | +/0.6494 | -/0.6366 | 0.2903 | Substrate | Inhibitor |
|  | ZINC85867137 | +/0.8919 | +/0.8924 | +/0.6327 | 0.9717 | Substrate | Inhibitor |
|  | ZINC85902344 | +/0.7117 | +/0.9763 | -/0.5728 | 0.9996 | Substrate | Inhibitor |
|  | **ZINC95100194** | +/0.7967 | +/0.9521 | +/0.7619 | 0.9265 | Non-Substrate | Inhibitor |
|  | ZINC95100209 | +/0.9089 | +/0.8159 | -/0.5590 | 0.7343 | Non-Substrate | Non-Inhibitor |
|  | ZINC95100330 | -/0.5173 | +/0.8950 | -/0.6322 | -0.0493 | Substrate | Non-Inhibitor |
|  | ZINC95100337 | +/0.6719 | +/0.9922 | -/0.5278 | 1.0004 | Non-Substrate | Inhibitor |
|  | ZINC95100338 | +/0.5196 | +/0.8863 | -/0.5311 | 0.9804 | Non-Substrate | Non-Inhibitor |
|  | ZINC95100378 | +/0.7054 | +/0.9872 | -/0.5522 | 1.0289 | Non-Substrate | Non-Inhibitor |
|  | ZINC95100379 | +/0.7197 | +/0.8750 | -/0.5182 | 0.9976 | Non-Substrate | Non-Inhibitor |
|  | ZINC95100380 | +/0.8836 | +/0.9880 | +/0.5000 | 1.1502 | Non-Substrate | Non-Inhibitor |
|  | ZINC95100381 | +/0.7529 | +/0.9849 | -/0.5772 | 0.9390 | Non-Substrate | Non-Inhibitor |
|  | ZINC95100382 | +/0.7394 | +/0.8828 | -/0.5284 | 0.8838 | Non-Substrate | Non-Inhibitor |
|  | ZINC95100383 | -/0.5683 | +/0.9834 | -/0.5476 | 0.9322 | Substrate | Non-Inhibitor |
|  | ZINC95101040 | +/0.6602 | +/0.9855 | -/0.5728 | 0.7998 | Substrate | Inhibitor |
|  | ZINC95101062 | +/0.8063 | +/0.9871 | -/0.7089 | 0.1903 | Substrate | Non-Inhibitor |
|  | ZINC95101078 | -/0.8441 | -/0.8035 | -/0.7301 | -0.0039 | Substrate | Inhibitor |
|  | ZINC95612014 | +/0.9844 | +/1.0000 | +/0.5128 | 1.5246 | Non-Substrate | Inhibitor |
|  | ZINC96316264 | +/0.6074 | +/0.9080 | +/0.5271 | 0.5720 | Substrate | Inhibitor |

**Supplementary Table S2.** *In-silico* Cyp450 enzyme metabolism profile was obtained from admetSAR server for selected 404 compounds. Selected compounds (20) for redocking were highlighted in bold.

| **Sr. No.** | **Zinc ID** | **CYP-2C9 substrate/inhibitor** | **CYP-2D6 substrate/inhibitor** | **CYP-3A4 substrate/inhibitor** | **CYP-1A2 inhibitor** | **CYP-2C19 inhibitor** | **CYP inhibitory promiscuity** |
| --- | --- | --- | --- | --- | --- | --- | --- |
|  | ZINC01530886 | Non-Substrate/ Inhibitor | Non-Substrate/ Non-Inhibitor | Substrate/ Non-Inhibitor | Inhibitor | Inhibitor | High |
|  | ZINC02096969 | Non-Substrate/ Non-Inhibitor | Non-Substrate/ Non-Inhibitor | Substrate/ Inhibitor | Inhibitor | Inhibitor | High |
|  | ZINC02097182 | Non-Substrate/ Non-Inhibitor | Non-Substrate/ Non-Inhibitor | Substrate/ Non-Inhibitor | Non-Inhibitor | Non-Inhibitor | High |
|  | ZINC02118796 | Non-Substrate/ Non-Inhibitor | Non-Substrate/ Non-Inhibitor | Substrate/ Non-Inhibitor | Non-Inhibitor | Non-Inhibitor | High |
|  | ZINC02121154 | Non-Substrate/ Non-Inhibitor | Non-Substrate/ Non-Inhibitor | Substrate/ Non-Inhibitor | Non-Inhibitor | Non-Inhibitor | High |
|  | ZINC02125476 | Non-Substrate/ Non-Inhibitor | Non-Substrate/ Non-Inhibitor | Substrate/ Non-Inhibitor | Non-Inhibitor | Non-Inhibitor | High |
|  | ZINC02128340 | Non-Substrate/ Non-Inhibitor | Non-Substrate/ Non-Inhibitor | Substrate/ Non-Inhibitor | Non-Inhibitor | Non-Inhibitor | Low |
|  | ZINC02128421 | Non-Substrate/ Non-Inhibitor | Non-Substrate/ Non-Inhibitor | Substrate/ Non-Inhibitor | Non-Inhibitor | Non-Inhibitor | Low |
|  | ZINC02128423 | Non-Substrate/ Non-Inhibitor | Non-Substrate/ Non-Inhibitor | Substrate/ Non-Inhibitor | Non-Inhibitor | Non-Inhibitor | Low |
|  | ZINC02128602 | Non-Substrate/ Inhibitor | Non-Substrate/ Non-Inhibitor | Substrate/ Inhibitor | Non-Inhibitor | Non-Inhibitor | High |
|  | ZINC02129853 | Non-Substrate/ Non-Inhibitor | Non-Substrate/ Non-Inhibitor | Substrate/ Non-Inhibitor | Non-Inhibitor | Non-Inhibitor | High |
|  | ZINC02129857 | Non-Substrate/ Non-Inhibitor | Non-Substrate/ Non-Inhibitor | Substrate/ Non-Inhibitor | Non-Inhibitor | Non-Inhibitor | Low |
|  | ZINC02130074 | Non-Substrate/ Inhibitor | Non-Substrate/ Non-Inhibitor | Substrate/ Inhibitor | Non-Inhibitor | Non-Inhibitor | High |
|  | ZINC02130079 | Non-Substrate/ Non-Inhibitor | Non-Substrate/ Non-Inhibitor | Substrate/ Non-Inhibitor | Non-Inhibitor | Non-Inhibitor | High |
|  | ZINC02130200 | Non-Substrate/ Non-Inhibitor | Non-Substrate/ Non-Inhibitor | Substrate/ Non-Inhibitor | Non-Inhibitor | Non-Inhibitor | Low |
|  | ZINC02130322 | Non-Substrate/ Non-Inhibitor | Non-Substrate/ Non-Inhibitor | Non-Substrate/ Non-Inhibitor | Non-Inhibitor | Non-Inhibitor | Low |
|  | ZINC02130539 | Non-Substrate/ Inhibitor | Non-Substrate/ Non-Inhibitor | Substrate/ Inhibitor | Non-Inhibitor | Non-Inhibitor | High |
|  | ZINC02130817 | Non-Substrate/ Non-Inhibitor | Non-Substrate/ Non-Inhibitor | Substrate/ Non-Inhibitor | Non-Inhibitor | Non-Inhibitor | Low |
|  | ZINC02130826 | Non-Substrate/ Inhibitor | Non-Substrate/ Non-Inhibitor | Substrate/ Inhibitor | Non-Inhibitor | Non-Inhibitor | High |
|  | ZINC02131176 | Non-Substrate/ Non-Inhibitor | Non-Substrate/ Non-Inhibitor | Substrate/ Non-Inhibitor | Non-Inhibitor | Non-Inhibitor | High |
|  | ZINC02131179 | Non-Substrate/ Non-Inhibitor | Non-Substrate/ Non-Inhibitor | Substrate/ Non-Inhibitor | Non-Inhibitor | Non-Inhibitor | High |
|  | ZINC02131227 | Non-Substrate/ Non-Inhibitor | Non-Substrate/ Non-Inhibitor | Substrate/ Non-Inhibitor | Non-Inhibitor | Non-Inhibitor | Low |
|  | ZINC02131415 | Non-Substrate/ Non-Inhibitor | Non-Substrate/ Non-Inhibitor | Substrate/ Non-Inhibitor | Non-Inhibitor | Non-Inhibitor | Low |
|  | ZINC02131893 | Non-Substrate/ Non-Inhibitor | Non-Substrate/ Non-Inhibitor | Substrate/ Non-Inhibitor | Non-Inhibitor | Non-Inhibitor | Low |
|  | ZINC02131897 | Non-Substrate/ Non-Inhibitor | Non-Substrate/ Non-Inhibitor | Substrate/ Non-Inhibitor | Non-Inhibitor | Non-Inhibitor | Low |
|  | ZINC02133098 | Non-Substrate/ Non-Inhibitor | Non-Substrate/ Non-Inhibitor | Non-Substrate/ Non-Inhibitor | Non-Inhibitor | Non-Inhibitor | High |
|  | ZINC02133383 | Non-Substrate/ Non-Inhibitor | Non-Substrate/ Non-Inhibitor | Substrate/ Inhibitor | Non-Inhibitor | Non-Inhibitor | High |
|  | ZINC02133431 | Non-Substrate/ Non-Inhibitor | Non-Substrate/ Non-Inhibitor | Non-Substrate/ Non-Inhibitor | Non-Inhibitor | Non-Inhibitor | Low |
|  | ZINC02133462 | Non-Substrate/ Non-Inhibitor | Non-Substrate/ Non-Inhibitor | Substrate/ Non-Inhibitor | Non-Inhibitor | Non-Inhibitor | Low |
|  | ZINC02133485 | Non-Substrate/ Non-Inhibitor | Non-Substrate/ Non-Inhibitor | Substrate/ Non-Inhibitor | Non-Inhibitor | Non-Inhibitor | Low |
|  | ZINC02133487 | Non-Substrate/ Non-Inhibitor | Non-Substrate/ Non-Inhibitor | Substrate/ Non-Inhibitor | Non-Inhibitor | Non-Inhibitor | Low |
|  | ZINC02134726 | Non-Substrate/ Non-Inhibitor | Non-Substrate/ Non-Inhibitor | Substrate/ Non-Inhibitor | Non-Inhibitor | Non-Inhibitor | Low |
|  | ZINC02134956 | Non-Substrate/ Non-Inhibitor | Non-Substrate/ Non-Inhibitor | Substrate/ Non-Inhibitor | Non-Inhibitor | Non-Inhibitor | Low |
|  | ZINC02135285 | Non-Substrate/ Non-Inhibitor | Non-Substrate/ Non-Inhibitor | Substrate/ Non-Inhibitor | Non-Inhibitor | Non-Inhibitor | Low |
|  | ZINC02135300 | Non-Substrate/ Non-Inhibitor | Non-Substrate/ Non-Inhibitor | Substrate/ Non-Inhibitor | Non-Inhibitor | Non-Inhibitor | Low |
|  | ZINC02135304 | Non-Substrate/ Non-Inhibitor | Non-Substrate/ Non-Inhibitor | Substrate/ Non-Inhibitor | Non-Inhibitor | Non-Inhibitor | Low |
|  | ZINC02135455 | Non-Substrate/ Non-Inhibitor | Non-Substrate/ Non-Inhibitor | Substrate/ Non-Inhibitor | Non-Inhibitor | Non-Inhibitor | Low |
|  | ZINC02135875 | Non-Substrate/ Non-Inhibitor | Non-Substrate/ Non-Inhibitor | Substrate/ Non-Inhibitor | Non-Inhibitor | Non-Inhibitor | High |
|  | ZINC02135983 | Non-Substrate/ Non-Inhibitor | Non-Substrate/ Non-Inhibitor | Substrate/ Non-Inhibitor | Non-Inhibitor | Non-Inhibitor | High |
|  | ZINC02137586 | Non-Substrate/ Non-Inhibitor | Non-Substrate/ Non-Inhibitor | Substrate/ Non-Inhibitor | Non-Inhibitor | Non-Inhibitor | Low |
|  | ZINC02137697 | Non-Substrate/ Non-Inhibitor | Non-Substrate/ Non-Inhibitor | Substrate/ Non-Inhibitor | Non-Inhibitor | Non-Inhibitor | Low |
|  | ZINC02137876 | Non-Substrate/ Non-Inhibitor | Non-Substrate/ Non-Inhibitor | Substrate/ Non-Inhibitor | Non-Inhibitor | Non-Inhibitor | Low |
|  | ZINC02145637 | Non-Substrate/  Inhibitor | Non-Substrate/ Non-Inhibitor | Substrate/ Inhibitor | Inhibitor | Non-Inhibitor | High |
|  | ZINC02146033 | Non-Substrate/  Inhibitor | Substrate/ Inhibitor | Substrate/ Inhibitor | Inhibitor | Non-Inhibitor | High |
|  | ZINC02146060 | Non-Substrate/  Inhibitor | Non-Substrate/  Inhibitor | Substrate/ Inhibitor | Inhibitor | Non-Inhibitor | High |
|  | ZINC02146088 | Non-Substrate/  Inhibitor | Substrate/ Inhibitor | Substrate/ Inhibitor | Inhibitor | Non-Inhibitor | High |
|  | ZINC02160816 | Non-Substrate/ Non-Inhibitor | Non-Substrate/ Non-Inhibitor | Substrate/ Non-Inhibitor | Non-Inhibitor | Non-Inhibitor | Low |
|  | ZINC02160958 | Non-Substrate/ Non-Inhibitor | Non-Substrate/ Non-Inhibitor | Substrate/ Non-Inhibitor | Non-Inhibitor | Non-Inhibitor | Low |
|  | ZINC02161189 | Non-Substrate/ Non-Inhibitor | Non-Substrate/ Non-Inhibitor | Substrate/ Non-Inhibitor | Non-Inhibitor | Non-Inhibitor | Low |
|  | ZINC02161190 | Non-Substrate/ Non-Inhibitor | Non-Substrate/ Non-Inhibitor | Substrate/ Non-Inhibitor | Non-Inhibitor | Non-Inhibitor | Low |
|  | ZINC02161303 | Non-Substrate/ Non-Inhibitor | Non-Substrate/ Non-Inhibitor | Substrate/ Non-Inhibitor | Non-Inhibitor | Non-Inhibitor | Low |
|  | ZINC03983911 | Non-Substrate/ Inhibitor | Non-Substrate/ Non-Inhibitor | Substrate/ Non-Inhibitor | Inhibitor | Non-Inhibitor | Low |
|  | ZINC04204381 | Non-Substrate/ Non-Inhibitor | Non-Substrate/ Non-Inhibitor | Substrate/ Inhibitor | Non-Inhibitor | Inhibitor | High |
|  | ZINC04268355 | Non-Substrate/ Non-Inhibitor | Non-Substrate/ Non-Inhibitor | Non-Substrate/ Non-Inhibitor | Non-Inhibitor | Non-Inhibitor | Low |
|  | ZINC04273402 | Non-Substrate/ Non-Inhibitor | Non-Substrate/ Non-Inhibitor | Substrate/ Inhibitor | Non-Inhibitor | Non-Inhibitor | Low |
|  | ZINC04281017 | Non-Substrate/ Non-Inhibitor | Non-Substrate/ Non-Inhibitor | Substrate/ Non-Inhibitor | Non-Inhibitor | Non-Inhibitor | Low |
|  | ZINC04292491 | Non-Substrate/ Inhibitor | Non-Substrate/ Non-Inhibitor | Non-Substrate/ Inhibitor | Non-Inhibitor | Inhibitor | High |
|  | ZINC04292705 | Non-Substrate/ Inhibitor | Non-Substrate/ Non-Inhibitor | Substrate/ Inhibitor | Non-Inhibitor | Inhibitor | High |
|  | ZINC04293318 | Non-Substrate/ Inhibitor | Non-Substrate/ Non-Inhibitor | Substrate/ Non-Inhibitor | Non-Inhibitor | Inhibitor | High |
|  | ZINC04293322 | Non-Substrate/ Inhibitor | Non-Substrate/ Non-Inhibitor | Substrate/ Non-Inhibitor | Non-Inhibitor | Inhibitor | High |
|  | ZINC04293326 | Non-Substrate/ Inhibitor | Non-Substrate/ Non-Inhibitor | Substrate/ Non-Inhibitor | Non-Inhibitor | Inhibitor | High |
|  | ZINC04293328 | Non-Substrate/ Inhibitor | Non-Substrate/ Non-Inhibitor | Substrate/ Inhibitor | Inhibitor | Inhibitor | High |
|  | ZINC04293329 | Non-Substrate/ Inhibitor | Non-Substrate/ Non-Inhibitor | Substrate/ Inhibitor | Inhibitor | Inhibitor | High |
|  | ZINC04293330 | Non-Substrate/ Inhibitor | Non-Substrate/ Non-Inhibitor | Substrate/ Inhibitor | Inhibitor | Inhibitor | High |
|  | ZINC04293377 | Non-Substrate/ Inhibitor | Non-Substrate/ Non-Inhibitor | Non-Substrate/ Inhibitor | Inhibitor | Inhibitor | High |
|  | ZINC04293484 | Non-Substrate/ Inhibitor | Non-Substrate/ Non-Inhibitor | Non-Substrate/ Inhibitor | Inhibitor | Inhibitor | High |
|  | ZINC04293487 | Non-Substrate/ Inhibitor | Non-Substrate/ Non-Inhibitor | Non-Substrate/ Non-Inhibitor | Inhibitor | Inhibitor | High |
|  | ZINC04293736 | Non-Substrate/ Inhibitor | Non-Substrate/ Non-Inhibitor | Substrate/ Inhibitor | Non-Inhibitor | Inhibitor | High |
|  | ZINC04691948 | Non-Substrate/ Inhibitor | Non-Substrate/ Non-Inhibitor | Substrate/ Non-Inhibitor | Inhibitor | Non-Inhibitor | High |
|  | ZINC04712260 | Non-Substrate/ Inhibitor | Non-Substrate/ Non-Inhibitor | Non-Substrate/ Inhibitor | Non-Inhibitor | Inhibitor | High |
|  | ZINC06500907 | Non-Substrate/ Inhibitor | Non-Substrate/ Non-Inhibitor | Substrate/ Inhibitor | Non-Inhibitor | Inhibitor | High |
|  | ZINC06500915 | Non-Substrate/ Inhibitor | Non-Substrate/ Non-Inhibitor | Substrate/ Inhibitor | Non-Inhibitor | Inhibitor | High |
|  | ZINC06631508 | Non-Substrate/ Inhibitor | Non-Substrate/ Non-Inhibitor | Substrate/ Non-Inhibitor | Non-Inhibitor | Inhibitor | High |
|  | ZINC08214433 | Non-Substrate/ Inhibitor | Non-Substrate/ Non-Inhibitor | Substrate/ Non-Inhibitor | Non-Inhibitor | Non-Inhibitor | High |
|  | ZINC08382321 | Non-Substrate/ Non-Inhibitor | Non-Substrate/ Non-Inhibitor | Non-Substrate/ Non-Inhibitor | Non-Inhibitor | Non-Inhibitor | Low |
|  | ZINC08382323 | Non-Substrate/ Non-Inhibitor | Non-Substrate/ Non-Inhibitor | Non-Substrate/ Non-Inhibitor | Non-Inhibitor | Non-Inhibitor | Low |
|  | ZINC08382324 | Non-Substrate/ Non-Inhibitor | Non-Substrate/ Non-Inhibitor | Non-Substrate/ Non-Inhibitor | Non-Inhibitor | Non-Inhibitor | Low |
|  | ZINC08398296 | Non-Substrate/ Non-Inhibitor | Non-Substrate/ Non-Inhibitor | Substrate/ Inhibitor | Non-Inhibitor | Non-Inhibitor | Low |
|  | ZINC08398409 | Non-Substrate/ Inhibitor | Non-Substrate/ Non-Inhibitor | Substrate/ Non-Inhibitor | Inhibitor | Non-Inhibitor | High |
|  | ZINC08790054 | Non-Substrate/ Inhibitor | Non-Substrate/ Non-Inhibitor | Substrate/ Non-Inhibitor | Non-Inhibitor | Non-Inhibitor | Low |
|  | ZINC08790412 | Non-Substrate/ Non-Inhibitor | Non-Substrate/ Non-Inhibitor | Substrate/ Non-Inhibitor | Non-Inhibitor | Non-Inhibitor | Low |
|  | ZINC08790736 | Non-Substrate/ Non-Inhibitor | Non-Substrate/ Non-Inhibitor | Substrate/ Non-Inhibitor | Non-Inhibitor | Non-Inhibitor | Low |
|  | ZINC08790787 | Non-Substrate/ Non-Inhibitor | Non-Substrate/ Non-Inhibitor | Substrate/ Non-Inhibitor | Non-Inhibitor | Non-Inhibitor | Low |
|  | ZINC08790849 | Non-Substrate/ Non-Inhibitor | Non-Substrate/ Non-Inhibitor | Substrate/ Non-Inhibitor | Non-Inhibitor | Non-Inhibitor | Low |
|  | ZINC08790961 | Non-Substrate/ Non-Inhibitor | Non-Substrate/ Non-Inhibitor | Non-Substrate/ Non-Inhibitor | Non-Inhibitor | Non-Inhibitor | Low |
|  | ZINC08791059 | Non-Substrate/ Non-Inhibitor | Non-Substrate/ Non-Inhibitor | Substrate/ Inhibitor | Non-Inhibitor | Non-Inhibitor | Low |
|  | ZINC08791123 | Non-Substrate/ Non-Inhibitor | Non-Substrate/ Non-Inhibitor | Non-Substrate/ Non-Inhibitor | Non-Inhibitor | Non-Inhibitor | Low |
|  | ZINC08791133 | Non-Substrate/ Non-Inhibitor | Non-Substrate/ Non-Inhibitor | Substrate/ Non-Inhibitor | Non-Inhibitor | Non-Inhibitor | Low |
|  | ZINC08791324 | Non-Substrate/ Non-Inhibitor | Non-Substrate/ Non-Inhibitor | Substrate/ Inhibitor | Non-Inhibitor | Non-Inhibitor | High |
|  | ZINC08917941 | Non-Substrate/ Inhibitor | Non-Substrate/ Non-Inhibitor | Non-Substrate/ Inhibitor | Inhibitor | Inhibitor | High |
|  | ZINC08918002 | Non-Substrate/ Inhibitor | Non-Substrate/ Non-Inhibitor | Non-Substrate/ Inhibitor | Inhibitor | Inhibitor | High |
|  | ZINC08918038 | Non-Substrate/ Non-Inhibitor | Non-Substrate/ Non-Inhibitor | Substrate/ Non-Inhibitor | Non-Inhibitor | Non-Inhibitor | Low |
|  | ZINC08918050 | Non-Substrate/ Non-Inhibitor | Non-Substrate/ Non-Inhibitor | Substrate/ Inhibitor | Non-Inhibitor | Non-Inhibitor | Low |
|  | ZINC08918440 | Non-Substrate/ Non-Inhibitor | Non-Substrate/ Non-Inhibitor | Substrate/ Non-Inhibitor | Non-Inhibitor | Non-Inhibitor | Low |
|  | ZINC09312660 | Non-Substrate/ Non-Inhibitor | Non-Substrate/ Non-Inhibitor | Substrate/ Non-Inhibitor | Non-Inhibitor | Non-Inhibitor | Low |
|  | ZINC09373722 | Non-Substrate/ Inhibitor | Non-Substrate/ Non-Inhibitor | Non-Substrate/ Non-Inhibitor | Non-Inhibitor | Inhibitor | High |
|  | ZINC11867664 | Non-Substrate/ Inhibitor | Non-Substrate/ Non-Inhibitor | Non-Substrate/ Non-Inhibitor | Inhibitor | Inhibitor | High |
|  | ZINC11868779 | Non-Substrate/ Inhibitor | Non-Substrate/ Non-Inhibitor | Non-Substrate/ Inhibitor | Inhibitor | Inhibitor | High |
|  | ZINC11868805 | Non-Substrate/ Inhibitor | Non-Substrate/ Non-Inhibitor | Non-Substrate/ Inhibitor | Inhibitor | Inhibitor | High |
|  | ZINC11868862 | Non-Substrate/ Inhibitor | Non-Substrate/ Non-Inhibitor | Substrate/ Inhibitor | Inhibitor | Inhibitor | High |
|  | ZINC11868946 | Non-Substrate/ Inhibitor | Non-Substrate/ Non-Inhibitor | Non-Substrate/ Non-Inhibitor | Inhibitor | Inhibitor | High |
|  | ZINC11869394 | Non-Substrate/ Inhibitor | Non-Substrate/ Non-Inhibitor | Non-Substrate/ Non-Inhibitor | Inhibitor | Inhibitor | High |
|  | ZINC11869400 | Non-Substrate/ Inhibitor | Non-Substrate/ Non-Inhibitor | Non-Substrate/ Inhibitor | Inhibitor | Inhibitor | High |
|  | ZINC11869425 | Non-Substrate/ Inhibitor | Non-Substrate/ Non-Inhibitor | Non-Substrate/ Inhibitor | Inhibitor | Inhibitor | High |
|  | ZINC12662395 | Non-Substrate/ Inhibitor | Non-Substrate/ Non-Inhibitor | Non-Substrate/ Inhibitor | Inhibitor | Inhibitor | High |
|  | ZINC12872711 | Non-Substrate/ Non-Inhibitor | Non-Substrate/ Non-Inhibitor | Substrate/ Non-Inhibitor | Non-Inhibitor | Non-Inhibitor | Low |
|  | ZINC12880349 | Non-Substrate/ Non-Inhibitor | Non-Substrate/ Non-Inhibitor | Substrate/ Non-Inhibitor | Non-Inhibitor | Non-Inhibitor | Low |
|  | ZINC12880848 | Non-Substrate/ Non-Inhibitor | Non-Substrate/ Non-Inhibitor | Substrate/ Inhibitor | Inhibitor | Inhibitor | High |
|  | ZINC12882432 | Non-Substrate/ Non-Inhibitor | Non-Substrate/ Non-Inhibitor | Substrate/ Non-Inhibitor | Non-Inhibitor | Non-Inhibitor | Low |
|  | ZINC12882846 | Non-Substrate/ Non-Inhibitor | Non-Substrate/ Non-Inhibitor | Substrate/ Non-Inhibitor | Non-Inhibitor | Non-Inhibitor | High |
|  | ZINC12883224 | Non-Substrate/ Inhibitor | Non-Substrate/ Non-Inhibitor | Non-Substrate/ Non-Inhibitor | Inhibitor | Non-Inhibitor | High |
|  | ZINC12883239 | Non-Substrate/ Non-Inhibitor | Non-Substrate/ Non-Inhibitor | Substrate/ Non-Inhibitor | Non-Inhibitor | Non-Inhibitor | Low |
|  | ZINC12883509 | Non-Substrate/ Non-Inhibitor | Non-Substrate/ Non-Inhibitor | Substrate/ Non-Inhibitor | Non-Inhibitor | Non-Inhibitor | Low |
|  | ZINC15953437 | Non-Substrate/ Inhibitor | Non-Substrate/ Non-Inhibitor | Substrate/ Inhibitor | Inhibitor | Inhibitor | High |
|  | **ZINC15968620** | Non-Substrate/ Non-Inhibitor | Non-Substrate/ Non-Inhibitor | Substrate/ Non-Inhibitor | Non-Inhibitor | Non-Inhibitor | Low |
|  | **ZINC15968622** | Non-Substrate/ Non-Inhibitor | Non-Substrate/ Non-Inhibitor | Substrate/ Non-Inhibitor | Non-Inhibitor | Non-Inhibitor | Low |
|  | ZINC18007499 | Non-Substrate/ Non-Inhibitor | Non-Substrate/ Non-Inhibitor | Substrate/ Non-Inhibitor | Non-Inhibitor | Non-Inhibitor | Low |
|  | ZINC18158134 | Non-Substrate/ Inhibitor | Non-Substrate/ Non-Inhibitor | Substrate/ Non-Inhibitor | Inhibitor | Non-Inhibitor | High |
|  | ZINC18163300 | Non-Substrate/ Inhibitor | Non-Substrate/ Non-Inhibitor | Substrate/ Non-Inhibitor | Non-Inhibitor | Non-Inhibitor | Low |
|  | ZINC19721276 | Non-Substrate/ Inhibitor | Non-Substrate/ Non-Inhibitor | Substrate/ Non-Inhibitor | Non-Inhibitor | Non-Inhibitor | Low |
|  | ZINC19866195 | Non-Substrate/ Inhibitor | Non-Substrate/ Non-Inhibitor | Substrate/ Non-Inhibitor | Non-Inhibitor | Non-Inhibitor | Low |
|  | ZINC22443609 | Non-Substrate/ Non-Inhibitor | Non-Substrate/ Non-Inhibitor | Substrate/ Non-Inhibitor | Non-Inhibitor | Inhibitor | High |
|  | ZINC26671872 | Non-Substrate/ Non-Inhibitor | Non-Substrate/ Non-Inhibitor | Non-Substrate/ Non-Inhibitor | Non-Inhibitor | Non-Inhibitor | Low |
|  | ZINC28539034 | Non-Substrate/ Inhibitor | Non-Substrate/ Non-Inhibitor | Substrate/ Non-Inhibitor | Inhibitor | Non-Inhibitor | Low |
|  | ZINC30724344 | Non-Substrate/ Inhibitor | Non-Substrate/ Non-Inhibitor | Substrate/ Non-Inhibitor | Inhibitor | Non-Inhibitor | Low |
|  | ZINC30725806 | Non-Substrate/ Non-Inhibitor | Non-Substrate/ Non-Inhibitor | Substrate/ Non-Inhibitor | Inhibitor | Non-Inhibitor | Low |
|  | ZINC30725812 | Non-Substrate/ Non-Inhibitor | Non-Substrate/ Non-Inhibitor | Substrate/ Non-Inhibitor | Non-Inhibitor | Non-Inhibitor | Low |
|  | ZINC32786262 | Non-Substrate/ Non-Inhibitor | Non-Substrate/ Non-Inhibitor | Substrate/ Non-Inhibitor | Non-Inhibitor | Non-Inhibitor | Low |
|  | ZINC32789745 | Non-Substrate/ Inhibitor | Non-Substrate/ Non-Inhibitor | Substrate/ Non-Inhibitor | Inhibitor | Non-Inhibitor | Low |
|  | ZINC38139950 | Non-Substrate/ Non-Inhibitor | Non-Substrate/ Non-Inhibitor | Substrate/ Non-Inhibitor | Non-Inhibitor | Non-Inhibitor | Low |
|  | ZINC38139967 | Non-Substrate/ Non-Inhibitor | Non-Substrate/ Non-Inhibitor | Substrate/ Inhibitor | Non-Inhibitor | Non-Inhibitor | Low |
|  | ZINC38139969 | Non-Substrate/ Non-Inhibitor | Non-Substrate/ Non-Inhibitor | Substrate/ Non-Inhibitor | Non-Inhibitor | Non-Inhibitor | Low |
|  | ZINC38139983 | Non-Substrate/ Non-Inhibitor | Non-Substrate/ Non-Inhibitor | Substrate/ Non-Inhibitor | Non-Inhibitor | Non-Inhibitor | Low |
|  | ZINC38140001 | Non-Substrate/ Non-Inhibitor | Non-Substrate/ Non-Inhibitor | Substrate/ Non-Inhibitor | Non-Inhibitor | Non-Inhibitor | Low |
|  | ZINC38140007 | Non-Substrate/ Non-Inhibitor | Non-Substrate/ Non-Inhibitor | Substrate/ Inhibitor | Non-Inhibitor | Non-Inhibitor | Low |
|  | ZINC38140019 | Non-Substrate/ Non-Inhibitor | Non-Substrate/ Non-Inhibitor | Substrate/ Inhibitor | Non-Inhibitor | Non-Inhibitor | Low |
|  | ZINC38140043 | Non-Substrate/ Non-Inhibitor | Non-Substrate/ Non-Inhibitor | Substrate/ Inhibitor | Non-Inhibitor | Non-Inhibitor | Low |
|  | ZINC38140045 | Non-Substrate/ Non-Inhibitor | Non-Substrate/ Non-Inhibitor | Substrate/ Inhibitor | Non-Inhibitor | Non-Inhibitor | Low |
|  | ZINC38140047 | Non-Substrate/ Non-Inhibitor | Non-Substrate/ Non-Inhibitor | Substrate/ Non-Inhibitor | Non-Inhibitor | Non-Inhibitor | Low |
|  | ZINC44459964 | Non-Substrate/ Non-Inhibitor | Non-Substrate/ Non-Inhibitor | Substrate/ Non-Inhibitor | Non-Inhibitor | Non-Inhibitor | Low |
|  | ZINC53276076 | Non-Substrate/ Inhibitor | Non-Substrate/ Inhibitor | Substrate/ Non-Inhibitor | Inhibitor | Non-Inhibitor | Low |
|  | ZINC53682947 | Non-Substrate/ Inhibitor | Non-Substrate/ Non-Inhibitor | Substrate/ Non-Inhibitor | Inhibitor | Non-Inhibitor | High |
|  | ZINC56871207 | Non-Substrate/ Non-Inhibitor | Non-Substrate/ Non-Inhibitor | Substrate/ Non-Inhibitor | Non-Inhibitor | Non-Inhibitor | Low |
|  | **ZINC65731330** | Non-Substrate/ Non-Inhibitor | Non-Substrate/ Non-Inhibitor | Non-Substrate/ Non-Inhibitor | Non-Inhibitor | Non-Inhibitor | Low |
|  | ZINC67903538 | Non-Substrate/ Non-Inhibitor | Non-Substrate/ Non-Inhibitor | Non-Substrate/ Non-Inhibitor | Non-Inhibitor | Non-Inhibitor | Low |
|  | ZINC67913695 | Non-Substrate/ Inhibitor | Non-Substrate/ Non-Inhibitor | Non-Substrate/ Non-Inhibitor | Non-Inhibitor | Non-Inhibitor | Low |
|  | ZINC68568464 | Non-Substrate/ Non-Inhibitor | Non-Substrate/ Non-Inhibitor | Non-Substrate/ Inhibitor | Inhibitor | Non-Inhibitor | Low |
|  | ZINC68581659 | Non-Substrate/ Inhibitor | Non-Substrate/ Non-Inhibitor | Substrate/ Inhibitor | Non-Inhibitor | Inhibitor | High |
|  | ZINC68581663 | Non-Substrate/ Inhibitor | Non-Substrate/ Non-Inhibitor | Substrate/ Inhibitor | Non-Inhibitor | Inhibitor | High |
|  | ZINC68581666 | Non-Substrate/ Inhibitor | Non-Substrate/ Non-Inhibitor | Substrate/ Inhibitor | Non-Inhibitor | Inhibitor | High |
|  | ZINC68603562 | Non-Substrate/ Non-Inhibitor | Non-Substrate/ Non-Inhibitor | Substrate/ Non-Inhibitor | Non-Inhibitor | Non-Inhibitor | High |
|  | ZINC68604313 | Non-Substrate/ Non-Inhibitor | Non-Substrate/ Non-Inhibitor | Non-Substrate/ Non-Inhibitor | Non-Inhibitor | Non-Inhibitor | Low |
|  | ZINC70670071 | Non-Substrate/ Non-Inhibitor | Non-Substrate/ Non-Inhibitor | Substrate/ Non-Inhibitor | Inhibitor | Non-Inhibitor | High |
|  | ZINC70673869 | Non-Substrate/ Inhibitor | Substrate/ Inhibitor | Substrate/ Inhibitor | Inhibitor | Non-Inhibitor | High |
|  | ZINC70686632 | Non-Substrate/ Non-Inhibitor | Non-Substrate/ Non-Inhibitor | Non-Substrate/ Inhibitor | Inhibitor | Inhibitor | High |
|  | ZINC70686670 | Non-Substrate/ Inhibitor | Non-Substrate/ Non-Inhibitor | Non-Substrate/ Inhibitor | Inhibitor | Inhibitor | High |
|  | ZINC70686752 | Non-Substrate/ Inhibitor | Non-Substrate/ Non-Inhibitor | Non-Substrate/ Inhibitor | Inhibitor | Inhibitor | High |
|  | ZINC70687241 | Non-Substrate/ Non-Inhibitor | Non-Substrate/ Non-Inhibitor | Substrate/ Non-Inhibitor | Non-Inhibitor | Non-Inhibitor | High |
|  | ZINC70687549 | Non-Substrate/ Inhibitor | Non-Substrate/ Non-Inhibitor | Substrate/ Inhibitor | Non-Inhibitor | Non-Inhibitor | High |
|  | ZINC70687967 | Non-Substrate/ Non-Inhibitor | Non-Substrate/ Non-Inhibitor | Substrate/ Inhibitor | Non-Inhibitor | Non-Inhibitor | High |
|  | ZINC70691607 | Non-Substrate/ Non-Inhibitor | Non-Substrate/ Non-Inhibitor | Substrate/ Non-Inhibitor | Inhibitor | Non-Inhibitor | Low |
|  | ZINC70692032 | Non-Substrate/ Non-Inhibitor | Non-Substrate/ Non-Inhibitor | Substrate/ Non-Inhibitor | Non-Inhibitor | Non-Inhibitor | Low |
|  | ZINC70692191 | Non-Substrate/ Non-Inhibitor | Non-Substrate/ Non-Inhibitor | Substrate/ Non-Inhibitor | Non-Inhibitor | Non-Inhibitor | High |
|  | ZINC70692310 | Non-Substrate/ Non-Inhibitor | Non-Substrate/ Non-Inhibitor | Substrate/ Non-Inhibitor | Non-Inhibitor | Non-Inhibitor | Low |
|  | ZINC70692371 | Non-Substrate/ Non-Inhibitor | Non-Substrate/ Non-Inhibitor | Substrate/ Inhibitor | Non-Inhibitor | Non-Inhibitor | Low |
|  | **ZINC70699156** | Non-Substrate/ Non-Inhibitor | Non-Substrate/ Non-Inhibitor | Substrate/ Non-Inhibitor | Non-Inhibitor | Non-Inhibitor | Low |
|  | ZINC70699175 | Non-Substrate/ Non-Inhibitor | Non-Substrate/ Non-Inhibitor | Substrate/ Non-Inhibitor | Non-Inhibitor | Non-Inhibitor | Low |
|  | ZINC70699179 | Non-Substrate/ Non-Inhibitor | Non-Substrate/ Non-Inhibitor | Substrate/ Non-Inhibitor | Non-Inhibitor | Non-Inhibitor | Low |
|  | ZINC70699730 | Non-Substrate/ Non-Inhibitor | Non-Substrate/ Non-Inhibitor | Substrate/ Non-Inhibitor | Non-Inhibitor | Non-Inhibitor | Low |
|  | **ZINC70699739** | Non-Substrate/ Non-Inhibitor | Non-Substrate/ Non-Inhibitor | Substrate/ Non-Inhibitor | Non-Inhibitor | Non-Inhibitor | Low |
|  | ZINC70699803 | Non-Substrate/ Inhibitor | Non-Substrate/ Non-Inhibitor | Non-Substrate/ Non-Inhibitor | Non-Inhibitor | Non-Inhibitor | High |
|  | ZINC70699952 | Non-Substrate/ Non-Inhibitor | Non-Substrate/ Non-Inhibitor | Substrate/ Non-Inhibitor | Non-Inhibitor | Non-Inhibitor | High |
|  | **ZINC70700165** | Non-Substrate/ Non-Inhibitor | Non-Substrate/ Non-Inhibitor | Substrate/ Non-Inhibitor | Non-Inhibitor | Non-Inhibitor | Low |
|  | ZINC70700167 | Non-Substrate/ Non-Inhibitor | Non-Substrate/ Non-Inhibitor | Substrate/ Non-Inhibitor | Non-Inhibitor | Non-Inhibitor | Low |
|  | ZINC70700233 | Non-Substrate/ Non-Inhibitor | Non-Substrate/ Non-Inhibitor | Substrate/ Non-Inhibitor | Inhibitor | Non-Inhibitor | High |
|  | **ZINC70700623** | Non-Substrate/ Non-Inhibitor | Non-Substrate/ Non-Inhibitor | Substrate/ Non-Inhibitor | Non-Inhibitor | Non-Inhibitor | Low |
|  | **ZINC70700682** | Non-Substrate/ Non-Inhibitor | Non-Substrate/ Non-Inhibitor | Substrate/ Non-Inhibitor | Non-Inhibitor | Non-Inhibitor | Low |
|  | ZINC70700741 | Non-Substrate/ Non-Inhibitor | Non-Substrate/ Non-Inhibitor | Substrate/ Non-Inhibitor | Non-Inhibitor | Non-Inhibitor | Low |
|  | ZINC70700757 | Non-Substrate/ Non-Inhibitor | Non-Substrate/ Non-Inhibitor | Substrate/ Non-Inhibitor | Non-Inhibitor | Non-Inhibitor | Low |
|  | ZINC70700768 | Non-Substrate/ Non-Inhibitor | Non-Substrate/ Non-Inhibitor | Substrate/ Non-Inhibitor | Non-Inhibitor | Non-Inhibitor | Low |
|  | ZINC70700772 | Non-Substrate/ Non-Inhibitor | Non-Substrate/ Non-Inhibitor | Substrate/ Non-Inhibitor | Non-Inhibitor | Non-Inhibitor | Low |
|  | ZINC70700789 | Non-Substrate/ Non-Inhibitor | Non-Substrate/ Non-Inhibitor | Substrate/ Non-Inhibitor | Non-Inhibitor | Non-Inhibitor | Low |
|  | ZINC70700816 | Non-Substrate/ Non-Inhibitor | Non-Substrate/ Non-Inhibitor | Substrate/ Non-Inhibitor | Non-Inhibitor | Non-Inhibitor | Low |
|  | ZINC70700844 | Non-Substrate/ Non-Inhibitor | Non-Substrate/ Non-Inhibitor | Substrate/ Non-Inhibitor | Non-Inhibitor | Non-Inhibitor | Low |
|  | ZINC70700849 | Non-Substrate/ Non-Inhibitor | Non-Substrate/ Non-Inhibitor | Substrate/ Non-Inhibitor | Non-Inhibitor | Non-Inhibitor | Low |
|  | **ZINC70700931** | Non-Substrate/ Non-Inhibitor | Non-Substrate/ Non-Inhibitor | Substrate/ Non-Inhibitor | Non-Inhibitor | Non-Inhibitor | Low |
|  | ZINC70700934 | Non-Substrate/ Non-Inhibitor | Non-Substrate/ Non-Inhibitor | Substrate/ Non-Inhibitor | Non-Inhibitor | Non-Inhibitor | High |
|  | ZINC70700996 | Non-Substrate/ Non-Inhibitor | Non-Substrate/ Non-Inhibitor | Substrate/ Non-Inhibitor | Non-Inhibitor | Non-Inhibitor | High |
|  | ZINC70701006 | Non-Substrate/ Non-Inhibitor | Non-Substrate/ Non-Inhibitor | Substrate/ Non-Inhibitor | Non-Inhibitor | Non-Inhibitor | Low |
|  | ZINC70701009 | Non-Substrate/ Non-Inhibitor | Non-Substrate/ Non-Inhibitor | Substrate/ Non-Inhibitor | Non-Inhibitor | Non-Inhibitor | Low |
|  | **ZINC70701019** | Non-Substrate/ Non-Inhibitor | Non-Substrate/ Non-Inhibitor | Substrate/ Non-Inhibitor | Non-Inhibitor | Non-Inhibitor | Low |
|  | ZINC70701154 | Non-Substrate/ Non-Inhibitor | Non-Substrate/ Non-Inhibitor | Substrate/ Non-Inhibitor | Non-Inhibitor | Non-Inhibitor | High |
|  | ZINC70701261 | Non-Substrate/ Inhibitor | Non-Substrate/ Non-Inhibitor | Substrate/ Non-Inhibitor | Non-Inhibitor | Non-Inhibitor | High |
|  | ZINC70701263 | Non-Substrate/ Inhibitor | Non-Substrate/ Non-Inhibitor | Substrate/ Non-Inhibitor | Non-Inhibitor | Non-Inhibitor | High |
|  | ZINC70701308 | Non-Substrate/ Non-Inhibitor | Non-Substrate/ Non-Inhibitor | Substrate/ Non-Inhibitor | Non-Inhibitor | Non-Inhibitor | Low |
|  | ZINC70701310 | Non-Substrate/ Non-Inhibitor | Non-Substrate/ Non-Inhibitor | Substrate/ Non-Inhibitor | Non-Inhibitor | Non-Inhibitor | Low |
|  | ZINC70701627 | Non-Substrate/ Non-Inhibitor | Non-Substrate/ Non-Inhibitor | Substrate/ Non-Inhibitor | Non-Inhibitor | Non-Inhibitor | Low |
|  | ZINC70701630 | Non-Substrate/ Non-Inhibitor | Non-Substrate/ Non-Inhibitor | Substrate/ Non-Inhibitor | Non-Inhibitor | Non-Inhibitor | Low |
|  | ZINC70704409 | Non-Substrate/ Non-Inhibitor | Non-Substrate/ Non-Inhibitor | Non-Substrate/ Non-Inhibitor | Non-Inhibitor | Non-Inhibitor | Low |
|  | ZINC70704530 | Non-Substrate/ Non-Inhibitor | Non-Substrate/ Non-Inhibitor | Substrate/ Non-Inhibitor | Non-Inhibitor | Non-Inhibitor | Low |
|  | ZINC70704538 | Non-Substrate/ Non-Inhibitor | Non-Substrate/ Non-Inhibitor | Substrate/ Non-Inhibitor | Non-Inhibitor | Non-Inhibitor | Low |
|  | ZINC70704562 | Non-Substrate/ Non-Inhibitor | Non-Substrate/ Non-Inhibitor | Substrate/ Non-Inhibitor | Non-Inhibitor | Non-Inhibitor | High |
|  | ZINC70704571 | Non-Substrate/ Non-Inhibitor | Non-Substrate/ Non-Inhibitor | Substrate/ Non-Inhibitor | Non-Inhibitor | Non-Inhibitor | Low |
|  | ZINC70704576 | Non-Substrate/ Non-Inhibitor | Non-Substrate/ Non-Inhibitor | Non-Substrate/ Non-Inhibitor | Non-Inhibitor | Non-Inhibitor | Low |
|  | ZINC70704593 | Non-Substrate/ Non-Inhibitor | Non-Substrate/ Non-Inhibitor | Substrate/ Inhibitor | Non-Inhibitor | Non-Inhibitor | Low |
|  | ZINC70704643 | Non-Substrate/ Non-Inhibitor | Non-Substrate/ Non-Inhibitor | Substrate/ Non-Inhibitor | Non-Inhibitor | Non-Inhibitor | Low |
|  | ZINC70704648 | Non-Substrate/ Non-Inhibitor | Non-Substrate/ Non-Inhibitor | Substrate/ Non-Inhibitor | Non-Inhibitor | Non-Inhibitor | Low |
|  | ZINC70704650 | Non-Substrate/ Non-Inhibitor | Non-Substrate/ Non-Inhibitor | Substrate/ Non-Inhibitor | Non-Inhibitor | Non-Inhibitor | Low |
|  | ZINC70704667 | Non-Substrate/ Non-Inhibitor | Non-Substrate/ Non-Inhibitor | Substrate/ Non-Inhibitor | Non-Inhibitor | Non-Inhibitor | Low |
|  | ZINC70704687 | Non-Substrate/ Non-Inhibitor | Non-Substrate/ Non-Inhibitor | Substrate/ Non-Inhibitor | Non-Inhibitor | Non-Inhibitor | Low |
|  | ZINC70704696 | Non-Substrate/ Non-Inhibitor | Non-Substrate/ Non-Inhibitor | Non-Substrate/ Non-Inhibitor | Non-Inhibitor | Non-Inhibitor | Low |
|  | ZINC70704741 | Non-Substrate/ Non-Inhibitor | Non-Substrate/ Non-Inhibitor | Substrate/ Inhibitor | Non-Inhibitor | Non-Inhibitor | High |
|  | ZINC70704777 | Non-Substrate/ Non-Inhibitor | Non-Substrate/ Non-Inhibitor | Substrate/ Non-Inhibitor | Non-Inhibitor | Non-Inhibitor | Low |
|  | ZINC70704782 | Non-Substrate/ Non-Inhibitor | Non-Substrate/ Non-Inhibitor | Substrate/ Inhibitor | Non-Inhibitor | Non-Inhibitor | High |
|  | ZINC70704817 | Non-Substrate/ Non-Inhibitor | Non-Substrate/ Non-Inhibitor | Substrate/ Non-Inhibitor | Non-Inhibitor | Non-Inhibitor | High |
|  | ZINC70704820 | Non-Substrate/ Non-Inhibitor | Non-Substrate/ Non-Inhibitor | Substrate/ Non-Inhibitor | Non-Inhibitor | Non-Inhibitor | High |
|  | ZINC70704831 | Non-Substrate/ Non-Inhibitor | Non-Substrate/ Non-Inhibitor | Substrate/ Non-Inhibitor | Non-Inhibitor | Non-Inhibitor | High |
|  | ZINC70704924 | Non-Substrate/ Non-Inhibitor | Non-Substrate/ Non-Inhibitor | Substrate/ Non-Inhibitor | Non-Inhibitor | Non-Inhibitor | Low |
|  | ZINC70704940 | Non-Substrate/ Non-Inhibitor | Non-Substrate/ Non-Inhibitor | Substrate/ Non-Inhibitor | Non-Inhibitor | Non-Inhibitor | Low |
|  | ZINC70704954 | Non-Substrate/ Non-Inhibitor | Non-Substrate/ Non-Inhibitor | Substrate/ Inhibitor | Non-Inhibitor | Non-Inhibitor | High |
|  | ZINC70704963 | Non-Substrate/ Non-Inhibitor | Non-Substrate/ Non-Inhibitor | Substrate/ Inhibitor | Non-Inhibitor | Non-Inhibitor | High |
|  | ZINC70704967 | Non-Substrate/ Inhibitor | Non-Substrate/ Non-Inhibitor | Substrate/ Inhibitor | Non-Inhibitor | Non-Inhibitor | High |
|  | ZINC70704970 | Non-Substrate/ Non-Inhibitor | Non-Substrate/ Non-Inhibitor | Substrate/ Inhibitor | Non-Inhibitor | Non-Inhibitor | High |
|  | **ZINC70704976** | Non-Substrate/ Non-Inhibitor | Non-Substrate/ Non-Inhibitor | Non-Substrate/ Non-Inhibitor | Non-Inhibitor | Non-Inhibitor | Low |
|  | ZINC70704983 | Non-Substrate/ Non-Inhibitor | Non-Substrate/ Non-Inhibitor | Substrate/ Non-Inhibitor | Non-Inhibitor | Non-Inhibitor | High |
|  | ZINC70705014 | Non-Substrate/ Non-Inhibitor | Non-Substrate/ Non-Inhibitor | Substrate/ Non-Inhibitor | Non-Inhibitor | Non-Inhibitor | Low |
|  | ZINC70705018 | Non-Substrate/ Non-Inhibitor | Non-Substrate/ Non-Inhibitor | Substrate/ Non-Inhibitor | Non-Inhibitor | Non-Inhibitor | Low |
|  | ZINC70705022 | Non-Substrate/ Non-Inhibitor | Non-Substrate/ Non-Inhibitor | Substrate/ Non-Inhibitor | Non-Inhibitor | Non-Inhibitor | Low |
|  | ZINC70705055 | Non-Substrate/ Non-Inhibitor | Non-Substrate/ Non-Inhibitor | Substrate/ Non-Inhibitor | Non-Inhibitor | Non-Inhibitor | High |
|  | ZINC70705072 | Non-Substrate/ Non-Inhibitor | Non-Substrate/ Non-Inhibitor | Substrate/ Non-Inhibitor | Non-Inhibitor | Non-Inhibitor | High |
|  | ZINC70705084 | Non-Substrate/ Non-Inhibitor | Non-Substrate/ Non-Inhibitor | Substrate/ Non-Inhibitor | Non-Inhibitor | Non-Inhibitor | High |
|  | ZINC70705092 | Non-Substrate/ Non-Inhibitor | Non-Substrate/ Non-Inhibitor | Substrate/ Inhibitor | Non-Inhibitor | Non-Inhibitor | Low |
|  | ZINC70705102 | Non-Substrate/ Non-Inhibitor | Non-Substrate/ Non-Inhibitor | Substrate/ Non-Inhibitor | Non-Inhibitor | Non-Inhibitor | Low |
|  | ZINC70705211 | Non-Substrate/ Non-Inhibitor | Non-Substrate/ Non-Inhibitor | Substrate/ Non-Inhibitor | Non-Inhibitor | Non-Inhibitor | Low |
|  | ZINC70705227 | Non-Substrate/ Non-Inhibitor | Non-Substrate/ Non-Inhibitor | Non-Substrate/ Non-Inhibitor | Non-Inhibitor | Non-Inhibitor | Low |
|  | ZINC70705243 | Non-Substrate/ Non-Inhibitor | Non-Substrate/ Non-Inhibitor | Substrate/ Non-Inhibitor | Non-Inhibitor | Non-Inhibitor | High |
|  | ZINC70705246 | Non-Substrate/ Non-Inhibitor | Non-Substrate/ Non-Inhibitor | Substrate/ Non-Inhibitor | Non-Inhibitor | Non-Inhibitor | High |
|  | ZINC70705249 | Non-Substrate/ Non-Inhibitor | Non-Substrate/ Non-Inhibitor | Substrate/ Non-Inhibitor | Non-Inhibitor | Non-Inhibitor | High |
|  | ZINC70705273 | Non-Substrate/ Inhibitor | Non-Substrate/ Non-Inhibitor | Substrate/ Inhibitor | Non-Inhibitor | Non-Inhibitor | High |
|  | ZINC70705332 | Non-Substrate/ Non-Inhibitor | Non-Substrate/ Non-Inhibitor | Substrate/ Non-Inhibitor | Non-Inhibitor | Non-Inhibitor | Low |
|  | ZINC70705335 | Non-Substrate/ Non-Inhibitor | Non-Substrate/ Non-Inhibitor | Substrate/ Non-Inhibitor | Non-Inhibitor | Non-Inhibitor | Low |
|  | ZINC70705341 | Non-Substrate/ Inhibitor | Non-Substrate/ Non-Inhibitor | Substrate/ Inhibitor | Non-Inhibitor | Non-Inhibitor | High |
|  | ZINC70705347 | Non-Substrate/ Non-Inhibitor | Non-Substrate/ Non-Inhibitor | Substrate/ Non-Inhibitor | Non-Inhibitor | Non-Inhibitor | Low |
|  | ZINC70705373 | Non-Substrate/ Non-Inhibitor | Non-Substrate/ Non-Inhibitor | Non-Substrate/ Non-Inhibitor | Non-Inhibitor | Non-Inhibitor | Low |
|  | ZINC70705454 | Non-Substrate/ Non-Inhibitor | Non-Substrate/ Non-Inhibitor | Substrate/ Non-Inhibitor | Non-Inhibitor | Non-Inhibitor | Low |
|  | ZINC70705469 | Non-Substrate/ Non-Inhibitor | Non-Substrate/ Non-Inhibitor | Substrate/ Non-Inhibitor | Non-Inhibitor | Non-Inhibitor | Low |
|  | ZINC70705472 | Non-Substrate/ Non-Inhibitor | Non-Substrate/ Non-Inhibitor | Substrate/ Non-Inhibitor | Non-Inhibitor | Non-Inhibitor | Low |
|  | ZINC70705481 | Non-Substrate/ Non-Inhibitor | Non-Substrate/ Non-Inhibitor | Substrate/ Non-Inhibitor | Non-Inhibitor | Non-Inhibitor | Low |
|  | ZINC70705530 | Non-Substrate/ Inhibitor | Non-Substrate/ Non-Inhibitor | Substrate/ Inhibitor | Non-Inhibitor | Non-Inhibitor | High |
|  | **ZINC70705576** | Non-Substrate/ Non-Inhibitor | Non-Substrate/ Non-Inhibitor | Non-Substrate/ Non-Inhibitor | Non-Inhibitor | Non-Inhibitor | Low |
|  | ZINC70705586 | Non-Substrate/ Inhibitor | Non-Substrate/ Non-Inhibitor | Substrate/ Inhibitor | Inhibitor | Inhibitor | High |
|  | ZINC70705594 | Non-Substrate/ Inhibitor | Non-Substrate/ Non-Inhibitor | Substrate/ Non-Inhibitor | Non-Inhibitor | Non-Inhibitor | High |
|  | ZINC70705647 | Non-Substrate/ Non-Inhibitor | Non-Substrate/ Non-Inhibitor | Substrate/ Non-Inhibitor | Non-Inhibitor | Non-Inhibitor | Low |
|  | ZINC70705650 | Non-Substrate/ Non-Inhibitor | Non-Substrate/ Non-Inhibitor | Non-Substrate/ Non-Inhibitor | Non-Inhibitor | Non-Inhibitor | Low |
|  | ZINC70705678 | Non-Substrate/ Inhibitor | Non-Substrate/ Non-Inhibitor | Substrate/ Non-Inhibitor | Non-Inhibitor | Non-Inhibitor | High |
|  | ZINC70705686 | Non-Substrate/ Inhibitor | Non-Substrate/ Non-Inhibitor | Substrate/ Non-Inhibitor | Non-Inhibitor | Non-Inhibitor | High |
|  | ZINC70705694 | Non-Substrate/ Non-Inhibitor | Non-Substrate/ Non-Inhibitor | Non-Substrate/ Non-Inhibitor | Non-Inhibitor | Non-Inhibitor | Low |
|  | ZINC70705713 | Non-Substrate/ Inhibitor | Non-Substrate/ Non-Inhibitor | Substrate/ Non-Inhibitor | Non-Inhibitor | Non-Inhibitor | High |
|  | ZINC70705731 | Non-Substrate/ Non-Inhibitor | Non-Substrate/ Non-Inhibitor | Substrate/ Non-Inhibitor | Non-Inhibitor | Non-Inhibitor | Low |
|  | ZINC70705733 | Non-Substrate/ Non-Inhibitor | Non-Substrate/ Non-Inhibitor | Substrate/ Non-Inhibitor | Non-Inhibitor | Non-Inhibitor | Low |
|  | ZINC70705741 | Non-Substrate/ Non-Inhibitor | Non-Substrate/ Non-Inhibitor | Substrate/ Non-Inhibitor | Non-Inhibitor | Non-Inhibitor | Low |
|  | ZINC70705766 | Non-Substrate/ Non-Inhibitor | Non-Substrate/ Non-Inhibitor | Substrate/ Non-Inhibitor | Non-Inhibitor | Non-Inhibitor | Low |
|  | ZINC70705771 | Non-Substrate/ Non-Inhibitor | Non-Substrate/ Non-Inhibitor | Substrate/ Non-Inhibitor | Non-Inhibitor | Non-Inhibitor | Low |
|  | ZINC70705789 | Non-Substrate/ Non-Inhibitor | Non-Substrate/ Non-Inhibitor | Substrate/ Non-Inhibitor | Non-Inhibitor | Non-Inhibitor | Low |
|  | ZINC70705793 | Non-Substrate/ Non-Inhibitor | Non-Substrate/ Non-Inhibitor | Substrate/ Non-Inhibitor | Non-Inhibitor | Non-Inhibitor | Low |
|  | ZINC70705797 | Non-Substrate/ Non-Inhibitor | Non-Substrate/ Non-Inhibitor | Substrate/ Non-Inhibitor | Non-Inhibitor | Non-Inhibitor | Low |
|  | ZINC70705801 | Non-Substrate/ Non-Inhibitor | Non-Substrate/ Non-Inhibitor | Non-Substrate/ Non-Inhibitor | Non-Inhibitor | Non-Inhibitor | Low |
|  | ZINC70705817 | Non-Substrate/ Non-Inhibitor | Non-Substrate/ Non-Inhibitor | Substrate/ Non-Inhibitor | Non-Inhibitor | Non-Inhibitor | Low |
|  | ZINC70705820 | Non-Substrate/ Non-Inhibitor | Non-Substrate/ Non-Inhibitor | Non-Substrate/ Non-Inhibitor | Non-Inhibitor | Non-Inhibitor | Low |
|  | ZINC70705851 | Non-Substrate/ Non-Inhibitor | Non-Substrate/ Non-Inhibitor | Substrate/ Non-Inhibitor | Non-Inhibitor | Non-Inhibitor | Low |
|  | ZINC70705862 | Non-Substrate/ Non-Inhibitor | Non-Substrate/ Non-Inhibitor | Substrate/ Non-Inhibitor | Non-Inhibitor | Non-Inhibitor | Low |
|  | ZINC70705876 | Non-Substrate/ Non-Inhibitor | Non-Substrate/ Non-Inhibitor | Substrate/ Non-Inhibitor | Non-Inhibitor | Non-Inhibitor | Low |
|  | ZINC70705882 | Non-Substrate/ Non-Inhibitor | Non-Substrate/ Non-Inhibitor | Substrate/ Non-Inhibitor | Non-Inhibitor | Non-Inhibitor | Low |
|  | ZINC70705913 | Non-Substrate/ Non-Inhibitor | Non-Substrate/ Non-Inhibitor | Substrate/ Non-Inhibitor | Non-Inhibitor | Non-Inhibitor | High |
|  | ZINC70705940 | Non-Substrate/ Non-Inhibitor | Non-Substrate/ Non-Inhibitor | Substrate/ Non-Inhibitor | Non-Inhibitor | Non-Inhibitor | Low |
|  | ZINC70705965 | Non-Substrate/ Non-Inhibitor | Non-Substrate/ Non-Inhibitor | Substrate/ Non-Inhibitor | Non-Inhibitor | Non-Inhibitor | Low |
|  | ZINC70705983 | Non-Substrate/ Non-Inhibitor | Non-Substrate/ Non-Inhibitor | Substrate/ Non-Inhibitor | Non-Inhibitor | Non-Inhibitor | Low |
|  | ZINC70706001 | Non-Substrate/ Inhibitor | Non-Substrate/ Non-Inhibitor | Substrate/ Inhibitor | Non-Inhibitor | Non-Inhibitor | High |
|  | ZINC70706036 | Non-Substrate/ Non-Inhibitor | Non-Substrate/ Non-Inhibitor | Substrate/ Inhibitor | Non-Inhibitor | Non-Inhibitor | High |
|  | **ZINC70706110** | Non-Substrate/ Non-Inhibitor | Non-Substrate/ Non-Inhibitor | Non-Substrate/ Non-Inhibitor | Non-Inhibitor | Non-Inhibitor | Low |
|  | **ZINC70706152** | Non-Substrate/ Non-Inhibitor | Non-Substrate/ Non-Inhibitor | Non-Substrate/ Non-Inhibitor | Non-Inhibitor | Non-Inhibitor | Low |
|  | ZINC70706199 | Non-Substrate/ Non-Inhibitor | Non-Substrate/ Non-Inhibitor | Substrate/ Non-Inhibitor | Non-Inhibitor | Non-Inhibitor | Low |
|  | ZINC70706205 | Non-Substrate/ Non-Inhibitor | Non-Substrate/ Non-Inhibitor | Substrate/ Non-Inhibitor | Non-Inhibitor | Non-Inhibitor | High |
|  | ZINC70706216 | Non-Substrate/ Non-Inhibitor | Non-Substrate/ Non-Inhibitor | Substrate/ Non-Inhibitor | Non-Inhibitor | Non-Inhibitor | High |
|  | ZINC70706255 | Non-Substrate/ Non-Inhibitor | Non-Substrate/ Non-Inhibitor | Substrate/ Non-Inhibitor | Non-Inhibitor | Non-Inhibitor | High |
|  | ZINC70706259 | Non-Substrate/ Non-Inhibitor | Non-Substrate/ Non-Inhibitor | Substrate/ Non-Inhibitor | Non-Inhibitor | Non-Inhibitor | High |
|  | ZINC70706263 | Non-Substrate/ Non-Inhibitor | Non-Substrate/ Non-Inhibitor | Substrate/ Non-Inhibitor | Non-Inhibitor | Non-Inhibitor | High |
|  | ZINC70706269 | Non-Substrate/ Non-Inhibitor | Non-Substrate/ Non-Inhibitor | Substrate/ Non-Inhibitor | Non-Inhibitor | Non-Inhibitor | Low |
|  | ZINC70706273 | Non-Substrate/ Non-Inhibitor | Non-Substrate/ Non-Inhibitor | Substrate/ Non-Inhibitor | Non-Inhibitor | Non-Inhibitor | Low |
|  | ZINC70706301 | Non-Substrate/ Inhibitor | Non-Substrate/ Non-Inhibitor | Substrate/ Inhibitor | Non-Inhibitor | Non-Inhibitor | High |
|  | ZINC70706313 | Non-Substrate/ Non-Inhibitor | Non-Substrate/ Non-Inhibitor | Substrate/ Non-Inhibitor | Non-Inhibitor | Non-Inhibitor | Low |
|  | ZINC70706346 | Non-Substrate/ Inhibitor | Non-Substrate/ Non-Inhibitor | Substrate/ Inhibitor | Non-Inhibitor | Non-Inhibitor | High |
|  | ZINC70706388 | Non-Substrate/ Inhibitor | Non-Substrate/ Non-Inhibitor | Substrate/ Inhibitor | Non-Inhibitor | Non-Inhibitor | High |
|  | ZINC70706410 | Non-Substrate/ Inhibitor | Non-Substrate/ Non-Inhibitor | Substrate/ Inhibitor | Non-Inhibitor | Non-Inhibitor | High |
|  | ZINC70706450 | Non-Substrate/ Non-Inhibitor | Non-Substrate/ Non-Inhibitor | Non-Substrate/ Inhibitor | Non-Inhibitor | Non-Inhibitor | High |
|  | ZINC70706454 | Non-Substrate/ Non-Inhibitor | Non-Substrate/ Non-Inhibitor | Substrate/ Inhibitor | Non-Inhibitor | Non-Inhibitor | High |
|  | ZINC70706476 | Non-Substrate/ Non-Inhibitor | Non-Substrate/ Non-Inhibitor | Non-Substrate/ Non-Inhibitor | Non-Inhibitor | Non-Inhibitor | Low |
|  | ZINC70706482 | Non-Substrate/ Non-Inhibitor | Non-Substrate/ Non-Inhibitor | Non-Substrate/ Non-Inhibitor | Non-Inhibitor | Non-Inhibitor | Low |
|  | ZINC70706497 | Non-Substrate/ Non-Inhibitor | Non-Substrate/ Non-Inhibitor | Substrate/ Non-Inhibitor | Non-Inhibitor | Non-Inhibitor | Low |
|  | ZINC70706500 | Non-Substrate/ Inhibitor | Non-Substrate/ Non-Inhibitor | Substrate/ Inhibitor | Non-Inhibitor | Non-Inhibitor | High |
|  | ZINC70706505 | Non-Substrate/ Inhibitor | Non-Substrate/ Non-Inhibitor | Substrate/ Inhibitor | Non-Inhibitor | Non-Inhibitor | High |
|  | ZINC70706527 | Non-Substrate/ Non-Inhibitor | Non-Substrate/ Non-Inhibitor | Substrate/ Inhibitor | Non-Inhibitor | Non-Inhibitor | High |
|  | ZINC70706554 | Non-Substrate/ Non-Inhibitor | Non-Substrate/ Non-Inhibitor | Substrate/ Non-Inhibitor | Non-Inhibitor | Non-Inhibitor | High |
|  | ZINC70706561 | Non-Substrate/ Non-Inhibitor | Non-Substrate/ Non-Inhibitor | Substrate/ Non-Inhibitor | Non-Inhibitor | Non-Inhibitor | High |
|  | ZINC70706606 | Non-Substrate/ Non-Inhibitor | Non-Substrate/ Non-Inhibitor | Substrate/ Non-Inhibitor | Non-Inhibitor | Non-Inhibitor | Low |
|  | ZINC70706646 | Non-Substrate/ Non-Inhibitor | Non-Substrate/ Non-Inhibitor | Non-Substrate/ Non-Inhibitor | Non-Inhibitor | Non-Inhibitor | Low |
|  | ZINC70706651 | Non-Substrate/ Non-Inhibitor | Non-Substrate/ Non-Inhibitor | Non-Substrate/ Non-Inhibitor | Non-Inhibitor | Non-Inhibitor | Low |
|  | ZINC70706660 | Non-Substrate/ Non-Inhibitor | Non-Substrate/ Non-Inhibitor | Substrate/ Inhibitor | Non-Inhibitor | Non-Inhibitor | High |
|  | ZINC70706670 | Non-Substrate/ Non-Inhibitor | Non-Substrate/ Non-Inhibitor | Substrate/ Non-Inhibitor | Non-Inhibitor | Non-Inhibitor | High |
|  | ZINC70706672 | Non-Substrate/ Non-Inhibitor | Non-Substrate/ Non-Inhibitor | Substrate/ Non-Inhibitor | Non-Inhibitor | Non-Inhibitor | High |
|  | ZINC70706692 | Non-Substrate/ Non-Inhibitor | Non-Substrate/ Non-Inhibitor | Substrate/ Inhibitor | Non-Inhibitor | Non-Inhibitor | High |
|  | ZINC70706700 | Non-Substrate/ Non-Inhibitor | Non-Substrate/ Non-Inhibitor | Substrate/ Non-Inhibitor | Non-Inhibitor | Non-Inhibitor | High |
|  | ZINC70706714 | Non-Substrate/ Inhibitor | Non-Substrate/ Non-Inhibitor | Substrate/ Non-Inhibitor | Non-Inhibitor | Non-Inhibitor | High |
|  | ZINC70706747 | Non-Substrate/ Non-Inhibitor | Non-Substrate/ Non-Inhibitor | Substrate/ Non-Inhibitor | Non-Inhibitor | Non-Inhibitor | Low |
|  | ZINC70706767 | Non-Substrate/ Non-Inhibitor | Non-Substrate/ Non-Inhibitor | Substrate/ Non-Inhibitor | Non-Inhibitor | Non-Inhibitor | Low |
|  | ZINC70706775 | Non-Substrate/ Non-Inhibitor | Non-Substrate/ Non-Inhibitor | Non-Substrate/ Non-Inhibitor | Non-Inhibitor | Non-Inhibitor | High |
|  | ZINC70706779 | Non-Substrate/ Non-Inhibitor | Non-Substrate/ Non-Inhibitor | Non-Substrate/ Non-Inhibitor | Non-Inhibitor | Non-Inhibitor | High |
|  | ZINC70706783 | Non-Substrate/ Non-Inhibitor | Non-Substrate/ Non-Inhibitor | Substrate/ Non-Inhibitor | Non-Inhibitor | Non-Inhibitor | Low |
|  | ZINC70706791 | Non-Substrate/ Non-Inhibitor | Non-Substrate/ Non-Inhibitor | Substrate/ Non-Inhibitor | Non-Inhibitor | Non-Inhibitor | Low |
|  | ZINC70706801 | Non-Substrate/ Non-Inhibitor | Non-Substrate/ Non-Inhibitor | Substrate/ Non-Inhibitor | Non-Inhibitor | Non-Inhibitor | Low |
|  | ZINC70706824 | Non-Substrate/ Non-Inhibitor | Non-Substrate/ Non-Inhibitor | Substrate/ Inhibitor | Non-Inhibitor | Non-Inhibitor | High |
|  | ZINC70706830 | Non-Substrate/ Non-Inhibitor | Non-Substrate/ Non-Inhibitor | Substrate/ Non-Inhibitor | Non-Inhibitor | Non-Inhibitor | Low |
|  | ZINC70706862 | Non-Substrate/ Inhibitor | Non-Substrate/ Non-Inhibitor | Substrate/ Inhibitor | Non-Inhibitor | Non-Inhibitor | High |
|  | ZINC70706868 | Non-Substrate/ Non-Inhibitor | Non-Substrate/ Non-Inhibitor | Substrate/ Non-Inhibitor | Non-Inhibitor | Non-Inhibitor | Low |
|  | ZINC70706920 | Non-Substrate/ Non-Inhibitor | Non-Substrate/ Non-Inhibitor | Substrate/ Non-Inhibitor | Non-Inhibitor | Non-Inhibitor | High |
|  | ZINC70706955 | Non-Substrate/ Non-Inhibitor | Non-Substrate/ Non-Inhibitor | Substrate/ Non-Inhibitor | Non-Inhibitor | Non-Inhibitor | Low |
|  | ZINC70706967 | Non-Substrate/ Non-Inhibitor | Non-Substrate/ Non-Inhibitor | Substrate/ Non-Inhibitor | Non-Inhibitor | Non-Inhibitor | High |
|  | **ZINC70706981** | Non-Substrate/ Non-Inhibitor | Non-Substrate/ Non-Inhibitor | Substrate/ Non-Inhibitor | Non-Inhibitor | Non-Inhibitor | Low |
|  | **ZINC70706982** | Non-Substrate/ Non-Inhibitor | Non-Substrate/ Non-Inhibitor | Substrate/ Non-Inhibitor | Non-Inhibitor | Non-Inhibitor | Low |
|  | ZINC70706983 | Non-Substrate/ Inhibitor | Non-Substrate/ Non-Inhibitor | Substrate/ Inhibitor | Non-Inhibitor | Non-Inhibitor | High |
|  | ZINC70707015 | Non-Substrate/ Non-Inhibitor | Non-Substrate/ Non-Inhibitor | Substrate/ Non-Inhibitor | Non-Inhibitor | Non-Inhibitor | Low |
|  | ZINC70707063 | Non-Substrate/ Non-Inhibitor | Non-Substrate/ Non-Inhibitor | Substrate/ Non-Inhibitor | Non-Inhibitor | Non-Inhibitor | Low |
|  | ZINC70707070 | Non-Substrate/ Non-Inhibitor | Non-Substrate/ Non-Inhibitor | Substrate/ Non-Inhibitor | Non-Inhibitor | Non-Inhibitor | Low |
|  | ZINC70707076 | Non-Substrate/ Inhibitor | Non-Substrate/ Non-Inhibitor | Substrate/ Inhibitor | Non-Inhibitor | Non-Inhibitor | High |
|  | ZINC70707086 | Non-Substrate/ Non-Inhibitor | Non-Substrate/ Non-Inhibitor | Substrate/ Non-Inhibitor | Non-Inhibitor | Non-Inhibitor | Low |
|  | ZINC70707113 | Non-Substrate/ Non-Inhibitor | Non-Substrate/ Non-Inhibitor | Substrate/ Non-Inhibitor | Non-Inhibitor | Non-Inhibitor | Low |
|  | ZINC70707115 | Non-Substrate/ Non-Inhibitor | Non-Substrate/ Non-Inhibitor | Substrate/ Non-Inhibitor | Non-Inhibitor | Non-Inhibitor | Low |
|  | **ZINC70707119** | Non-Substrate/ Non-Inhibitor | Non-Substrate/ Non-Inhibitor | Substrate/ Non-Inhibitor | Non-Inhibitor | Non-Inhibitor | Low |
|  | ZINC70707131 | Non-Substrate/ Non-Inhibitor | Non-Substrate/ Non-Inhibitor | Substrate/ Non-Inhibitor | Non-Inhibitor | Non-Inhibitor | Low |
|  | ZINC70707132 | Non-Substrate/ Non-Inhibitor | Non-Substrate/ Non-Inhibitor | Non-Substrate/ Non-Inhibitor | Non-Inhibitor | Non-Inhibitor | Low |
|  | **ZINC70707134** | Non-Substrate/ Non-Inhibitor | Non-Substrate/ Non-Inhibitor | Substrate/ Non-Inhibitor | Non-Inhibitor | Non-Inhibitor | Low |
|  | ZINC70707151 | Non-Substrate/ Non-Inhibitor | Non-Substrate/ Non-Inhibitor | Substrate/ Non-Inhibitor | Non-Inhibitor | Non-Inhibitor | Low |
|  | ZINC70707156 | Non-Substrate/ Inhibitor | Non-Substrate/ Non-Inhibitor | Substrate/ Non-Inhibitor | Non-Inhibitor | Non-Inhibitor | High |
|  | ZINC70707164 | Non-Substrate/ Non-Inhibitor | Non-Substrate/ Non-Inhibitor | Substrate/ Non-Inhibitor | Non-Inhibitor | Non-Inhibitor | Low |
|  | ZINC70707172 | Non-Substrate/ Inhibitor | Non-Substrate/ Non-Inhibitor | Substrate/ Inhibitor | Non-Inhibitor | Non-Inhibitor | High |
|  | ZINC70707186 | Non-Substrate/ Inhibitor | Non-Substrate/ Non-Inhibitor | Substrate/ Non-Inhibitor | Non-Inhibitor | Non-Inhibitor | High |
|  | ZINC70707192 | Non-Substrate/ Non-Inhibitor | Non-Substrate/ Non-Inhibitor | Substrate/ Non-Inhibitor | Non-Inhibitor | Non-Inhibitor | Low |
|  | ZINC70707205 | Non-Substrate/ Non-Inhibitor | Non-Substrate/ Non-Inhibitor | Substrate/ Non-Inhibitor | Non-Inhibitor | Non-Inhibitor | High |
|  | ZINC70707209 | Non-Substrate/ Non-Inhibitor | Non-Substrate/ Non-Inhibitor | Substrate/ Non-Inhibitor | Non-Inhibitor | Non-Inhibitor | Low |
|  | ZINC70707221 | Non-Substrate/ Non-Inhibitor | Non-Substrate/ Non-Inhibitor | Substrate/ Inhibitor | Non-Inhibitor | Non-Inhibitor | Low |
|  | ZINC70707222 | Non-Substrate/ Non-Inhibitor | Non-Substrate/ Non-Inhibitor | Substrate/ Inhibitor | Non-Inhibitor | Non-Inhibitor | High |
|  | ZINC70707229 | Non-Substrate/ Inhibitor | Non-Substrate/ Non-Inhibitor | Substrate/ Non-Inhibitor | Non-Inhibitor | Non-Inhibitor | High |
|  | ZINC70707251 | Non-Substrate/ Non-Inhibitor | Non-Substrate/ Non-Inhibitor | Non-Substrate/ Inhibitor | Non-Inhibitor | Non-Inhibitor | High |
|  | ZINC70707260 | Non-Substrate/ Non-Inhibitor | Non-Substrate/ Non-Inhibitor | Substrate/ Non-Inhibitor | Non-Inhibitor | Non-Inhibitor | Low |
|  | ZINC70707277 | Non-Substrate/ Non-Inhibitor | Non-Substrate/ Non-Inhibitor | Non-Substrate/ Non-Inhibitor | Non-Inhibitor | Non-Inhibitor | Low |
|  | ZINC70707332 | Non-Substrate/ Non-Inhibitor | Non-Substrate/ Non-Inhibitor | Substrate/ Non-Inhibitor | Non-Inhibitor | Non-Inhibitor | Low |
|  | ZINC70707349 | Non-Substrate/ Non-Inhibitor | Non-Substrate/ Non-Inhibitor | Substrate/ Inhibitor | Non-Inhibitor | Non-Inhibitor | High |
|  | ZINC70707371 | Non-Substrate/ Non-Inhibitor | Non-Substrate/ Non-Inhibitor | Substrate/ Inhibitor | Non-Inhibitor | Non-Inhibitor | High |
|  | ZINC70707376 | Non-Substrate/ Non-Inhibitor | Non-Substrate/ Non-Inhibitor | Substrate/ Inhibitor | Non-Inhibitor | Non-Inhibitor | High |
|  | ZINC70707395 | Non-Substrate/ Inhibitor | Non-Substrate/ Non-Inhibitor | Substrate/ Non-Inhibitor | Non-Inhibitor | Non-Inhibitor | High |
|  | ZINC70707416 | Non-Substrate/ Inhibitor | Non-Substrate/ Non-Inhibitor | Substrate/ Inhibitor | Non-Inhibitor | Non-Inhibitor | High |
|  | ZINC70707422 | Non-Substrate/ Non-Inhibitor | Non-Substrate/ Non-Inhibitor | Substrate/ Inhibitor | Non-Inhibitor | Non-Inhibitor | Low |
|  | ZINC70707443 | Non-Substrate/ Non-Inhibitor | Non-Substrate/ Non-Inhibitor | Substrate/ Inhibitor | Non-Inhibitor | Non-Inhibitor | Low |
|  | ZINC70707449 | Non-Substrate/ Non-Inhibitor | Non-Substrate/ Non-Inhibitor | Substrate/ Non-Inhibitor | Non-Inhibitor | Non-Inhibitor | High |
|  | ZINC70707458 | Non-Substrate/ Non-Inhibitor | Non-Substrate/ Non-Inhibitor | Substrate/ Inhibitor | Non-Inhibitor | Non-Inhibitor | High |
|  | ZINC70707552 | Non-Substrate/ Non-Inhibitor | Non-Substrate/ Non-Inhibitor | Substrate/ Non-Inhibitor | Non-Inhibitor | Non-Inhibitor | Low |
|  | ZINC70707580 | Non-Substrate/ Non-Inhibitor | Non-Substrate/ Non-Inhibitor | Substrate/ Non-Inhibitor | Non-Inhibitor | Non-Inhibitor | Low |
|  | ZINC70707597 | Non-Substrate/ Inhibitor | Non-Substrate/ Non-Inhibitor | Substrate/ Inhibitor | Non-Inhibitor | Non-Inhibitor | High |
|  | ZINC70707603 | Non-Substrate/ Non-Inhibitor | Non-Substrate/ Non-Inhibitor | Substrate/ Non-Inhibitor | Non-Inhibitor | Non-Inhibitor | Low |
|  | ZINC70707609 | Non-Substrate/ Non-Inhibitor | Non-Substrate/ Non-Inhibitor | Substrate/ Non-Inhibitor | Non-Inhibitor | Non-Inhibitor | Low |
|  | ZINC70707624 | Non-Substrate/ Non-Inhibitor | Non-Substrate/ Non-Inhibitor | Substrate/ Non-Inhibitor | Non-Inhibitor | Non-Inhibitor | Low |
|  | ZINC70707626 | Non-Substrate/ Non-Inhibitor | Non-Substrate/ Non-Inhibitor | Substrate/ Non-Inhibitor | Non-Inhibitor | Non-Inhibitor | Low |
|  | **ZINC70707655** | Non-Substrate/ Non-Inhibitor | Non-Substrate/ Non-Inhibitor | Non-Substrate/ Non-Inhibitor | Non-Inhibitor | Non-Inhibitor | Low |
|  | ZINC70707671 | Non-Substrate/ Non-Inhibitor | Non-Substrate/ Non-Inhibitor | Substrate/ Non-Inhibitor | Non-Inhibitor | Non-Inhibitor | Low |
|  | ZINC70707750 | Non-Substrate/Non-Inhibitor | Non-Substrate/ Non-Inhibitor | Substrate/ Inhibitor | Non-Inhibitor | Non-Inhibitor | High |
|  | ZINC70707762 | Non-Substrate/ Non-Inhibitor | Non-Substrate/ Non-Inhibitor | Substrate/ Non-Inhibitor | Non-Inhibitor | Non-Inhibitor | High |
|  | ZINC70707772 | Non-Substrate/ Non-Inhibitor | Non-Substrate/ Non-Inhibitor | Substrate/ Non-Inhibitor | Non-Inhibitor | Non-Inhibitor | High |
|  | ZINC70712128 | Non-Substrate/ Inhibitor | Non-Substrate/ Non-Inhibitor | Substrate/ Inhibitor | Non-Inhibitor | Inhibitor | High |
|  | ZINC71382583 | Non-Substrate/ Inhibitor | Non-Substrate/ Non-Inhibitor | Substrate/ Non-Inhibitor | Non-Inhibitor | Inhibitor | High |
|  | ZINC77269479 | Non-Substrate/ Inhibitor | Non-Substrate/ Non-Inhibitor | Non-Substrate/ Non-Inhibitor | Non-Inhibitor | Non-Inhibitor | Low |
|  | ZINC79209918 | Non-Substrate/ Inhibitor | Non-Substrate/ Non-Inhibitor | Non-Substrate/ Non-Inhibitor | Non-Inhibitor | Inhibitor | High |
|  | ZINC79212807 | Non-Substrate/ Non-Inhibitor | Non-Substrate/ Non-Inhibitor | Substrate/ Non-Inhibitor | Non-Inhibitor | Non-Inhibitor | Low |
|  | ZINC82185013 | Non-Substrate/ Inhibitor | Non-Substrate/ Non-Inhibitor | Substrate/ Inhibitor | Inhibitor | Inhibitor | High |
|  | ZINC85536937 | Non-Substrate/ Non-Inhibitor | Non-Substrate/ Non-Inhibitor | Substrate/ Inhibitor | Non-Inhibitor | Non-Inhibitor | Low |
|  | ZINC85552021 | Non-Substrate/ Non-Inhibitor | Non-Substrate/ Non-Inhibitor | Non-Substrate/ Non-Inhibitor | Non-Inhibitor | Non-Inhibitor | Low |
|  | ZINC85866826 | Non-Substrate/ Non-Inhibitor | Non-Substrate/ Non-Inhibitor | Substrate/ Inhibitor | Non-Inhibitor | Non-Inhibitor | High |
|  | ZINC85867137 | Non-Substrate/ Non-Inhibitor | Non-Substrate/ Non-Inhibitor | Substrate/ Non-Inhibitor | Non-Inhibitor | Inhibitor | Low |
|  | ZINC85902344 | Non-Substrate/ Inhibitor | Non-Substrate/ Non-Inhibitor | Substrate/ Inhibitor | Inhibitor | Inhibitor | High |
|  | **ZINC95100194** | Non-Substrate/ Non-Inhibitor | Non-Substrate/ Non-Inhibitor | Non-Substrate/ Non-Inhibitor | Inhibitor | Non-Inhibitor | Low |
|  | ZINC95100209 | Non-Substrate/ Inhibitor | Non-Substrate/ Non-Inhibitor | Non-Substrate/ Non-Inhibitor | Non-Inhibitor | Inhibitor | Low |
|  | ZINC95100330 | Non-Substrate/ Non-Inhibitor | Non-Substrate/ Non-Inhibitor | Substrate/ Non-Inhibitor | Non-Inhibitor | Non-Inhibitor | High |
|  | ZINC95100337 | Non-Substrate/ Inhibitor | Non-Substrate/ Non-Inhibitor | Substrate/ Inhibitor | Inhibitor | Inhibitor | High |
|  | ZINC95100338 | Non-Substrate/ Inhibitor | Non-Substrate/ Non-Inhibitor | Substrate/ Inhibitor | Non-Inhibitor | Inhibitor | High |
|  | ZINC95100378 | Non-Substrate/ Non-Inhibitor | Non-Substrate/ Non-Inhibitor | Non-Substrate/ Inhibitor | Inhibitor | Non-Inhibitor | High |
|  | ZINC95100379 | Non-Substrate/ Non-Inhibitor | Non-Substrate/ Non-Inhibitor | Substrate/ Inhibitor | Inhibitor | Inhibitor | High |
|  | ZINC95100380 | Non-Substrate/ Non-Inhibitor | Non-Substrate/ Non-Inhibitor | Non-Substrate/ Inhibitor | Inhibitor | Non-Inhibitor | High |
|  | ZINC95100381 | Non-Substrate/ Non-Inhibitor | Non-Substrate/ Non-Inhibitor | Non-Substrate/ Inhibitor | Inhibitor | Non-Inhibitor | High |
|  | ZINC95100382 | Non-Substrate/ Non-Inhibitor | Non-Substrate/ Non-Inhibitor | Non-Substrate/ Inhibitor | Inhibitor | Inhibitor | High |
|  | ZINC95100383 | Non-Substrate/ Non-Inhibitor | Non-Substrate/ Non-Inhibitor | Substrate/ Inhibitor | Inhibitor | Inhibitor | High |
|  | ZINC95101040 | Non-Substrate/ Inhibitor | Non-Substrate/ Non-Inhibitor | Substrate/ Inhibitor | Inhibitor | Inhibitor | High |
|  | ZINC95101062 | Non-Substrate/ Non-Inhibitor | Non-Substrate/ Non-Inhibitor | Non-Substrate/ Non-Inhibitor | Non-Inhibitor | Non-Inhibitor | Low |
|  | ZINC95101078 | Non-Substrate/ Non-Inhibitor | Non-Substrate/ Non-Inhibitor | Non-Substrate/ Non-Inhibitor | Non-Inhibitor | Non-Inhibitor | Low |
|  | ZINC95612014 | Non-Substrate/ Inhibitor | Non-Substrate/ Non-Inhibitor | Substrate/ Inhibitor | Inhibitor | Inhibitor | High |
|  | ZINC96316264 | Non-Substrate/ Non-Inhibitor | Non-Substrate/ Non-Inhibitor | Substrate/ Non-Inhibitor | Non-Inhibitor | Non-Inhibitor | Low |

**Supplementary Table S3.** *In-silico* toxicity, carcinogenicity and LD_50_ profile obtained from admetSAR server for selected 404 compounds. Selected compounds (20) for redocking were highlighted in bold.

| **Sr. No.** | **ZINC ID** | **AMES Toxicity** | **Carcinogen** | | **HERG inhibition** | **Acute Oral Toxicity** | **Rat LD_50_** |
| --- | --- | --- | --- | --- | --- | --- | --- |
|  | ZINC01530886 | Non-Toxic | Non-carcinogens | | Non-Inhibitor | II/0.5200 | 2.7875 |
|  | ZINC02096969 | Non-Toxic | Non-carcinogens | | Non-Inhibitor | III/0.5558 | 2.5634 |
|  | ZINC02097182 | Non-Toxic | Non-carcinogens | | Non-Inhibitor | III/0.5435 | 2.8119 |
|  | ZINC02118796 | Non-Toxic | Non-carcinogens | | Non-Inhibitor | III/0.5695 | 2.8890 |
|  | ZINC02121154 | Non-Toxic | Non-carcinogens | | Non-Inhibitor | III/0.5608 | 2.8858 |
|  | ZINC02125476 | Non-Toxic | Non-carcinogens | | Non-Inhibitor | III/0.5818 | 2.8595 |
|  | ZINC02128340 | Non-Toxic | Non-carcinogens | | Inhibitor | III/0.6635 | 2.3736 |
|  | ZINC02128421 | Non-Toxic | Non-carcinogens | | Non-Inhibitor | III/0.5383 | 3.0148 |
|  | ZINC02128423 | Non-Toxic | Non-carcinogens | | Non-Inhibitor | III/0.5383 | 3.0148 |
|  | ZINC02128602 | Non-Toxic | Non-carcinogens | | Inhibitor | III/0.7253 | 2.2461 |
|  | ZINC02129853 | Non-Toxic | Non-carcinogens | | Non-Inhibitor | III/0.5711 | 2.8642 |
|  | ZINC02129857 | Non-Toxic | Non-carcinogens | | Non-Inhibitor | III/0.4585 | 3.1227 |
|  | ZINC02130074 | Non-Toxic | Non-carcinogens | | Inhibitor | III/0.6949 | 2.2117 |
|  | ZINC02130079 | Non-Toxic | Non-carcinogens | | Inhibitor | III/0.6948 | 2.3469 |
|  | ZINC02130200 | Non-Toxic | Non-carcinogens | | Inhibitor | III/0.6371 | 2.1886 |
|  | ZINC02130322 | Non-Toxic | Non-carcinogens | | Non-Inhibitor | III/0.5006 | 2.9595 |
|  | ZINC02130539 | Non-Toxic | Non-carcinogens | | Inhibitor | III/0.6774 | 2.5338 |
|  | ZINC02130817 | Non-Toxic | Non-carcinogens | | Inhibitor | III/0.7009 | 2.3771 |
|  | ZINC02130826 | Non-Toxic | Non-carcinogens | | Inhibitor | III/0.6550 | 2.3435 |
|  | ZINC02131176 | Non-Toxic | Non-carcinogens | | Non-Inhibitor | III/0.6320 | 2.5551 |
|  | ZINC02131179 | Non-Toxic | Non-carcinogens | | Non-Inhibitor | III/0.6320 | 2.5551 |
|  | ZINC02131227 | Non-Toxic | Non-carcinogens | | Non-Inhibitor | III/0.4585 | 3.1227 |
|  | ZINC02131415 | Toxic | Non-carcinogens | | Non-Inhibitor | III/0.6235 | 2.4596 |
|  | ZINC02131893 | Non-Toxic | Non-carcinogens | | Inhibitor | III/0.6357 | 2.2647 |
|  | ZINC02131897 | Non-Toxic | Non-carcinogens | | Inhibitor | III/0.6357 | 2.2647 |
|  | ZINC02133098 | Non-Toxic | Non-carcinogens | | Non-Inhibitor | III/0.6454 | 2.4738 |
|  | ZINC02133383 | Non-Toxic | Non-carcinogens | | Inhibitor | III/0.6515 | 2.4541 |
|  | ZINC02133431 | Non-Toxic | Non-carcinogens | | Non-Inhibitor | III/0.5748 | 2.1551 |
|  | ZINC02133462 | Non-Toxic | Non-carcinogens | | Inhibitor | III/0.6367 | 2.3113 |
|  | ZINC02133485 | Non-Toxic | Non-carcinogens | | Non-Inhibitor | III/0.5500 | 2.9852 |
|  | ZINC02133487 | Non-Toxic | Non-carcinogens | | Non-Inhibitor | III/0.5500 | 2.9852 |
|  | ZINC02134726 | Non-Toxic | Non-carcinogens | | Non-Inhibitor | III/0.6238 | 2.5499 |
|  | ZINC02134956 | Non-Toxic | Non-carcinogens | | Inhibitor | III/0.6550 | 2.3435 |
|  | ZINC02135285 | Non-Toxic | Non-carcinogens | | Inhibitor | III/0.5624 | 2.7600 |
|  | ZINC02135300 | Non-Toxic | Non-carcinogens | | Non-Inhibitor | III/0.5010 | 3.0318 |
|  | ZINC02135304 | Non-Toxic | Non-carcinogens | | Non-Inhibitor | III/0.5010 | 3.0318 |
|  | ZINC02135455 | Non-Toxic | Non-carcinogens | | Non-Inhibitor | III/0.4839 | 3.0110 |
|  | ZINC02135875 | Non-Toxic | Non-carcinogens | | Inhibitor | III/0.6952 | 2.2386 |
|  | ZINC02135983 | Non-Toxic | Non-carcinogens | | Inhibitor | III/0.6654 | 2.3284 |
|  | ZINC02137586 | Non-Toxic | Non-carcinogens | | Non-Inhibitor | III/0.5321 | 2.9990 |
|  | ZINC02137697 | Non-Toxic | Non-carcinogens | | Inhibitor | III/0.6549 | 2.2299 |
|  | ZINC02137876 | Non-Toxic | Non-carcinogens | | Inhibitor | III/0.6842 | 2.1311 |
|  | ZINC02145637 | Non-Toxic | Non-carcinogens | | Non-Inhibitor | III/0.6613 | 2.8058 |
|  | ZINC02146033 | Non-Toxic | Non-carcinogens | | Non-Inhibitor | III/0.7060 | 2.7217 |
|  | ZINC02146060 | Non-Toxic | Non-carcinogens | | Inhibitor | III/0.6371 | 2.8157 |
|  | ZINC02146088 | Non-Toxic | Non-carcinogens | | Inhibitor | III/0.6345 | 2.9596 |
|  | ZINC02160816 | Non-Toxic | Non-carcinogens | | Inhibitor | III/0.6321 | 2.4741 |
|  | ZINC02160958 | Non-Toxic | Non-carcinogens | | Inhibitor | III/0.6369 | 2.5087 |
|  | ZINC02161189 | Non-Toxic | Non-carcinogens | | Non-Inhibitor | III/0.4840 | 3.0965 |
|  | ZINC02161190 | Non-Toxic | Non-carcinogens | | Non-Inhibitor | III/0.4840 | 3.0965 |
|  | ZINC02161303 | Non-Toxic | Non-carcinogens | | Inhibitor | III/0.7009 | 2.3771 |
|  | ZINC03983911 | Non-Toxic | Non-carcinogens | | Non-Inhibitor | III/0.5855 | 2.5745 |
|  | ZINC04204381 | Non-Toxic | Non-carcinogens | | Non-Inhibitor | III/0.6449 | 2.2960 |
|  | ZINC04268355 | Non-Toxic | Non-carcinogens | | Non-Inhibitor | III/0.4895 | 3.2833 |
|  | ZINC04273402 | Non-Toxic | Non-carcinogens | | Inhibitor | III/0.7066 | 2.6025 |
|  | ZINC04281017 | Toxic | Non-carcinogens | | Non-Inhibitor | III/0.5085 | 2.4704 |
|  | ZINC04292491 | Non-Toxic | Non-carcinogens | | Non-Inhibitor | III/0.4630 | 2.5735 |
|  | ZINC04292705 | Toxic | Non-carcinogens | | Non-Inhibitor | III/0.6168 | 2.6156 |
|  | ZINC04293318 | Toxic | Non-carcinogens | | Non-Inhibitor | III/0.6746 | 2.3157 |
|  | ZINC04293322 | Toxic | Non-carcinogens | | Non-Inhibitor | III/0.6746 | 2.3157 |
|  | ZINC04293326 | Toxic | Non-carcinogens | | Non-Inhibitor | III/0.6548 | 2.3788 |
|  | ZINC04293328 | Non-Toxic | Non-carcinogens | | Non-Inhibitor | III/0.6619 | 2.2815 |
|  | ZINC04293329 | Non-Toxic | Carcinogens | | Non-Inhibitor | III/0.6269 | 2.3407 |
|  | ZINC04293330 | Non-Toxic | Carcinogens | | Non-Inhibitor | III/0.4423 | 2.5403 |
|  | ZINC04293377 | Toxic | Non-carcinogens | | Non-Inhibitor | III/0.6586 | 2.1520 |
|  | ZINC04293484 | Toxic | Non-carcinogens | | Non-Inhibitor | III/0.5509 | 2.6548 |
|  | ZINC04293487 | Toxic | Non-carcinogens | | Non-Inhibitor | III/0.5402 | 2.5414 |
|  | ZINC04293736 | Toxic | Non-carcinogens | | Non-Inhibitor | III/0.7183 | 2.1503 |
|  | ZINC04691948 | Non-Toxic | Non-carcinogens | | Non-Inhibitor | III/0.5745 | 2.5997 |
|  | ZINC04712260 | Non-Toxic | Non-carcinogens | | Non-Inhibitor | III/0.4630 | 2.5735 |
|  | ZINC06500907 | Non-Toxic | Non-carcinogens | | Non-Inhibitor | III/0.6896 | 2.6935 |
|  | ZINC06500915 | Non-Toxic | Non-carcinogens | | Non-Inhibitor | III/0.6896 | 2.6935 |
|  | ZINC06631508 | Non-Toxic | Non-carcinogens | | Non-Inhibitor | III/0.6667 | 2.6980 |
|  | ZINC08214433 | Toxic | Non-carcinogens | | Non-Inhibitor | III/0.6728 | 2.1904 |
|  | ZINC08382321 | Toxic | Non-carcinogens | | Non-Inhibitor | III/0.5967 | 2.5350 |
|  | ZINC08382323 | Toxic | Non-carcinogens | | Non-Inhibitor | III/0.1504 | 2.5350 |
|  | ZINC08382324 | Toxic | Non-carcinogens | | Non-Inhibitor | III/0.1504 | 2.5350 |
|  | ZINC08398296 | Toxic | Non-carcinogens | | Non-Inhibitor | III/0.6366 | 2.3406 |
|  | ZINC08398409 | Non-Toxic | Non-carcinogens | | Non-Inhibitor | III/0.5900 | 2.5442 |
|  | ZINC08790054 | Non-Toxic | Non-carcinogens | | Non-Inhibitor | III/0.5803 | 2.2514 |
|  | ZINC08790412 | Non-Toxic | Non-carcinogens | | Non-Inhibitor | III/0.5975 | 2.2727 |
|  | ZINC08790736 | Non-Toxic | Non-carcinogens | | Non-Inhibitor | III/0.6588 | 2.4667 |
|  | ZINC08790787 | Non-Toxic | Non-carcinogens | | Inhibitor | III/0.6513 | 2.1881 |
|  | ZINC08790849 | Non-Toxic | Non-carcinogens | | Inhibitor | III/0.6513 | 2.1881 |
|  | ZINC08790961 | Non-Toxic | Non-carcinogens | | Inhibitor | III/0.6207 | 2.2134 |
|  | ZINC08791059 | Non-Toxic | Non-carcinogens | | Non-Inhibitor | III/0.6451 | 2.5002 |
|  | ZINC08791123 | Non-Toxic | Non-carcinogens | | Non-Inhibitor | III/0.5748 | 2.1551 |
|  | ZINC08791133 | Non-Toxic | Non-carcinogens | | Non-Inhibitor | III/0.6367 | 2.4353 |
|  | ZINC08791324 | Non-Toxic | Non-carcinogens | | Non-Inhibitor | III/0.6724 | 2.3367 |
|  | ZINC08917941 | Non- Toxic | Non-carcinogens | | Non-Inhibitor | III/0.4394 | 2.7514 |
|  | ZINC08918002 | Toxic | Non-carcinogens | | Non-Inhibitor | III/0.5134 | 2.6252 |
|  | ZINC08918038 | Non- Toxic | Non-carcinogens | | Non-Inhibitor | III/0.6544 | 2.6685 |
|  | ZINC08918050 | Non- Toxic | Non-carcinogens | | Inhibitor | III/0.6338 | 2.5271 |
|  | ZINC08918440 | Non- Toxic | Non-carcinogens | | Inhibitor | III/0.6457 | 2.6378 |
|  | ZINC09312660 | Non- Toxic | Non-carcinogens | | Non-Inhibitor | III/0.6529 | 2.6575 |
|  | ZINC09373722 | Non- Toxic | Non-carcinogens | | Non-Inhibitor | III/0.6529 | 2.6575 |
|  | ZINC11867664 | Non- Toxic | Non-carcinogens | | Non-Inhibitor | III/0.5890 | 3.0238 |
|  | ZINC11868779 | Non- Toxic | Non-carcinogens | | Non-Inhibitor | III/0.5927 | 2.6595 |
|  | ZINC11868805 | Non- Toxic | Non-carcinogens | | Non-Inhibitor | III/0.5987 | 2.8036 |
|  | ZINC11868862 | Toxic | Non-carcinogens | | Non-Inhibitor | III/0.4795 | 2.6528 |
|  | ZINC11868946 | Non- Toxic | Non-carcinogens | | Non-Inhibitor | III/0.5012 | 2.8891 |
|  | ZINC11869394 | Non- Toxic | Non-carcinogens | | Non-Inhibitor | III/0.6597 | 2.5482 |
|  | ZINC11869400 | Non- Toxic | Non-carcinogens | | Non-Inhibitor | III/0.6954 | 2.4279 |
|  | ZINC11869425 | Toxic | Non-carcinogens | | Non-Inhibitor | III/0.6234 | 2.4140 |
|  | ZINC12662395 | Non- Toxic | Non-carcinogens | | Non-Inhibitor | III/0.4394 | 2.7514 |
|  | ZINC12872711 | Non- Toxic | Non-carcinogens | | Inhibitor | III/0.6717 | 2.2768 |
|  | ZINC12880349 | Non- Toxic | Non-carcinogens | | Inhibitor | III/0.6210 | 2.5461 |
|  | ZINC12880848 | Non- Toxic | Non-carcinogens | | Non-Inhibitor | III/0.6631 | 2.2244 |
|  | ZINC12882432 | Non- Toxic | Non-carcinogens | | Non-Inhibitor | III/0.5660 | 2.5172 |
|  | ZINC12882846 | Non- Toxic | Non-carcinogens | | Inhibitor | III/0.6717 | 2.2768 |
|  | ZINC12883224 | Non- Toxic | Non-carcinogens | | Inhibitor | III/0.5073 | 2.5539 |
|  | ZINC12883239 | Non- Toxic | Non-carcinogens | | Inhibitor | III/0.6663 | 2.4016 |
|  | ZINC12883509 | Non- Toxic | Non-carcinogens | | Inhibitor | III/0.6210 | 2.3818 |
|  | ZINC15953437 | Toxic | Non-carcinogens | | Non-Inhibitor | III/0.6296 | 2.5869 |
|  | **ZINC15968620** | Non- Toxic | Non-carcinogens | | Non-Inhibitor | III/0.5943 | 2.2915 |
|  | **ZINC15968622** | Non- Toxic | Non-carcinogens | | Non-Inhibitor | III/0.5943 | 2.2915 |
|  | ZINC18007499 | Non- Toxic | Non-carcinogens | | Non-Inhibitor | III/0.6032 | 2.5815 |
|  | ZINC18158134 | Non- Toxic | Non-carcinogens | | Non-Inhibitor | III/0.5726 | 2.5621 |
|  | ZINC18163300 | Non- Toxic | Non-carcinogens | | Non-Inhibitor | III/0.5726 | 2.5621 |
|  | ZINC19721276 | Non- Toxic | Non-carcinogens | | Non-Inhibitor | III/0.5774 | 2.5475 |
|  | ZINC19866195 | Non- Toxic | Non-carcinogens | | Non-Inhibitor | III/0.6651 | 2.4402 |
|  | ZINC22443609 | Non- Toxic | Non-carcinogens | | Inhibitor | III/0.7264 | 2.2396 |
|  | ZINC26671872 | Non- Toxic | Non-carcinogens | | Non-Inhibitor | III/0.5767 | 2.5730 |
|  | ZINC28539034 | Non- Toxic | Non-carcinogens | | Non-Inhibitor | III/0.5703 | 2.5654 |
|  | ZINC30724344 | Non- Toxic | Non-carcinogens | | Non-Inhibitor | III/0.5437 | 2.6645 |
|  | ZINC30725806 | Non- Toxic | Non-carcinogens | | Non-Inhibitor | III/0.5892 | 2.6241 |
|  | ZINC30725812 | Non- Toxic | Non-carcinogens | | Non-Inhibitor | III/0.5892 | 2.6241 |
|  | ZINC32786262 | Non- Toxic | Non-carcinogens | | Non-Inhibitor | III/0.5733 | 2.5637 |
|  | ZINC32789745 | Non- Toxic | Non-carcinogens | | Non-Inhibitor | III/0.5892 | 2.6241 |
|  | ZINC38139950 | Non- Toxic | Non-carcinogens | | Inhibitor | III/0.5676 | 2.3155 |
|  | ZINC38139967 | Non- Toxic | Non-carcinogens | | Non-Inhibitor | III/0.6823 | 2.3381 |
|  | ZINC38139969 | Non- Toxic | Non-carcinogens | | Non-Inhibitor | III/0.6910 | 2.2937 |
|  | ZINC38139983 | Non- Toxic | Non-carcinogens | | Non-Inhibitor | III/0.6910 | 2.2937 |
|  | ZINC38140001 | Non- Toxic | Non-carcinogens | | Inhibitor | III/0.6567 | 2.1588 |
|  | ZINC38140007 | Non- Toxic | Non-carcinogens | | Inhibitor | III/0.5961 | 2.3243 |
|  | ZINC38140019 | Non- Toxic | Non-carcinogens | | Inhibitor | III/0.5961 | 2.3243 |
|  | ZINC38140043 | Non- Toxic | Non-carcinogens | | Inhibitor | III/0.6567 | 2.1588 |
|  | ZINC38140045 | Non- Toxic | Non-carcinogens | | Inhibitor | III/0.5719 | 2.1303 |
|  | ZINC38140047 | Non- Toxic | Non-carcinogens | | Inhibitor | III/0.5719 | 2.1303 |
|  | ZINC44459964 | Non- Toxic | Non-carcinogens | | Non-Inhibitor | III/0.6735 | 2.5170 |
|  | ZINC53276076 | Non- Toxic | Non-carcinogens | | Non-Inhibitor | III/0.5855 | 2.5745 |
|  | ZINC53682947 | Non-Toxic | Non-Carcinogens | | Non-Inhibitor | III/0.5544 | 2.6802 |
|  | ZINC56871207 | Non-Toxic | Non-Carcinogens | | Non-Inhibitor | III/0.5892 | 2.6241 |
|  | **ZINC65731330** | Non-Toxic | Non-Carcinogens | | Non-Inhibitor | III/0.5132 | 2.6536 |
|  | ZINC67903538 | Non-Toxic | Non-Carcinogens | | Non-Inhibitor | III/0.6853 | 2.6805 |
|  | ZINC67913695 | Toxic | Non-Carcinogens | | Non-Inhibitor | III/0.3483 | 3.2895 |
|  | ZINC68568464 | Non-Toxic | Non-Carcinogens | | Non-Inhibitor | III/0.5441 | 2.6921 |
|  | ZINC68581659 | Non-Toxic | Non-Carcinogens | | Non-Inhibitor | III/0.7501 | 2.4366 |
|  | ZINC68581663 | Non-Toxic | Non-Carcinogens | | Non-Inhibitor | III/0.7501 | 2.4366 |
|  | ZINC68581666 | Non-Toxic | Non-Carcinogens | | Non-Inhibitor | III/0.7501 | 2.4366 |
|  | ZINC68603562 | Toxic | Non-Carcinogens | | Non-Inhibitor | III/0.6282 | 2.5225 |
|  | ZINC68604313 | Non-Toxic | Non-Carcinogens | | Non-Inhibitor | III/0.6191 | 2.3418 |
|  | ZINC70670071 | Non-Toxic | Non-Carcinogens | | Inhibitor | III/0.6196 | 2.5751 |
|  | ZINC70673869 | Non-Toxic | Non-Carcinogens | | Inhibitor | III/0.6418 | 2.8382 |
|  | ZINC70686632 | Non-Toxic | Non-Carcinogens | | Non-Inhibitor | II/0.3801 | 2.8473 |
|  | ZINC70686670 | Non-Toxic | Non-Carcinogens | | Non-Inhibitor | III/0.5987 | 2.8036 |
|  | ZINC70686752 | Non-Toxic | Non-Carcinogens | | Non-Inhibitor | III/0.4394 | 2.7514 |
|  | ZINC70687241 | Non-Toxic | Non-Carcinogens | | Non-Inhibitor | III/0.6383 | 2.6514 |
|  | ZINC70687549 | Non-Toxic | Non-Carcinogens | | Non-Inhibitor | III/0.6632 | 2.5423 |
|  | ZINC70687967 | Non-Toxic | Non-Carcinogens | | Inhibitor | III/0.6457 | 2.6378 |
|  | ZINC70691607 | Non-Toxic | Non-Carcinogens | | Non-Inhibitor | III/0.6737 | 2.5407 |
|  | ZINC70692032 | Non-Toxic | Non-Carcinogens | | Non-Inhibitor | III/0.8109 | 2.4065 |
|  | ZINC70692191 | Non-Toxic | Non-Carcinogens | | Inhibitor | III/0.6952 | 2.2386 |
|  | ZINC70692310 | Non-Toxic | Non-Carcinogens | | Inhibitor | III/0.6369 | 2.5087 |
|  | ZINC70692371 | Non-Toxic | Non-Carcinogens | | Inhibitor | III/0.6417 | 2.4551 |
|  | **ZINC70699156** | Non-Toxic | Non-Carcinogens | | Non-Inhibitor | III/0.6241 | 2.3709 |
|  | ZINC70699175 | Toxic | Non-Carcinogens | | Non-Inhibitor | III/0.5946 | 2.3699 |
|  | ZINC70699179 | Toxic | Non-Carcinogens | | Non-Inhibitor | III/0.5946 | 2.3699 |
|  | ZINC70699730 | Non-Toxic | Non-Carcinogens | | Non-Inhibitor | III/0.6287 | 2.3489 |
|  | **ZINC70699739** | Non-Toxic | Non-Carcinogens | | Non-Inhibitor | III/0.6396 | 2.2138 |
|  | ZINC70699803 | Non-Toxic | Non-Carcinogens | | Non-Inhibitor | III/0.6803 | 2.1911 |
|  | ZINC70699952 | Non-Toxic | Non-Carcinogens | | Non-Inhibitor | III/0.6367 | 2.5634 |
|  | **ZINC70700165** | Non-Toxic | Non-Carcinogens | | Non-Inhibitor | III/0.6205 | 2.2701 |
|  | ZINC70700167 | Non-Toxic | Non-Carcinogens | | Non-Inhibitor | III/0.6066 | 2.2720 |
|  | ZINC70700233 | Non-Toxic | Non-Carcinogens | | Non-Inhibitor | III/0.6803 | 2.1911 |
|  | **ZINC70700623** | Non-Toxic | Non-Carcinogens | | Non-Inhibitor | III/0.6241 | 2.3709 |
|  | **ZINC70700682** | Non-Toxic | Non-Carcinogens | | Non-Inhibitor | III/0.6396 | 2.2138 |
|  | ZINC70700741 | Non-Toxic | Non-Carcinogens | | Inhibitor | III/0.6817 | 2.3817 |
|  | ZINC70700757 | Non-Toxic | Non-Carcinogens | | Inhibitor | III/0.6817 | 2.3817 |
|  | ZINC70700768 | Non-Toxic | Non-Carcinogens | | Non-Inhibitor | III/0.6238 | 2.5499 |
|  | ZINC70700772 | Non-Toxic | Non-Carcinogens | | Inhibitor | III/0.6560 | 2.3771 |
|  | ZINC70700789 | Non-Toxic | Non-Carcinogens | | Inhibitor | III/0.6842 | 2.1311 |
|  | ZINC70700816 | Non-Toxic | Non-Carcinogens | | Inhibitor | III/0.6184 | 2.5703 |
|  | ZINC70700844 | Non-Toxic | Non-Carcinogens | | Inhibitor | III/0.6113 | 2.1376 |
|  | ZINC70700849 | Non-Toxic | Non-Carcinogens | | Non-Inhibitor | III/0.6189 | 2.4300 |
|  | **ZINC70700931** | Non-Toxic | Non-Carcinogens | | Non-Inhibitor | III/0.6241 | 2.3709 |
|  | ZINC70700934 | Non-Toxic | Non-Carcinogens | | Non-Inhibitor | III/0.6576 | 2.2647 |
|  | ZINC70700996 | Non-Toxic | Non-Carcinogens | | Non-Inhibitor | III/0.6576 | 2.2647 |
|  | ZINC70701006 | Non-Toxic | Non-Carcinogens | | Inhibitor | III/0.5859 | 2.2181 |
|  | ZINC70701009 | Non-Toxic | Non-Carcinogens | | Inhibitor | III/0.5859 | 2.2181 |
|  | **ZINC70701019** | Non-Toxic | Non-Carcinogens | | Non-Inhibitor | III/0.6241 | 2.3709 |
|  | ZINC70701154 | Non-Toxic | Non-Carcinogens | | Non-Inhibitor | III/0.6341 | 2.3417 |
|  | ZINC70701261 | Non-Toxic | Non-Carcinogens | | Inhibitor | III/0.5522 | 2.6518 |
|  | ZINC70701263 | Non-Toxic | Non-Carcinogens | | Inhibitor | III/0.5522 | 2.6518 |
|  | ZINC70701308 | Non-Toxic | Non-Carcinogens | | Inhibitor | III/0.6560 | 2.3771 |
|  | ZINC70701310 | Non-Toxic | Non-Carcinogens | | Inhibitor | III/0.6740 | 2.0916 |
|  | ZINC70701627 | Non-Toxic | Non-Carcinogens | | Non-Inhibitor | III/0.6520 | 2.3699 |
|  | ZINC70701630 | Non-Toxic | Non-Carcinogens | | Non-Inhibitor | III/0.6520 | 2.3699 |
|  | ZINC70704409 | Non-Toxic | Non-Carcinogens | | Non-Inhibitor | III/0.6075 | 2.0806 |
|  | ZINC70704530 | Non-Toxic | Non-Carcinogens | | Non-Inhibitor | III/0.6520 | 2.3699 |
|  | ZINC70704538 | Non-Toxic | Non-Carcinogens | | Non-Inhibitor | III/0.6520 | 2.3699 |
|  | ZINC70704562 | Non-Toxic | Non-Carcinogens | | Inhibitor | III/0.6952 | 2.2386 |
|  | ZINC70704571 | Non-Toxic | Non-Carcinogens | | Non-Inhibitor | III/0.6474 | 2.3082 |
|  | ZINC70704576 | Non-Toxic | Non-Carcinogens | | Inhibitor | III/0.6365 | 2.3341 |
|  | ZINC70704593 | Non-Toxic | Non-Carcinogens | | Inhibitor | III/0.6693 | 2.4716 |
|  | ZINC70704643 | Non-Toxic | Non-Carcinogens | | Non-Inhibitor | III/0.6138 | 2.5257 |
|  | ZINC70704648 | Non-Toxic | Non-Carcinogens | | Non-Inhibitor | III/0.6138 | 2.5257 |
|  | ZINC70704650 | Non-Toxic | Non-Carcinogens | | Non-Inhibitor | III/0.6480 | 2.3200 |
|  | ZINC70704667 | Non-Toxic | Non-Carcinogens | | Non-Inhibitor | III/0.6511 | 2.4559 |
|  | ZINC70704687 | Non-Toxic | Non-Carcinogens | | Non-Inhibitor | III/0.6889 | 2.2732 |
|  | ZINC70704696 | Non-Toxic | Non-Carcinogens | | Inhibitor | III/0.5128 | 2.4705 |
|  | ZINC70704741 | Non-Toxic | Non-Carcinogens | | Inhibitor | III/0.6392 | 2.2367 |
|  | ZINC70704777 | Non-Toxic | Non-Carcinogens | | Non-Inhibitor | III/0.6459 | 2.3756 |
|  | ZINC70704782 | Non-Toxic | Non-Carcinogens | | Inhibitor | III/0.6565 | 2.2504 |
|  | ZINC70704817 | Non-Toxic | Non-Carcinogens | | Non-Inhibitor | III/0.6247 | 2.3781 |
|  | ZINC70704820 | Non-Toxic | Non-Carcinogens | | Non-Inhibitor | III/0.7006 | 2.3781 |
|  | ZINC70704831 | Non-Toxic | Non-Carcinogens | | Non-Inhibitor | III/0.6960 | 2.2792 |
|  | ZINC70704924 | Non-Toxic | Non-Carcinogens | | Non-Inhibitor | III/0.6201 | 2.3188 |
|  | ZINC70704940 | Non-Toxic | Non-Carcinogens | | Non-Inhibitor | III/0.6326 | 2.5011 |
|  | ZINC70704954 | Non-Toxic | Non-Carcinogens | | Inhibitor | III/0.5722 | 2.3462 |
|  | ZINC70704963 | Non-Toxic | Non-Carcinogens | | Inhibitor | III/0.5722 | 2.3462 |
|  | ZINC70704967 | Non-Toxic | Non-Carcinogens | | Inhibitor | III/0.5981 | 2.3921 |
|  | ZINC70704970 | Non-Toxic | Non-Carcinogens | | Inhibitor | III/0.6515 | 2.4541 |
|  | **ZINC70704976** | Non-Toxic | Non-Carcinogens | | Non-Inhibitor | III/0.6120 | 2.2111 |
|  | ZINC70704983 | Non-Toxic | Non-Carcinogens | | Inhibitor | III/0.6897 | 2.1847 |
|  | ZINC70705014 | Non-Toxic | Non-Carcinogens | | Non-Inhibitor | III/0.6183 | 2.5273 |
|  | ZINC70705018 | Non-Toxic | Non-Carcinogens | | Non-Inhibitor | III/0.6183 | 2.5273 |
|  | ZINC70705022 | Non-Toxic | Non-Carcinogens | | Non-Inhibitor | III/0.6459 | 2.3756 |
|  | ZINC70705055 | Non-Toxic | Non-Carcinogens | | Inhibitor | III/0.6952 | 2.2386 |
|  | ZINC70705072 | Non-Toxic | Non-Carcinogens | | Non-Inhibitor | III/0.5954 | 2.3786 |
|  | ZINC70705084 | Non-Toxic | Non-Carcinogens | | Non-Inhibitor | III/0.5954 | 2.3786 |
|  | ZINC70705092 | Non-Toxic | Non-Carcinogens | | Inhibitor | III/0.6705 | 2.5444 |
|  | ZINC70705102 | Non-Toxic | Non-Carcinogens | | Non-Inhibitor | III/0.6751 | 2.3157 |
|  | ZINC70705211 | Non-Toxic | Non-Carcinogens | | Non-Inhibitor | III/0.7100 | 2.0902 |
|  | ZINC70705227 | Non-Toxic | Non-Carcinogens | | Non-Inhibitor | III/0.6195 | 2.1989 |
|  | ZINC70705243 | Non-Toxic | Non-Carcinogens | | Inhibitor | III/0.6693 | 2.4716 |
|  | ZINC70705246 | Non-Toxic | Non-Carcinogens | | Inhibitor | III/0.6693 | 2.4716 |
|  | ZINC70705249 | Non-Toxic | Non-Carcinogens | | Inhibitor | III/0.6693 | 2.4716 |
|  | ZINC70705273 | Non-Toxic | Non-Carcinogens | | Inhibitor | III/0.6949 | 2.2117 |
|  | ZINC70705332 | Non-Toxic | Non-Carcinogens | | Non-Inhibitor | III/0.6434 | 2.2814 |
|  | ZINC70705335 | Non-Toxic | Non-Carcinogens | | Non-Inhibitor | III/0.6434 | 2.2814 |
|  | ZINC70705341 | Non-Toxic | Non-Carcinogens | | Inhibitor | III/0.6949 | 2.2117 |
|  | ZINC70705347 | Non-Toxic | Non-Carcinogens | | Non-Inhibitor | III/0.6512 | 2.3320 |
|  | ZINC70705373 | Non-Toxic | Non-Carcinogens | | Inhibitor | III/0.6365 | 2.3341 |
|  | ZINC70705454 | Non-Toxic | Non-Carcinogens | | Non-Inhibitor | III/0.6235 | 2.2855 |
|  | ZINC70705469 | Non-Toxic | Non-Carcinogens | | Non-Inhibitor | III/0.6276 | 2.3806 |
|  | ZINC70705472 | Non-Toxic | Non-Carcinogens | | Non-Inhibitor | III/0.6276 | 2.3806 |
|  | ZINC70705481 | Non-Toxic | Non-Carcinogens | | Non-Inhibitor | III/0.6459 | 2.3756 |
|  | ZINC70705530 | Non-Toxic | Non-Carcinogens | | Inhibitor | III/0.6284 | 2.3283 |
|  | **ZINC70705576** | Non-Toxic | Non-Carcinogens | | Non-Inhibitor | III/0.6174 | 2.3102 |
|  | ZINC70705586 | Non-Toxic | Non-Carcinogens | | Inhibitor | III/0.5738 | 2.3486 |
|  | ZINC70705594 | Non-Toxic | Non-Carcinogens | | Inhibitor | III/0.7044 | 2.2647 |
|  | ZINC70705647 | Non-Toxic | Non-Carcinogens | | Non-Inhibitor | III/0.6668 | 2.2499 |
|  | ZINC70705650 | Non-Toxic | Non-Carcinogens | | Non-Inhibitor | III/0.6291 | 2.1558 |
|  | ZINC70705678 | Non-Toxic | Non-Carcinogens | | Inhibitor | III/0.6767 | 2.4111 |
|  | ZINC70705686 | Non-Toxic | Non-Carcinogens | | Inhibitor | III/0.6949 | 2.2117 |
|  | ZINC70705694 | Non-Toxic | Non-Carcinogens | | Non-Inhibitor | III/0.6158 | 2.3261 |
|  | ZINC70705713 | Non-Toxic | Non-Carcinogens | | Inhibitor | III/0.7044 | 2.2647 |
|  | ZINC70705731 | Non-Toxic | Non-Carcinogens | | Non-Inhibitor | III/0.6183 | 2.5273 |
|  | ZINC70705733 | Non-Toxic | Non-Carcinogens | | Non-Inhibitor | III/0.6183 | 2.5273 |
|  | ZINC70705741 | Non-Toxic | Non-Carcinogens | | Non-Inhibitor | III/0.9632 | 2.0902 |
|  | ZINC70705766 | Non-Toxic | Non-Carcinogens | | Non-Inhibitor | III/0.6434 | 2.2814 |
|  | ZINC70705771 | Non-Toxic | Non-Carcinogens | | Non-Inhibitor | III/0.6434 | 2.2814 |
|  | ZINC70705789 | Non-Toxic | Non-Carcinogens | | Non-Inhibitor | III/0.6474 | 2.3082 |
|  | ZINC70705793 | Non-Toxic | Non-Carcinogens | | Non-Inhibitor | III/0.6474 | 2.3082 |
|  | ZINC70705797 | Non-Toxic | Non-Carcinogens | | Non-Inhibitor | III/0.6955 | 2.3543 |
|  | ZINC70705801 | Non-Toxic | Non-Carcinogens | | Inhibitor | III/0.6365 | 2.3341 |
|  | ZINC70705817 | Non-Toxic | Non-Carcinogens | | Non-Inhibitor | III/0.7270 | 2.0902 |
|  | ZINC70705820 | Non-Toxic | Non-Carcinogens | | Non-Inhibitor | III/0.6107 | 2.1740 |
|  | ZINC70705851 | Non-Toxic | Non-Carcinogens | | Non-Inhibitor | III/0.6384 | 2.2940 |
|  | ZINC70705862 | Non-Toxic | Non-Carcinogens | | Inhibitor | III/0.5487 | 2.0507 |
|  | ZINC70705876 | Non-Toxic | Non-Carcinogens | | Non-Inhibitor | III/0.6659 | 2.2426 |
|  | ZINC70705882 | Non-Toxic | Non-Carcinogens | | Non-Inhibitor | III/0.6110 | 2.4618 |
|  | ZINC70705913 | Non-Toxic | Non-Carcinogens | | Non-Inhibitor | III/0.6433 | 2.4516 |
|  | ZINC70705940 | Non-Toxic | Non-Carcinogens | | Inhibitor | III/0.6923 | 2.5687 |
|  | ZINC70705965 | Non-Toxic | Non-Carcinogens | | Non-Inhibitor | III/0.6382 | 2.4241 |
|  | ZINC70705983 | Non-Toxic | Non-Carcinogens | | Non-Inhibitor | III/0.6519 | 2.3235 |
|  | ZINC70706001 | Non-Toxic | Non-Carcinogens | | Inhibitor | III/0.6588 | 2.4387 |
|  | ZINC70706036 | Non-Toxic | Non-Carcinogens | | Inhibitor | III/0.6550 | 2.3435 |
|  | **ZINC70706110** | Non-Toxic | Non-Carcinogens | | Non-Inhibitor | III/0.4134 | 2.2305 |
|  | **ZINC70706152** | Non-Toxic | Non-Carcinogens | | Non-Inhibitor | III/0.5890 | 2.2318 |
|  | ZINC70706199 | Non-Toxic | Non-Carcinogens | | Non-Inhibitor | III/0.6399 | 2.3050 |
|  | ZINC70706205 | Non-Toxic | Non-Carcinogens | | Inhibitor | III/0.6952 | 2.2386 |
|  | ZINC70706216 | Non-Toxic | Non-Carcinogens | | Inhibitor | III/0.6952 | 2.2386 |
|  | ZINC70706255 | Non-Toxic | Non-Carcinogens | | Inhibitor | III/0.6489 | 2.5040 |
|  | ZINC70706259 | Non-Toxic | Non-Carcinogens | | Inhibitor | III/0.6489 | 2.5040 |
|  | ZINC70706263 | Non-Toxic | Non-Carcinogens | | Inhibitor | III/0.6489 | 2.5040 |
|  | ZINC70706269 | Non-Toxic | Non-Carcinogens | | Non-Inhibitor | III/0.6326 | 2.5011 |
|  | ZINC70706273 | Non-Toxic | Non-Carcinogens | | Non-Inhibitor | III/0.6751 | 2.3157 |
|  | ZINC70706301 | Non-Toxic | | Non-Carcinogens | Inhibitor | III/0.6949 | 2.2117 |
|  | ZINC70706313 | Non-Toxic | | Non-Carcinogens | Non-Inhibitor | III/0.6562 | 2.1777 |
|  | ZINC70706346 | Non-Toxic | | Non-Carcinogens | Non-Inhibitor | III/0.7164 | 2.0365 |
|  | ZINC70706388 | Non-Toxic | | Non-Carcinogens | Inhibitor | III/0.7253 | 2.2461 |
|  | ZINC70706410 | Non-Toxic | | Non-Carcinogens | Non-Inhibitor | III/0.7164 | 2.0365 |
|  | ZINC70706450 | Non-Toxic | | Non-Carcinogens | Inhibitor | III/0.5953 | 2.2453 |
|  | ZINC70706454 | Non-Toxic | | Non-Carcinogens | Inhibitor | III/0.7158 | 2.1302 |
|  | ZINC70706476 | Non-Toxic | | Non-Carcinogens | Non-Inhibitor | III/0.6083 | 2.3356 |
|  | ZINC70706482 | Non-Toxic | | Non-Carcinogens | Non-Inhibitor | III/0.6083 | 2.3356 |
|  | ZINC70706497 | Non-Toxic | | Non-Carcinogens | Non-Inhibitor | III/0.6511 | 2.4559 |
|  | ZINC70706500 | Non-Toxic | | Non-Carcinogens | Inhibitor | III/0.6599 | 2.4522 |
|  | ZINC70706505 | Non-Toxic | | Non-Carcinogens | Inhibitor | III/0.6599 | 2.4522 |
|  | ZINC70706527 | Non-Toxic | | Non-Carcinogens | Inhibitor | III/0.6284 | 2.3283 |
|  | ZINC70706554 | Non-Toxic | | Non-Carcinogens | Inhibitor | III/0.6723 | 2.6447 |
|  | ZINC70706561 | Non-Toxic | | Non-Carcinogens | Inhibitor | III/0.6723 | 2.6447 |
|  | ZINC70706606 | Non-Toxic | | Non-Carcinogens | Non-Inhibitor | III/0.6110 | 2.4618 |
|  | ZINC70706646 | Non-Toxic | | Non-Carcinogens | Non-Inhibitor | III/0.6083 | 2.3356 |
|  | ZINC70706651 | Non-Toxic | | Non-Carcinogens | Non-Inhibitor | III/0.6083 | 2.3356 |
|  | ZINC70706660 | Non-Toxic | | Non-Carcinogens | Inhibitor | III/0.5995 | 2.4070 |
|  | ZINC70706670 | Non-Toxic | | Non-Carcinogens | Non-Inhibitor | III/0.6960 | 2.2792 |
|  | ZINC70706672 | Non-Toxic | | Non-Carcinogens | Non-Inhibitor | III/0.6960 | 2.2792 |
|  | ZINC70706692 | Non-Toxic | | Non-Carcinogens | Non-Inhibitor | III/0.6387 | 2.5224 |
|  | ZINC70706700 | Non-Toxic | | Non-Carcinogens | Non-Inhibitor | III/0.6270 | 2.4727 |
|  | ZINC70706714 | Non-Toxic | | Non-Carcinogens | Inhibitor | III/0.5936 | 2.3763 |
|  | ZINC70706747 | Non-Toxic | | Non-Carcinogens | Non-Inhibitor | III/0.6386 | 2.3454 |
|  | ZINC70706767 | Non-Toxic | | Non-Carcinogens | Non-Inhibitor | III/0.6512 | 2.3320 |
|  | ZINC70706775 | Non-Toxic | | Non-Carcinogens | Non-Inhibitor | III/0.5720 | 2.3205 |
|  | ZINC70706779 | Non-Toxic | | Non-Carcinogens | Non-Inhibitor | III/0.5720 | 2.3205 |
|  | ZINC70706783 | Non-Toxic | | Non-Carcinogens | Non-Inhibitor | III/0.6235 | 2.2855 |
|  | ZINC70706791 | Non-Toxic | | Non-Carcinogens | Non-Inhibitor | III/0.7166 | 2.1402 |
|  | ZINC70706801 | Non-Toxic | | Non-Carcinogens | Non-Inhibitor | III/0.6110 | 2.4618 |
|  | ZINC70706824 | Non-Toxic | | Non-Carcinogens | Inhibitor | III/0.7113 | 2.1064 |
|  | ZINC70706830 | Non-Toxic | | Non-Carcinogens | Non-Inhibitor | III/0.6198 | 2.3568 |
|  | ZINC70706862 | Non-Toxic | | Non-Carcinogens | Inhibitor | III/0.6757 | 2.7459 |
|  | ZINC70706868 | Non-Toxic | | Non-Carcinogens | Non-Inhibitor | III/0.6668 | 2.2499 |
|  | ZINC70706920 | Non-Toxic | | Non-Carcinogens | Non-Inhibitor | III/0.6889 | 2.2732 |
|  | ZINC70706955 | Non-Toxic | | Non-Carcinogens | Non-Inhibitor | III/0.6751 | 2.3157 |
|  | ZINC70706967 | Non-Toxic | | Non-Carcinogens | Non-Inhibitor | III/0.6258 | 2.2665 |
|  | **ZINC70706981** | Non-Toxic | | Non-Carcinogens | Non-Inhibitor | III/0.5886 | 2.5096 |
|  | **ZINC70706982** | Non-Toxic | | Non-Carcinogens | Non-Inhibitor | III/0.5886 | 2.5096 |
|  | ZINC70706983 | Non-Toxic | | Non-Carcinogens | Inhibitor | III/0.6710 | 2.3584 |
|  | ZINC70707015 | Non-Toxic | | Non-Carcinogens | Non-Inhibitor | III/0.6559 | 2.0310 |
|  | ZINC70707063 | Non-Toxic | | Non-Carcinogens | Non-Inhibitor | III/0.6386 | 2.3454 |
|  | ZINC70707070 | Non-Toxic | | Non-Carcinogens | Inhibitor | III/0.6655 | 2.1700 |
|  | ZINC70707076 | Non-Toxic | | Non-Carcinogens | Inhibitor | III/0.6655 | 2.1700 |
|  | ZINC70707086 | Non-Toxic | | Non-Carcinogens | Inhibitor | III/0.6949 | 2.2117 |
|  | ZINC70707113 | Non-Toxic | | Non-Carcinogens | Non-Inhibitor | III/0.6474 | 2.3082 |
|  | ZINC70707115 | Non-Toxic | | Non-Carcinogens | Non-Inhibitor | III/0.6474 | 2.3082 |
|  | **ZINC70707119** | Non-Toxic | | Non-Carcinogens | Non-Inhibitor | III/0.6355 | 2.2023 |
|  | ZINC70707131 | Non-Toxic | | Non-Carcinogens | Inhibitor | III/0.6333 | 2.5402 |
|  | ZINC70707132 | Non-Toxic | | Non-Carcinogens | Inhibitor | III/0.6333 | 2.5402 |
|  | **ZINC70707134** | Non-Toxic | | Non-Carcinogens | Non-Inhibitor | III/0.5890 | 2.2318 |
|  | ZINC70707151 | Non-Toxic | | Non-Carcinogens | Non-Inhibitor | III/0.6326 | 2.5011 |
|  | ZINC70707156 | Non-Toxic | | Non-Carcinogens | Non-Inhibitor | III/0.7110 | 1.9651 |
|  | ZINC70707164 | Non-Toxic | | Non-Carcinogens | Inhibitor | III/0.7044 | 2.2647 |
|  | ZINC70707172 | Non-Toxic | | Non-Carcinogens | Non-Inhibitor | III/0.6757 | 2.3251 |
|  | ZINC70707186 | Non-Toxic | | Non-Carcinogens | Inhibitor | III/0.7253 | 2.2461 |
|  | ZINC70707192 | Non-Toxic | | Non-Carcinogens | Non-Inhibitor | III/0.6251 | 2.4709 |
|  | ZINC70707205 | Non-Toxic | | Non-Carcinogens | Non-Inhibitor | III/0.6459 | 2.3756 |
|  | ZINC70707209 | Non-Toxic | | Non-Carcinogens | Inhibitor | III/0.6897 | 2.1847 |
|  | ZINC70707221 | Non-Toxic | | Non-Carcinogens | Non-Inhibitor | III/0.6110 | 2.4618 |
|  | ZINC70707222 | Non-Toxic | | Non-Carcinogens | Inhibitor | III/0.6844 | 2.3122 |
|  | ZINC70707229 | Non-Toxic | | Non-Carcinogens | Inhibitor | III/0.6718 | 2.4139 |
|  | ZINC70707251 | Non-Toxic | | Non-Carcinogens | Inhibitor | III/0.6767 | 2.4111 |
|  | ZINC70707260 | Non-Toxic | | Non-Carcinogens | Inhibitor | III/0.5941 | 2.6479 |
|  | ZINC70707277 | Non-Toxic | | Non-Carcinogens | Non-Inhibitor | III/0.6459 | 2.3756 |
|  | ZINC70707332 | Non-Toxic | | Non-Carcinogens | Non-Inhibitor | III/0.6158 | 2.3261 |
|  | ZINC70707349 | Non-Toxic | | Non-Carcinogens | Non-Inhibitor | III/0.6511 | 2.4559 |
|  | ZINC70707371 | Non-Toxic | | Non-Carcinogens | Inhibitor | III/0.6655 | 2.1700 |
|  | ZINC70707376 | Non-Toxic | | Non-Carcinogens | Inhibitor | III/0.6655 | 2.1700 |
|  | ZINC70707395 | Non-Toxic | | Non-Carcinogens | Non-Inhibitor | III/0.6930 | 2.5254 |
|  | ZINC70707416 | Non-Toxic | | Non-Carcinogens | Inhibitor | III/0.6151 | 2.5148 |
|  | ZINC70707422 | Non-Toxic | | Non-Carcinogens | Inhibitor | III/0.6820 | 2.3698 |
|  | ZINC70707443 | Non-Toxic | | Non-Carcinogens | Inhibitor | III/0.6453 | 2.2969 |
|  | ZINC70707449 | Non-Toxic | | Non-Carcinogens | Inhibitor | III/0.6952 | 2.2386 |
|  | ZINC70707458 | Non-Toxic | | Non-Carcinogens | Inhibitor | III/0.7113 | 2.1064 |
|  | ZINC70707552 | Non-Toxic | | Non-Carcinogens | Non-Inhibitor | III/0.6519 | 2.3235 |
|  | ZINC70707580 | Non-Toxic | | Non-Carcinogens | Non-Inhibitor | III/0.6559 | 2.0310 |
|  | ZINC70707597 | Non-Toxic | | Non-Carcinogens | Inhibitor | III/0.7253 | 2.2461 |
|  | ZINC70707603 | Non-Toxic | | Non-Carcinogens | Non-Inhibitor | III/0.7164 | 2.0365 |
|  | ZINC70707609 | Non-Toxic | | Non-Carcinogens | Non-Inhibitor | III/0.6326 | 2.5011 |
|  | ZINC70707624 | Non-Toxic | | Non-Carcinogens | Non-Inhibitor | III/0.6476 | 2.3082 |
|  | ZINC70707626 | Non-Toxic | | Non-Carcinogens | Non-Inhibitor | III/0.6476 | 2.3082 |
|  | **ZINC70707655** | Non-Toxic | | Non-Carcinogens | Non-Inhibitor | III/0.6016 | 2.2945 |
|  | ZINC70707671 | Non-Toxic | | Non-Carcinogens | Inhibitor | III/0.6948 | 2.3469 |
|  | ZINC70707750 | Non-Toxic | | Non-Carcinogens | Inhibitor | III/0.7158 | 2.1302 |
|  | ZINC70707762 | Non-Toxic | | Non-Carcinogens | Non-Inhibitor | III/0.5954 | 2.3786 |
|  | ZINC70707772 | Non-Toxic | | Non-Carcinogens | Non-Inhibitor | III/0.5954 | 2.3786 |
|  | ZINC70712128 | Non-Toxic | | Non-Carcinogens | Non-Inhibitor | III/0.6231 | 2.3269 |
|  | ZINC71382583 | Non-Toxic | | Non-Carcinogens | Non-Inhibitor | III/0.5658 | 3.0525 |
|  | ZINC77269479 | Toxic | | Non-Carcinogens | Non-Inhibitor | III/0.3483 | 3.2895 |
|  | ZINC79209918 | Non-Toxic | | Non-Carcinogens | Non-Inhibitor | III/0.6891 | 3.0120 |
|  | ZINC79212807 | Non-Toxic | | Non-Carcinogens | Non-Inhibitor | III/0.5631 | 2.5678 |
|  | ZINC82185013 | Toxic | | Non-Carcinogens | Non-Inhibitor | III/0.6095 | 2.5837 |
|  | ZINC85536937 | Non-Toxic | | Non-Carcinogens | Non-Inhibitor | III/0.5915 | 2.5973 |
|  | ZINC85552021 | Non-Toxic | | Non-Carcinogens | Non-Inhibitor | III/0.5725 | 2.4517 |
|  | ZINC85866826 | Non-Toxic | | Non-Carcinogens | Non-Inhibitor | III/0.5884 | 2.6096 |
|  | ZINC85867137 | Non-Toxic | | Non-Carcinogens | Non-Inhibitor | III/0.6742 | 2.1653 |
|  | ZINC85902344 | Non-Toxic | | Non-Carcinogens | Non-Inhibitor | III/0.5622 | 2.6375 |
|  | **ZINC95100194** | Non-Toxic | | Non-Carcinogens | Non-Inhibitor | III/0.7202 | 2.0775 |
|  | ZINC95100209 | Toxic | | Non-Carcinogens | Non-Inhibitor | III/0.5984 | 2.3563 |
|  | ZINC95100330 | Non-Toxic | | Non-Carcinogens | Inhibitor | III/0.5955 | 2.5193 |
|  | ZINC95100337 | Non-Toxic | | Non-Carcinogens | Non-Inhibitor | III/0.4568 | 2.5621 |
|  | ZINC95100338 | Non-Toxic | | Non-Carcinogens | Non-Inhibitor | III/0.5647 | 2.5025 |
|  | ZINC95100378 | Toxic | | Non-Carcinogens | Non-Inhibitor | III/0.6223 | 2.5694 |
|  | ZINC95100379 | Toxic | | Non-Carcinogens | Non-Inhibitor | III/0.6386 | 2.5518 |
|  | ZINC95100380 | Non-Toxic | | Non-Carcinogens | Non-Inhibitor | III/0.5485 | 2.6615 |
|  | ZINC95100381 | Toxic | | Non-Carcinogens | Non-Inhibitor | III/0.6695 | 2.5038 |
|  | ZINC95100382 | Toxic | | Non-Carcinogens | Non-Inhibitor | III/0.6338 | 2.6109 |
|  | ZINC95100383 | Non-Toxic | | Non-Carcinogens | Non-Inhibitor | III/0.5727 | 2.5752 |
|  | ZINC95101040 | Toxic | | Non-Carcinogens | Non-Inhibitor | III/0.6095 | 2.5837 |
|  | ZINC95101062 | Non-Toxic | | Non-Carcinogens | Inhibitor | III/0.6014 | 2.5097 |
|  | ZINC95101078 | Non-Toxic | | Non-Carcinogens | Inhibitor | III/0.5670 | 2.2964 |
|  | ZINC95612014 | Non-Toxic | | Non-Carcinogens | Non-Inhibitor | III/0.6964 | 2.3928 |
|  | ZINC96316264 | Non-Toxic | | Non-Carcinogens | Non-Inhibitor | III/0.4244 | 3.5418 |

**Supplementary Table S4.** Summary of binding affinity with interacting residues of the top 20 compounds with control compound ANP obtained from molecular docking studies by three docking tools: Autodock Tools, AutodockVina and Molegro Virtual Docker. The Residues which involved in hydrogen bonding were highlighted in bold as well as selected hits for MDS are also highlighted in bold.

| **Sr. No.** | **Compound ID** | **IC_50_** | **Binding Energy** | **No. of H bond** | **Residues** | **Binding energy**  **Vina** | **No. of H bond** | **Residues** | **MVD**  **Score** | **No. of H bond** | **Residues** |
| --- | --- | --- | --- | --- | --- | --- | --- | --- | --- | --- | --- |
|  | ANP | 1.83  uM | -7.83 | 8 | **Ile62**,**Asn64**,Val70,Ala83,Leu132,Tyr134,Val110,**Asp133**,Val135,Leu188,**Thr138**,Tyr140,**Arg141**,Gln185 | -7.8 | 8 | **Ser66**,**Phe67**,**Gly68**,Val70,**Val135**,Asp200,Tyr134,Ala83,Leu132,**Lys85**,Arg141,**Asp133** | -179.077 | 9 | Gly63,Ser66,Gly65,**Phe67**,**Cys199**,Asp200,Lys85,Leu188,Val70,Gly68,**Ile62**,**Tyr134**,Pro136,**Arg141**,Thr138,Asn186 |
|  | **ZINC_15968620** | 3.7 nM | -11.50 | 5 | Ile62,Thr134,**Val135**,Asn64,Ala83,Leu132,Leu188,Cys199,Asp200,Asn186,**Gln185**,Thr138,**Arg141** | -10.6 | 3 | Gly63,**Asn64**,Gly65,Ile62,Val135,Tyr134,Val70,Ala83,**Lys85**,Gly68,Phe67,Asp200,Leu188,**Lys183**,Gln185,Thr138,Arg141 | -184.884 | 3 | Phe67,Ser66,Gly65,**Lys85**,Leu132,Gly68,Val87,**Asn64**,Val70,Gly63,Ala83,Ile62,Tyr134,Val135,Arg141,Thr138,Leu188,Val110,Cys199,Asp200,Asn186,**Lys183**,Gln185,Tyr140, |
|  | **ZINC_15968622** | 3.52nM | -11.53 | 4 | Ile62,Tyr134,Ala83,Leu132,**Val135**,Asn64,Leu188,Cys199,Asn186,**Gln185**,Tyr140,**Thr138,Arg141** | -10.5 | 2 | Gly65,**Asn64**,Val35,Gly63,Val70,Ile62,Tyr134,Ala83,**Lys85**,Gly68,Phe67,Asp200,Lys183,Gln185,Leu188,Thr138,Arg141 | -188.439 | 2 | Phe67,Ser66,Asp200,Cys199,Gly65,Val110,Gly68,**Lys85**,Val87,**Asn64**,Leu132,Val70,Val135,Gly63,Ala83,Ile62,Tyr134,Leu188,Asn186,Lys183,Gln185,Thr138,Arg141,Tyr140 |
|  | ZINC_65731330 | 231.09  nM | -9.05 | 7 | Asn64,**Ser66**,Ala83,Val135,Leu188,Asp200,**Asn186**,Asp181,**Lys183**,Gln185,**Thr138**,Tyr140,**Arg141** | -9.0 | 3 | Ser66,**Phe67**,Asp200,Gly65,Gly68,Asn64,**Lys85**,Val110,Gly63,Val70,Leu132,Val135,Ile62,Ala83,Tyr134,Leu188,Arg141,Thr138,Asn186,Gln185,**Lys183**,Asp181 | -185.888 | 7 | Gly63,Asn64,Val110,Val70,Lys85,Leu132,Ile62,Ala83,Tyr134,Val135,Leu188,Cys199,Asp200,Arg141,Thr138,Asn186,Gln185,Tyr140,Arg144 |
|  | ZINC_70699156 | 12.33  nM | -10.79 | 3 | Ile62,Val135,**Asn64**,Val70,Ala83,**Lys85**,Gly68,Phe67,Asp200,**Lys183**,Gln185,Thr138,Arg141 | -10.3 | 3 | **Asn64**,Gly65,Phe67,Gly68,**Lys85**,Asp200,Val135,Val63,Val70,Ala83,Ile62,Tyr134,Leu188,Thr138,Arg141,Tyr140,Gln185,**Lys183** | -188.305 | 3 | Gly65,Val110,**Asn64**,Gly68,**Lys85**,Val87,Leu132,Val70,Gly63,Val135,Ala83,Ile62,Tyr134,Leu188,Cys199,Asp200,Ser66,Phe67,Asn186,**Lys183**,Gln185,Tyr140,Thr138,Arg141 |
|  | ZINC_70699739 | 4.32  nM | -11.41 | 4 | Asn64,**Val135**,Val70,Tyr134,Ala83,Leu132,Lys85,Cys199,Asp200,Asn186,Lys183,**Gln185**,Leu188,Thr138,**Arg141** | -10.3 | 3 | **Asn64**,Gly65,Phe67,Gly68,**Lys85**,Asp200,Gly63,Val135,Val70,Ile62,Ala83,Tyr134,Leu188,Thr138,Arg141,Gln185,**Lys183** | -183.562 | 4 | Gly65,Val110,Asn64,Gly68,**Lys85**,Val87,Leu132,Val70,Gly63,Val135,Ala83,Ile62,Tyr134,Leu188,**Cys199**,**Asp200**,Phe67,Asn186,Lys183,Gln185,Thr138,Arg141 |
|  | ZINC_70700165 | 3.27 nM | -11.58 | 6 | Ile62,**Val135**,Tyr134,Ala83,Leu132,Val70,Leu188,**Asn64**,Cys199,Asn186,Lys183,**Gln185**,Tyr140,**Thr138,Arg141** | -10.0 | 2 | **Asn64**,Gly65,Phe67,Val110,Asp200,Gly68,**Lys85**,Val135,Gly63,Val70,Ile62,Ala83,Tyr134,Leu188,Thr138,Arg141,Gln185 | -181.252 | 6 | **Asn64**,Val110,**Lys85**,Leu132,Val70,Gly63,**Val135**,Ala83,Ile62,Tyr134,Pro136,Arg141,Thr138,Leu188,**Cys199**,**Asp200**,Asn186,Gln185,Lys183 |
|  | ZINC_70700623 | 17.84  nM | -10.57 | 2 | Asn64,Val135,Ile62,Val70,Ala83,**Lys85**,Gly68,Phe67,Asp200,Asn186,**Lys183**,Gln185,Thr138,Arg141 | -10.2 | 2 | **Asn64**,Gly65,Phe67,Asp200,Gly68,**Lys85**,Val135,Gly63,Val70,Ala83,Ile62,Tyr134,Leu188,Arg141,Thr138,Tyr140,Gln185,Lys183 | -191.691 | 3 | Gly65,Ser66,Phe67,**Lys85**,Leu132,Gly68,Val87,**Asn64**,Val70,Gly63,Ala83,Ile62,Tyr134,Val135,Val110,Leu188,Asp200,Cys199,Gln185,Lys183,Tyr140,Thr138,**Arg141** |
|  | ZINC_70700682 | 11.6  nM | -10.83 | 3 | **Asn64**,**Val135**,Ile62,Val70,Tyr134,Ala83,Leu132,Leu188,Cys199,Gln185,Lys183,Tyr140,Thr138,**Arg141** | -10.2 | 2 | **Asn64**,Gly65,Phe67,Gly68,Asp200,**Lys85**,Val70,Gly63,Val135,Ile62,Ala83,Tyr134,Leu188,Thr138,Arg141,Gln185,Lys183 | -181.853 | 2 | **Asn64**,Gly65,Ser67,Ser66,Asp200,Cys199,Val110,Gly63,**Lys85**,Gly68,Val87,Val70,Leu132,Ile62,Ala83,Tyr134,Val135,Leu188,Arg141,Thr138,Gln185,Lys183 |
|  | ZINC_70700931 | 14.52  nM | -10.69 | 4 | **Asn64**,Gly68,**Lys85**,Val70,Val135,Ile62,Ala83,Phe67,Asp200,Asn186,**Lys183**,Gln185,Thr138,**Arg141** | -10.00 | 2 | **Asn64**,Gly65,Phe67,Gly68,Asp200,**Lys85**,Val135,Gly63,Val70,Ile62,Ala83,Tyr134,Leu188,Thr138,Arg141,Gln185,Lys183 | -188.694 | 3 | Gly65,Val110,**Asn64**,Gly68,**Lys85**,Val87,Leu132,Val70,Gly63,Val135,Ala83,Ile62,Tyr134,Leu188,Thr138,**Arg141**,Tyr140,Gln185,Lys183,Asn186,Asp200,Cys199,Ser66,Phe67 |
|  | ZINC_70701019 | 10.32  nM | -10.9 | 4 | **Asn64**,Phe67,Gly68,**Lys85**,Asp200,Val70,Ala83,Ile62,Val135,**Arg141**,Thr138,Gln185,**Lys183** | -10.1 | 3 | **Asn64**,Gly65,Phe67,Gly68,Val110,Asp200,**Lys85**,Val70,Gly63,Val135,Ile62,Ala83,Tyr134,Leu188,Thr138,Arg141,Gln185,**Lys183** | -189.597 | 4 | Asn64,Gly65,Val110,Gly68,**Lys85**,Val87,Leu132,Val70,Gly63,Val135,Ile62,Ala83,Tyr134,Leu188,Thr138,Arg141,Gln185,Asn186,Lys183,**Asp200,Cys199**,Phe67 |
|  | **ZINC_70704976** | 4.2  nM | -11.43 | 4 | Asn64,**Val135**,Leu132,Ala83,Ile62,Tyr134,Leu188,**Thr138,Arg141**,Tyr140,Gln185,Asn186,Cys199,Asp200 | -10.00 | 2 | **Asn64**,Gly65,Gly68,**Lys85**,Gly63,Val135,Val70,Ile62,Tyr134,Ala83,Phe67,Ser66,Asp200,Lys183,Gln185,Leu188,Thr138,Arg141 | -207.046 | 3 | **Asn64**,Gly65,Ser66,Phe67,Gly68,Val70,**Lys85**,Val87,Asp200,Ile62,Cys199,Leu132,Ala83,Val110,Tyr134,Val135,Leu188,Thr138,Arg141Gln185,**Lys183**,Tyr140 |
|  | ZINC_70705576 | 6.85  nM | -11.14 | 6 | Ile62,**Asn64**,Val70,**Val135**,Ala83,Tyr134,Leu132,Cys199,Leu188,**Gln185**,Tyr140,Thr138,**Arg141** | -9.8 | 2 | Ile62,Val70,**Lys85**,Gly63,**Asn64,**Gly68,Gly65,Phe67,Ser66,Ala83,Tyr134,Val135,Val110,Leu110,Leu188,Thr138,Arg141,Asp200,Gln185,Lys183 | -195.222 | 3 | Ile62,Ala83,Gly63,Val70,Leu132,**Asn64**,**Lys85**,Gly65,Gly68,Val87,Ser66,Phe67,Asp200,**Lys183**,Asn186,Gln185,Cys199,Val110,Leu188,Thr138,Arg141,Val135,Tyr134 |
|  | ZINC_70706110 | 10.37  nM | -10.89 | 3 | Val64,Val70,**Val135**,Ile62,Tyr134,Ala83,Leu132,**Lys85**,Val110,Cys199,**Asp200**,Asn186,Leu188,Arg141 | -9.7 | 2 | **Asn64**,Val135,Gly63,Ile62,Tyr134,Val70,Ala83,Leu132,**Lys85**,Gly68,Val110,Gly65,Phe67,Asp200,Lys183,Gln185,:eu188,Thr138,Arg141 | -186.661 | 2 | Ile62,Val110,Ala83,Leu132,Val70,**Lys85**,Val87,Gly63,Cys199,Gly68,Asp200,**Asn64**,Gly65,Phe67,Ser66,Lys183,Gln185,Arg141,Thr138,Leu188,Val135,Tyr134,Ala83,Leu132 |
|  | ZINC_70706152 | 1.94  nM | -11.89 | 3 | Ile62,**Asn64,Val135**,Tyr134,Ala83,Leu132,Leu188,Asn186,**Thr138**,Gln185,Tyr140,Arg144,Arg141 | -10.3 | 1 | Asn64,Gly65,Val110,Gly68,Lys85,gly63,Val70,Leu132,Ala83,Phe67,Asp200,Cys199,Asn186,Lys183,**Gln185**,Tyr140,Leu188,Thr138,Arg141 | -193.105 | 3 | Asn64,Val110,Gly65,**Cys199**,Asp200,Phe201,Ser66,Phe67,Glu97,Met101,Gly68,**Lys85**,Leu132,Val70,Gly63,Ala83,Ile62,Tyr134,Val135,Leu188,Asn186,Gln185,Lys183,Arg141,Thr138,Tyr140 |
|  | ZINC_70706981 | 30.77  nM | -10.25 | 1 | Val110,**Lys85**,Val135,Val70,Leu132,Ala83,Glu97,Phe201,Asp200,Cys199,Asn186,Lys183,Gln185,Leu188,Tyr140,Thr138,Arg141 | -10.00 | 2 | **Asn64**,Gly65,Val110,Gly68,**Lys85**,Gly63,Val135,Val70,Ile62,Tyr134,Ala83,Phe67,Ser66,Asp200,Lys183,Gln185,Leu188,Thr138,Arg141 | -191.317 | 2 | **Asn64**,Gly68,**Lys85**,Val87,Val110,Leu132,Val70,Gly63,Leu188,Ala83,Val135,Ile62,Tyr134,Thr138,Arg141,Tyr140,Gln185,Lys183,Phe67,Ser66,Asp200,Gly65,Cys199 |
|  | ZINC_70706982 | 20.07  nM | -10.50 | 1 | Asn64,**Val135**,Ile62,Tyr134,Val70,Ala83,Leu132,Lys85,Glu97,Asp200,Cys199,Asn186,Gln185,Leu188,Arg141 | -9.9 | 2 | **Asn64**,Gly65,Gly68,**Lys85**,Val135,Gly63,Val70,Ile62,Tyr134,Ala83,Phe67,Asp200,Lys183,Gln185,Leu188,Thr138,Arg141 | -192.292 | 2 | **Asn64**,Gly65,Val110,Gly68,**Lys85**,Val87,Cys199,Asp200,Ser66,Phe67,Leu132,Val70,Gly63,Ala83,Ile62,Tyr134,Val135,Leu188,Arg141,Thr138,Tyr140,Gln185,Lys183,Asp200,Ser66,Phe67,Cys199 |
|  | **ZINC_70707119** | 190.48  pM | -13.26 | 5 | **Asn64**,**Val135**,Ile62,Tyr134,Val70,Ala83,Leu132,Asp200,Cys199,Asn186,Leu188,**Gln185**,Tyr140,**Thr138**,**Arg141** | -9.7 | 2 | **Asn64**,Gly65,Gly68,Val110,**Lys85**,Gly63,Val135,Val70,Ile62,Tyr134,Ala83,Phe67,Ser66,Asp200,Lys183,Gln185,Leu188,Thr138,Arg141 | -200.768 | 3 | Ile62,Gly63,Ala83,Val70,**Asn64**,Leu132,Gly65,**Lys85**,Gly68,Ser66,Val87,Phe67,Leu132,Asp200,**Lys183**,Cys199,Val110,Gln185,Leu188,Val135,Thr138,Arg141,Tyr134 |
|  | ZINC_70707134 | 3.5  nM | -11.54 | 4 | Asn64,**Val135**,Ile62,Tyr134,Ala83,Leu132,Asp200,Cys199,Leu188,Asn186,**Gln185**,Tyr140,**Thr138**,**Arg141** | -10.5 | 2 | **Asn64**,Gly65,Gly68,**Lys85**,Val135,Gly63,Val70,Ile62,Tyr134,Ala83,Phe67,Asp200,Lys183,Gln185,Leu188,Thr138,Arg141 | -192.522 | 2 | Asn64,Val110,Gly65,Cys199,Asp200,Ser66,Phe67,Gly68,Lys85,Val87,Leu132,Val70,Gly63,Ala83,Ile62,Tyr134,Val135,Leu188,Gln185,Asn186,Lys183,Arg141,Thr138,Tyr140 |
|  | ZINC_70707655 | 7.13  nM | -11.11 | 2 | **Asn64**,Gly68,**Lys85**,Val70,Val135,Ile62,Ala83,Phe67,Asp200,Cys199,Asn186,Lys183,Gln185,Leu188,Thr138,Arg141 | -10.2 | 2 | Asn64,Gly65,Gly68,Lys85,Val135,Gly63,Val70,Ile62,Tyr134,Ala83,Phe67,Ser66,Asp200,Lys183,Gln185,Leu188,Thr138,Arg141 | -201.986 | 3 | Ile62,Gly63,Ala83,Val70,**Asn64**,Leu132,Gly65,**Lys85**,Gly68,Ser66,Val87,Phe67,Asp200,**Lys183**,Cys199,Val110,Gln185,Leu188,Tyr140,Thr138,Val135,Arg141,Tyr134 |
|  | ZINC_95100194 | 1.39  uM | -7.99 | 5 | Asn64,Val110,**Ser66**,Phe67,**Lys85**,Gly68,Leu132,Ala83,Ile62,**Val135**,Leu188,Asn186,Asp200,**Arg141**,Tyr140 | -8.4 | 6 | Gly65,Val110,Gly68,**Lys85**,Val135,Gly63,Val70,Leu132,Ile62,Tyr134,Tyr83,Phe67,Glu97,Cys199,**Asp200**,Phe201,Ser203,Asp181,**Lys183**,Asn186,Gln185,Leu188,Thr138,**Arg141**,Tyr140 | -187.598 | 7 | Ser203,Phe201,**Asp200**,Cys199,**Ser66,Phe67**,Val110,Leu188,Gly65,Glu97,Met101,**Lys85**,Gly68,Leu132,Leu130,Val70,Ala83,Asn64,Gly65,Gly63,Ile62,**Arg141**,**Thr138**,Tyr140,Gln185,Arg144,Asn186,Lys183,**Ser219**,Cys218,Asp181 |
